# Introducing exceptional growth mining—Analyzing the impact of soil characteristics on on-farm crop growth and yield variability

**DOI:** 10.1371/journal.pone.0296684

**Published:** 2024-01-29

**Authors:** Puck J. A. M. Mulders, Edwin R. van den Heuvel, Pytrik Reidsma, Wouter Duivesteijn

**Affiliations:** 1 Control Systems Technology, Department of Mechanical Engineering, Technische Universiteit Eindhoven, Eindhoven, the Netherlands; 2 Stochastics, Department of Mathematics and Computer Science, Technische Universiteit Eindhoven, Eindhoven, the Netherlands; 3 Plant Production Systems, Department of Plant Sciences, Wageningen University, Wageningen, the Netherlands; 4 Data Mining, Department of Mathematics and Computer Science, Technische Universiteit Eindhoven, Eindhoven, the Netherlands; BOKU: Universitat fur Bodenkultur Wien, AUSTRIA

## Abstract

Sustainable intensification of agriculture requires understanding of the effect of soil characteristics and nutrient supply on crop growth. As farms are increasing in size by acquiring small fields from various farmers, the soil characteristics and nutrient supply might be very different from field to field, while at the same time specific soil properties might limit the nutrient uptake. As a result, there might be a large number of heterogeneous reasons why crop growth varies significantly. New data analysis techniques can help to explain variability in crop growth among fields. This paper introduces Exceptional Growth Mining (EGM) as a first contribution. EGM instantiates the data mining framework Exceptional Model Mining (EMM) such that subgroups of fields can be found that grow exceptionally in terms of three growth parameters (high/low maximum growth, steep/flat linear growth and early/late midpoint of maximum growth). As second contribution, we apply EGM to a case study by analyzing the dataset of a potato farm in the south of the Netherlands. EGM consists of (i) estimating growth curves by applying nonlinear mixed models, (ii) investigating the correlation between the estimated growth parameters, and (iii) applying EMM on these growth curve parameters using a growth curve-specific quality measure. By applying EGM on the data of the potato farm, we obtain the following results: 1) the estimated growth curves represent the variability in potato tuber growth very well (*R*^2^ of 0.92), 2) the steepness of the growth curve has a strong correlation with the maximum growth and the midpoint of maximum growth, and the correlation between the midpoint of maximum growth and maximum growth is weak, 3) the subgroup analyses indicate that: high values of K correspond to high maxima; low values of K correspond to low maxima, steep growth curves’, and a late midpoint of halfway growth; Mg influences the midpoint of the growth curve; values of B are higher on dry soils with high tuber growth, while low values of B are found on wet soils with high tuber growth; high values of Zn, Mn, and Fe are found in subgroups with low tuber weight, probably related to the soil’s low pH. In summary, this paper introduces EGM to obtain understanding in crop response to soil properties and nutrient supply. In addition, EGM provides a way to analyze only small parts of a large dataset, such that the impact of soil factors on growth can be analyzed on a more detailed level than existing methods.

## Introduction

Sustainable intensification of agriculture aims to increase crop yield and its economic returns per unit of time and land, without putting a strain on soil and water resources or the integrity of associated non-agricultural ecosystems [1, 2]. In practice, this calls for farm management that is adjusted in such a way that the soil’s capacity to provide water and nutrients for crop growth is maintained or enhanced, such that sustainability is ensured in terms of soil quality. To realize this, responsible nutrient supply is required: the crop demand must be met without any excess or deficiency throughout the season. In order to be able to respond to the crop’s exact needs, it is necessary to understand how the soil nutrient content affects crop growth. Although much research has been done on this topic over the years, estimating crop response to soil and fertilizer nutrient supply and applying subsequent management requirements remains a challenge [3, 4].

Within a farm, crop growth and yield highly varies. Understanding how soil nutrient content influences these differences in growth is crucial, especially because farms in the Netherlands and similar countries are increasing in size [5] due to the rental of a large number of small fields that were all managed by different farmers and thus all have a different past. As a result, the soil nutrient supply, and hence the crop’s fertilizer nutrient demand, might be very different from field to field, also given the observation that nutrient uptake might be limited due to specific soil properties. As an increasing number of farmers collect data, opportunities arise: new agronomic research methods can contribute to understanding the crop’s response to soil and fertilizer nutrient supply for the purpose of sustainable intensification [2, 6].

In response to this opportunity, data-driven methodologies are developed to analyze crop growth and yield. Some methods aim to divide fields into management zones that can be used for variable fertilizer application or yield predictions [7–9]. Other methods aim to explain and predict crop growth and/or yield variability and use only one model that is applied across all fields [10–12]. Since crop behavior will likely vary across the heterogeneous conditions between (sub-)fields within a farm, it is likely that such one-size-fits-all modeling will deliver suboptimal results. These between-field variations in soil conditions make modeling crop growth and yield a complicated task, as the reasons why crop growth is optimal or reduced might be different across fields. Therefore, it would be more appropriate to analyze available data with methods that explicitly cater for multiple kinds of crop growth behavior. Crucially, multiple kinds of behavior can result from multiple overlapping causes. It would therefore be beneficial for a method to automatically detect deviating growth, and cater for multiple overlapping phenomena occurring simultaneously.

**Fig 1:**
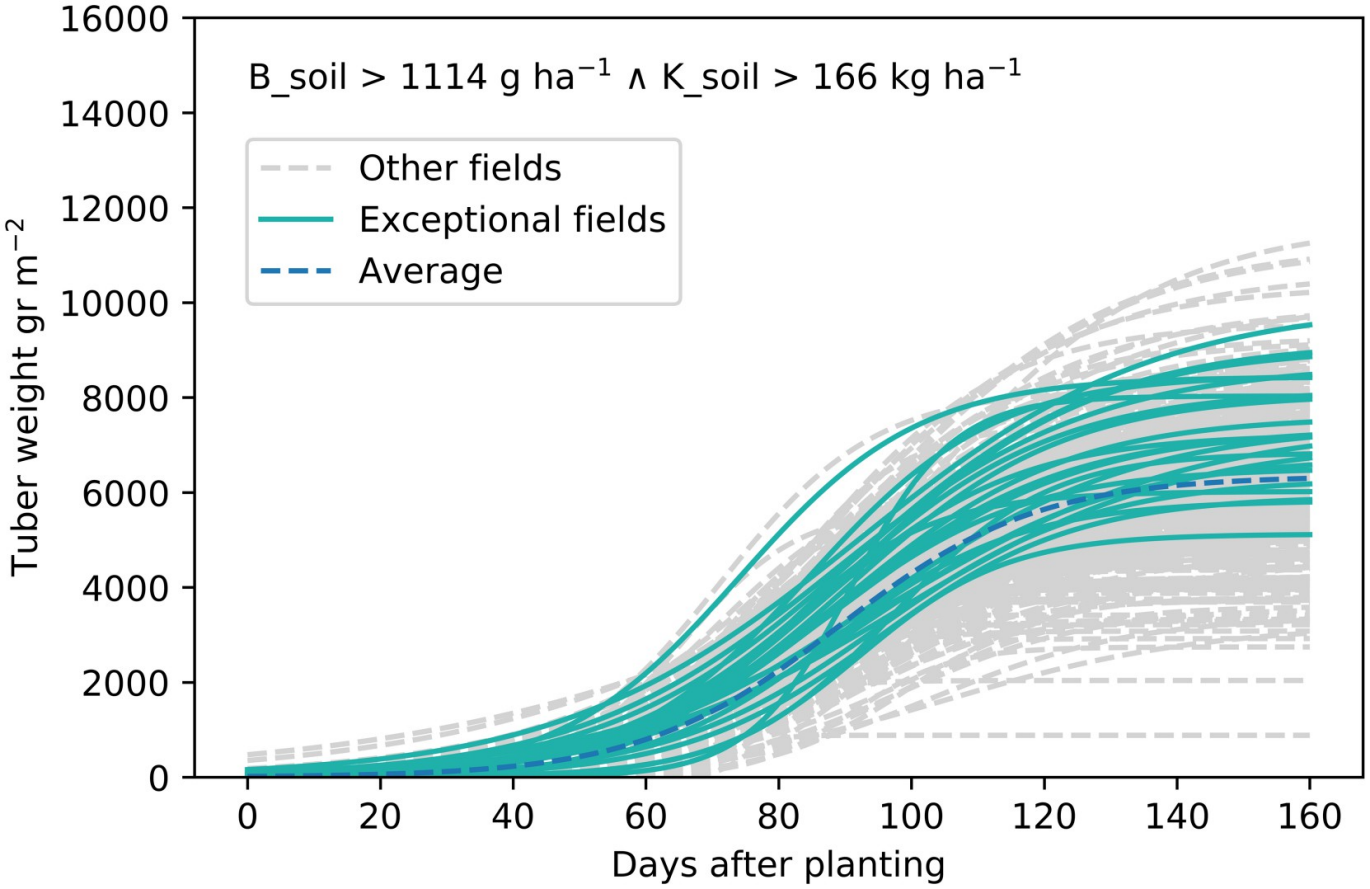
Example of a subgroup with exceptional growth. On average, the fields in the subgroup have a higher maximum tuber weight.

In this paper, we introduce Exceptional Growth Mining (EGM) as a new method to analyze on-farm collected data. It employs mixed models [13] to accurately model the growth curves for all fields. These growth curves are then embedded in the search strategy of Exceptional Model Mining [14, 15], a local pattern mining technique striving to find coherent subgroups of the dataset that display exceptional behavior. The result of EGM will be a list of subgroups of fields: each subgroup is defined by soil properties (such as the conditions given in Fig 1), and displays growth curves that deviate from the general behavior in a specific way (such as the highlighted curves in Fig 1). EGM can find subgroups where growth leads to an exceptionally high/low maximum growth, subgroups where the growth curve is exceptionally steep/flat, and subgroups where the midpoint of maximum growth is reached exceptionally early/late after planting. The resulting subgroups provide new understandings of growth and yield variability: it generates new hypotheses on how soil factors impact growth on a more detailed level than existing methods can, and hence ought to stimulate further agronomic research.

We apply EGM on a dataset of a potato *(Solanum tuberosum L.)* farm in the south of the Netherlands [12]. Starting from 2015, the farmer has analyzed the soil to obtain the extractable levels of mineral nutrients for the potato plants for approximately 100 fields per year. Throughout the growing season, the farmer has sampled potato plants on each field to monitor the growth. The potato growth varies highly between fields; on some fields, more than 80 ton ha^−1^ yield was obtained, while other fields obtained no more than 40 ton ha^−1^ yield, making it an appropriate case study to analyze with EGM.

### Main contributions

The main contributions of this work are:

- introduction of EGM: a new data analysis technique to discover various reasons for reduced or optimal plant growth by applying Exceptional Model Mining on growth curve parameters, resulting in a set of subgroups;
- analysis of the growth parameters: how are the maximum, steepness, and midway point of growth related;
- result list of subgroups defined (mostly) in terms of soil nutrients, displaying exceptional potato growth applied on the dataset of the mentioned farm.

## Materials and methods

**Fig 2:**
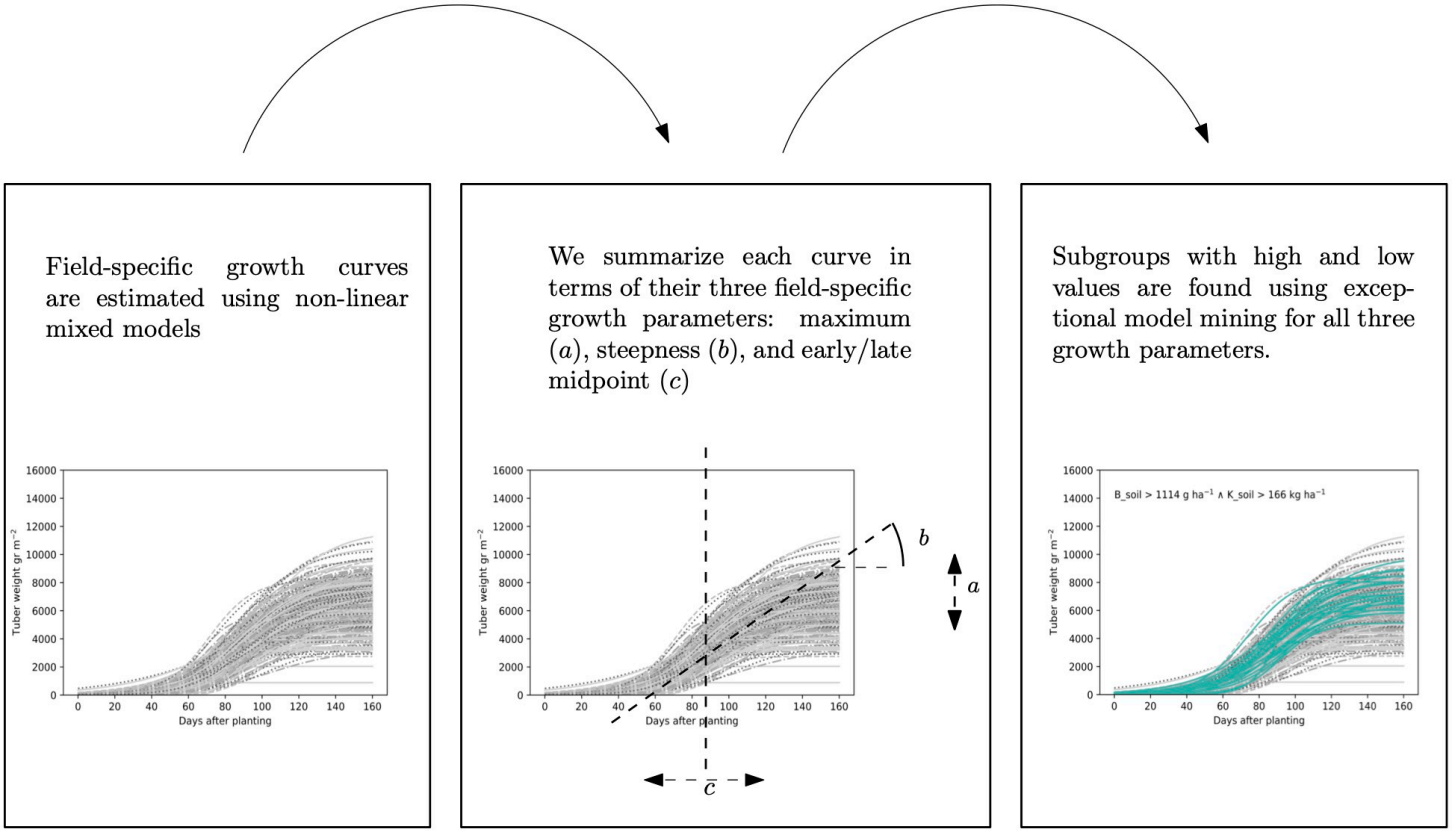
Overview of the three steps of EGM.

The goal of *Exceptional Growth Mining* (EGM) is to find subgroups of fields that grow exceptionally, such as the one in Fig 1. EGM can be applied if longitudinal growth data are collected, as well as some variables that could possibly influence growth. Growth curve analysis provides insights in the growth dynamics during the growing season, allowing us to understand how soil nutrients influence specific properties of growth (e.g., early/late midpoint, high/low maximum yield, and steep/flat curve), and how these properties interact. This is useful for farm management: for example, a field which has a very steep growth will be fully grown relatively soon, and thus could be planted later. Roughly, EGM consists of (i) estimating growth curves, (ii) investigating the correlation between the estimated growth parameters, and (iii) applying Exceptional Model Mining [14, 15] on these growth curves. In EGM, we develop a quality measure for exceptional model mining, which is specifically designed for the field-specific growth curves. As applying EGM can result in an overwhelmingly long list of subgroups, we in addition show how to automatically select the most relevant ones. An overview of the three steps of EGM can be found in Fig 2.

In order to apply EGM, it is necessary to have access to a detailed dataset of longitudinal growth data of one ore more plant characteristic. In addition, of each sampling location, details of soil and/or management variables should be available in order to discover heterogeneous reasons for variability in growth.

In the upcoming sections, we start with providing an overview of the data of the case study. Secondly, we explain how to estimate field-specific growth curves. Thirdly, we discuss Exceptional Model Mining, followed by how to turn EMM into EGM, where we explain how to turn the growth curves into targets and explain the growth-specific quality measure. Finally, we explain how to select the most interesting subgroups found by EGM.

### Data

The data of the case study were collected on potato farm Van den Borne Aardappelen in the south of the Netherlands. The owner of this farm, Jacob van den Borne, has given us permission to use these data. No permits were required to access the fields, as the data were collected by himself, his employees and/or students who did an internship at the farm. The data from 2015 to 2018 were used. Each year, potato plants were sampled five to seven times per growing season by the farmer on approximately 100 fields. The main goal was to monitor potato growth throughout the season. Each sample round, the farmer picked three potato plants and weighed the tubers. The total tuber weight of these three plants were used to calculate the tuber weight in grams per squared meter:

$$\begin{array}{c} y\left(\text{gr}\;{\text{m}}^{-2}\right)=\frac{\tilde{y}\left(\text{gr/3}\;\text{plants}\right)*1.333}{\text{planting}\;\text{distance}\;\text{(cm)}*3/100} \end{array}$$

The potatoes were sampled at the median of the electrical conductivity of the field. The electrical conductivity of a field was measured using a DUALEM-21s: it measures conductivity based on soil properties such as the amount of salt, water, and soil compaction. Three plants were taken per sampling moment; prior work showed that relatively small number suffices since the tuber growth of these three plants has a strong representative relation to yield at field level [12].

At the first sampling round, soil samples were taken and analyzed by Eurofins [16], resulting in the extractable levels of mineral nutrients. These soil sample analyses included the levels of the main soil nutrients N (Nitrogen), P (Phosphor) and K (Potassium) and many more macro and micro nutrients, but unfortunately these analyses did not include any information of the soil’s pH and Soil Organic Matter (SOM). There is however a relation between pH and SOM and analyzed micro nutrients Mn, Fe and Zn [17], which allows us to implicitly analyze the effect of pH and SOM on crop growth. Based on the potato’s needs, Eurofins provided a range for each nutrient in which the nutrient amount should lie. In addition, a category depending of the level of dryness (dry, average, and wet) and nutrient richness (rich, average, and poor) was assigned to each field based on the expert knowledge of the farmer’s father. It was also known which crop was cultivated before the potatoes and whether the field was infected with nematodes. For a complete overview of the data used in this analysis, see S1 Fig and S1 Table.

The farmer mainly cultivates Fontane potatoes for the French Fries industry. The area in which the farmer operates consists mainly of sandy soils. The climate is a moderate maritime climate (Köppen classification Cfb). About 22 km away from the farm, a meteorological station captured weather data. Based on the weather data between 2008 and 2018, the solar radiation is about 3.1 MJ m^−2^ and the average temperature 6.6°C during winter (December, January, and February), slowly increasing to 17.8 MJ m^−2^ and 23.5°C in the summer (June, July, and August). Yearly precipitation is about 742.3 mm on average.

Four years of data were used (2015–2018). In particular, 2016 and 2018 experienced extreme weather. 2016 was a very wet year, where in June about 200 mm of rain fell, resulting in fields with rotten potatoes due to water excess. On the other hand, 2018 was extremely dry. Almost no rain fell in June and July, resulting in a long period of drought with high temperatures. The years 2015 and 2017 had preferable weather conditions: there was no excess or lack of water, and temperatures were close to the average, resulting in high yields. We report this weather data here pure to provide context to the reader: no meteorological parameters were available to the curve estimation model that represents the potato growth, and to the search algorithm that creates candidate subgroups.

### Estimating growth curves

We started by modeling growth curves for all fields. The field-specific growth curves were estimated using mixed models [13]. Mixed models have mixed effects consisting of two components: fixed effects that represent the yearly average growth curve, and random effects that represent the field-specific deviation from this yearly average growth curve exploiting the variance-covariance structure of the repeated data. Mixed models are able to model observations taken on non-equidistant time intervals: this holds for measurements taken on the same subject, but it also allows different subjects to be measured at different moments in time. In addition, mixed models can handle missing data well when the missingness mechanism is not “missing not at random” and longitudinal profiles can be estimated even if a subject has only a few observations by employing the variance-covariance structure of the entire dataset. As a result, mixed models use data efficiently, as almost all data can be used to estimate longitudinal growth profiles. The growth curve was modeled based on the shape of growth over time and the quality of its fit was evaluated with *R*^2^ (linear regression on observed and non-linear mixed model predicted observations). It was important that the *R*^2^ was high, as the growth curves were the basis of the further analysis and the validity of the results relied on the quality of fit.

For the case study of the potato farm, we described the tuber weight as a function of days after planting. The tuber weight is described with an s-curve, as tuber growth is s-shaped [18], for which we use the logistic growth curve [19]. The missingness mechanism can reasonably be assumed to be a combination of Missing Completely At Random (MCAR) and Missing At Random (MAR). Some data is missing due to simple recording mistakes, which is MCAR. Some of the missingness resulted from the yield on a particular field being completely destroyed due to extreme weather. In those cases, the tuber weight was registered as zero, after which the farmer stopped visiting that field. Here, the missingness depends on meteorological variables that are not present in the dataset, but the missingness probability depends on values for those variables. This is MAR missingness, although the column on whose values the missingness probabilities depend are not part of the dataset itself. We chose a multiplicative error term, which occurs when growth is active [20], such that

$$\begin{array}{c} y_{ijk}^{TW}=\frac{\text{exp}(a_{ik})}{1+\text{exp}\{-b_{ik}\cdot \left(t_{ijk}-c_{ik}\right)\}}\text{exp}\left(e_{ijk}\right) \end{array}$$ (1)

with $y_{ijk}^{TW}$ the tuber weight on field *i* at standardized growth day *j* measured from the day of planting in year *k* (divided by its standard deviation for numerical purposes), where time is reset to zero each year, *e*_*ijk*_ the residual i.i.d. $N(0,\sigma^{2})$, and:

$$\begin{array}{c} \begin{array}{cc} a_{ik} & =\alpha_{0k}+\sigma_{\alpha }a_{i}\\ b_{ik} & =\text{exp}\{\beta_{0k}+\sigma_{\beta }b_{i}\}\\ c_{ik} & =\gamma_{0k}+\text{exp}\left\{\sigma_{\gamma }\right\}c_{i} \end{array} \end{array}$$ (2)

**Fig 3:**
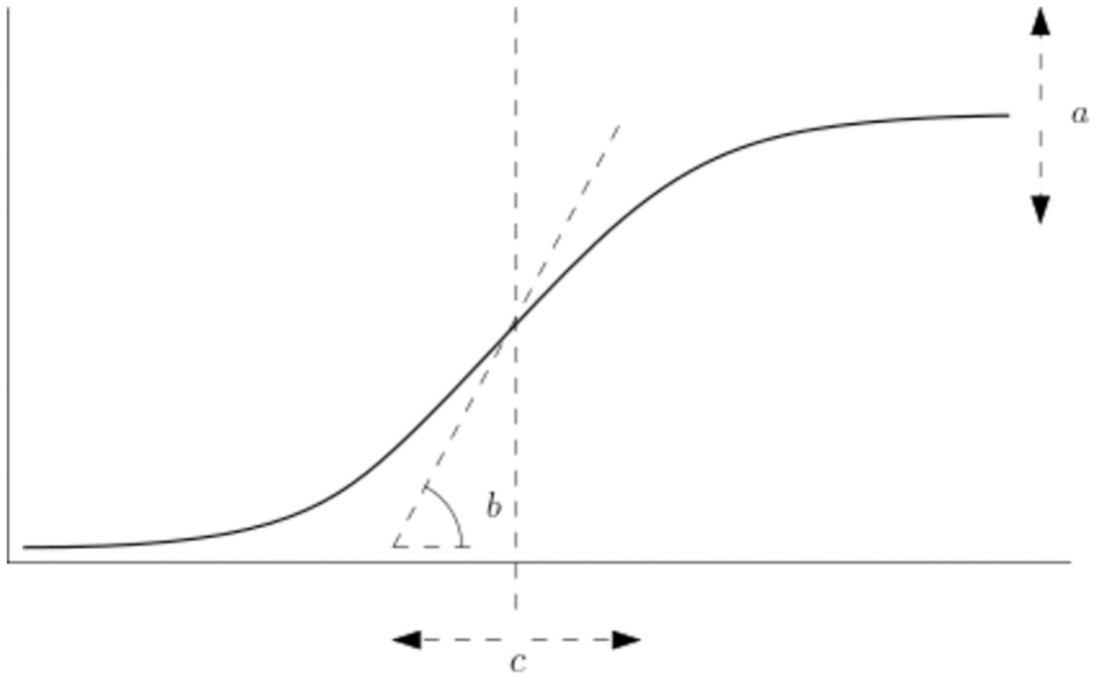
Transformations on the growth curve. Positive values of *a* result in a higher maximum and negative values of *a* result in a lower maximum. Positive values of *b* refer to a steeper curve, while negative values of *b* make the growth curve flatter. Positive values of *c* delay the moment of halfway maximum growth, and negative values advance the moment when halfway maximum growth is reached.

Here, *α*_0*k*_, *β*_0*k*_, and *γ*_0*k*_ are year-specific mean parameters, *σ*_*α*_, *σ*_*β*_, and *σ*_*γ*_ are standard deviations, where we assume that the variance between years is constant. Exponentiation ensures that *σ*_*γ*_ > 0 and *b*_*ik*_ > 0, which makes *b*_*ik*_ essentially lognormally distributed. *a*_*i*_, *b*_*i*_ and *c*_*i*_ are the random effects, which correspond to an interpretable transformation (see Fig 3):

*a*_*i*_ is the field-specific deviation from the yearly average maximum tuber growth;
*b*_*i*_ corresponds to the field-specific deviation in steepness of the linear growth;
*c*_*i*_ refers to the moment in time when half the maximum tuber growth has been produced.

The random effects *a*_*i*_, *b*_*i*_, and *c*_*i*_ are multivariate normally distributed:

$$\begin{array}{c} \left(\begin{array}{c} a_{i}\\ b_{i}\\ c_{i} \end{array})∼N((\begin{array}{c} 0\\ 0\\ 0 \end{array}),(\begin{array}{ccc} 1 & \rho_{12} & \rho_{13}\\ \rho_{12} & 1 & \rho_{23}\\ \rho_{13} & \rho_{23} & 1 \end{array})\right) \end{array}$$ (3)

The covariance matrix of the random effects contains the correlations between the random effects, with *ρ*_12_ the correlation between *a* and *b*, *ρ*_13_ the correlation between *a* and *c*, and *ρ*_23_ the correlation between *b* and *c*. We estimated the log likelihood function of this model with adaptive Gaussian Quadrature using the NLMIXED procedure of SAS [21]. For the used SAS code, see S1 Appendix.

### Exceptional model mining

Exceptional Model Mining (EMM) [14, 15] is a local pattern mining framework, designed to find interesting subgroups in datasets. Subgroups are deemed interesting, if they possess two characteristics:

1. they must be *interpretable*. This is typically covered by allowing subgroups to only be those subsets of the datasets at hand, often defined by a conjunction of relevant attributes of the dataset;
2. they must be *exceptional*. This is typically gauged in terms of unusual parameter values in some kind of model fitted over the variables of the dataset in which we are interested, the so called targets.

Let Ω denote our dataset encompassing *N* records of the form *r* = (*d*_1_, …, *d*_*k*_, *t*_1_, …, *t*_*m*_). Whenever we want to refer to a specific record of the dataset (or element thereof), we do so by superscript index *l*: the *l*^th^ observation is $r^{l}=\left(d_{1}^{l},\ldots ,d_{k}^{l},t_{1}^{l},\ldots ,t_{k}^{l}\right)=\left(d^{l},t^{l}\right)$. The attributes *d*_1_, …, *d*_*k*_ are the *descriptors* of the dataset. Each descriptor *d*_*i*_ takes values from a domain $D_{i}$, and hence $\forall_{l=1}^{N}d_{i}^{l}\in D_{i}$. This domain can be any kind of set: numeric, binary, or nominal, for example. In our case study, we have descriptor *d*_1_ being the K soil content, where $\forall_{l=1}^{N}d_{1}^{l}\in D_{1}$, with $D_{1}\subseteq R_{+}$; and descriptor *d*_2_ being the dryness of the field, where $\forall_{l=1}^{N}d_{2}^{l}\in D_{2}$, with $D_{2}=\left\{\text{Dry},\text{Average},\text{Wet}\right\}$. The attributes *t*_1_, …, *t*_*m*_ are the *m* *targets* of the dataset; they are used to *evaluate* subgroups and, hence, govern *exceptionality*. In our case, all targets are numeric, and thus $\forall_{l=1}^{N}t_{i}^{l}\in R$ with *i* = 1, …, *m*. The targets are related to the tuber weight throughout the season, which we will replace by the growth parameters/random effects *a*, *b*, and *c* as estimated in Eq (2), which will be further discussed in the next section.

We then use $D_{1}\times \ldots \times D_{k}$ to define our *description language* D, which is a subset of P(D), being the powerset of D, i.e., collection of all subsets of D.

**Definition 1 (Description language)** D⊆P(D)

Each $\bar{D}\in D$ is called a *description*. According to the formal definition, $\bar{D}$ can be any subset of P(D). In practice, we want to relate the description to the interpretation of individual descriptors. Hence, we let D search through the space of conjunctions of conditions on *d*_1_, …, *d*_*k*_. For instance, if *d*_1_ is the K soil content and *d*_2_ the dryness of the field, then $\bar{D}$ can be the subset $\left\{r^{l}\in \Omega \;|\;d_{1}^{l}>200\;\text{kg}\;{\text{ha}}^{-1}\wedge d_{2}^{l}=\left\{\text{Average}\right\}\right\}$ of P(D).

We define the subgroup that follows from description $\bar{D}$ as $G_{\bar{D}}$:

**Definition 2** $G_{\bar{D}}=\{r^{l}=\left(d^{l},t^{l}\right)\in \Omega |d^{l}\in \bar{D}\}$

From now on, if we refer to a subgroup, we omit $\bar{D}$ if no confusion can arise, and simply call it *G*.

A *quality measure* is a function that quantifies for each description $\bar{D}$ in the description language D how exceptional the corresponding subgroup $G_{\bar{D}}$ of Ω is. In our case, the quality measure uses the targets of the subgroup to calculate the quality of the subgroup (see the next section for more details, specifically Eqs (6) and (7)) and is used to measure its interestingness.

**Definition 3 (Quality measure)** A *quality measure* is a function ϕ:D→R that assigns a numeric value to a subgroup $G_{\bar{D}}$ of Ω induced by $\bar{D}$.

From this formal definition, a quality measure can represent basically anything about the subgroup. However, philosophically, in EMM, the quality measure should be designed to capture the level of exceptionality in a specific kind of interaction between the targets, as dictated by the chosen model class. In Exceptional Growth Mining, this type of interaction is gauged by unusual parameters of the growth curves.

Beam search is a heuristic algorithm, delivering a top-*q* of the most exceptional subgroups it found during its search process [15]. Beam search holds the middle ground between a purely greedy approach and an exhaustive algorithm (which is computationally unaffordable). It builds up subgroups level-wise, considering all conditions on a single attribute on the first level of the search. For numeric variables, thresholds are created automatically, and all categories of categorical variables are considered. How these thresholds are estimated, can be altered and optimized as well [22], but in our case, we stick to the traditional approach of the beam search algorithm, as we discuss in the upcoming paragraph. The top-*w* (for *beam width*) most exceptional subgroups are stored as the *beam* for the next level. On every subsequent level, those subgroups that ended up in the beam are retrieved, and refined (by conjoining all possible new conditions on single attributes to the existing description) into new candidate subgroups for the next level; the *w* best of those are stored as the new beam for the next level. The search terminates after a fixed number *d* (for *search depth*) of levels. Here, we chose the largest *d* in such a way that most subgroups have more than 15 members. This choice is rather arbitrary; there is no consensus in literature on how to properly set the parameter. Result variation through varying this parameter strongly depends on the dataset at hand, and its underlying structure. The choice of *d* is a trade-off between model stability and subgroup specificity: the lower we set *d*, the more specific are the subgroups that we will find, but also the fewer fields are used to estimate its model (thus increasing uncertainty). If a user would prefer to find more stable models or more specific subgroups, this parameter can be varied at will.

Exhaustive algorithms for Exceptional Model Mining also exist. We argue that deploying them here would do more harm than good. As one can see from S1 Table, our dataset encompasses several continuous variables. The continuous variables have a high-cardinality; repeated measurements are scarce. As a consequence, if the subgroup ‘K_soil ≤105’ were to be deemed exceptional by EGM, then subgroups ‘K_soil ≤104’, ‘K_soil ≤102’ and ‘K_soil ≤100’ will also be deemed exceptional by EGM. Thus, exhaustive algorithms will pollute the result set of an EGM run with many reinventions of very few wheels. One could consider empirically evaluating the extent to which this is a problem in real-life, if it were not for the fact that S1 Table also displays that the continuous variables are mostly weakly correlated. Thus, exhaustive search through the space of subgroups defined as conjunctions of even a limited number of conditions on these attributes, likely gives the algorithm access to a substantial subset of *all possible subsets of fields in our dataset*. Hence, the severity of this problem cannot be computed in a reasonable amount of time.

The beam search algorithm pseudocode can be found in [15]. Our implementation is based on an existing EMM implementation [23]; our source code can be found (along with data from this paper) at dx.doi.org/10.6084/m9.figshare.24592506.

### Quality measures

Each field has multiple observations or records throughout the growing season, resulting in dependence between the records. Therefore, after applying Eq (2), we replace the tuber weight measurements by the estimated random effects *a*, *b*, and *c*, reducing our target space to a multivariate normal distribution, in which all records are independent. As a result, each field *i* is now a record *l* in the dataset. We search for subgroups in each of the random effects separately. From here on, *u* refers to any of the random effects *a*, *b*, or *c* as estimated by Eq (2). For each of the random effects *a*, *b*, and *c*, we have

$$\begin{array}{c} u∼N(0,\sigma_{u}) \end{array}$$ (4)

Any subgroup *G* under consideration is a subsample from that distribution. Hence, whether we look at the entire dataset or a specific subgroup, we retrieve a sample *G* of a finite size *n* from the distribution in Eq (4). From this sample, we derive an estimate of the two main parameters ${\hat{\mu }}_{G}^{u}$ being the mean of *u* and ${\hat{\sigma }}_{G}^{u}$ being the standard deviation of *u*. Depending on the random effect, these parameters indicate how the subgroup deviates from the average growth curve.

A subgroup is considered exceptional when the found subgroup (with a clear description) does not have zero mean anymore. This type of exceptionality is captured by the *t*-test statistic, which can then be used to derive our quality measure. We can independently put our parameter estimates to a standard *t*-test statistic for each subgroup *G*, computing the test statistics:

$$\begin{array}{c} t_{G}^{u}=\frac{{\hat{\mu }}_{G}^{u}\sqrt{n}}{{\hat{\sigma }}_{G}^{u}} \end{array}$$ (5)

Hence, positive values of $t_{G}^{u}$ indicate a positive mean, and negative values of $t_{G}^{u}$ indicate a negative mean. Next, we define the following two quality measures to use for the growth curves (GC) for EMM, where Eq (6) searches for subgroups with positive values of *u*, and Eq (7) searches for subjects with negative values of *u*.

$$\begin{array}{c} \varphi_{{\text{GC}}_{h}}^{u}\left(G\right)=t_{G}^{u} \end{array}$$ (6)

$$\begin{array}{c} \varphi_{{\text{GC}}_{l}}^{u}\left(G\right)=-t_{G}^{u} \end{array}$$ (7)

These quality measures correspond to the demands of Exceptional Model Mining: the higher their output values, the more exceptional the behavior on target space in the desired direction.

For each subgroup, we calculate its average potato yield, in order to relate this yield to the growth parameters and subgroups. In addition, we check for each subgroup how many of the fields were irrigated and when the fields were planted. Because the farmer cultivates many small parcels, it takes over a month to plant all fields. As the planting period progresses, the temperature rises, which results in potato plants that grow faster, influencing the shape of the growth curve (as we expect a high *b*) and therefore possibly influencing the found subgroups. Therefore, for each year, the planting period is split into three sets: fields that are planted early, averagely, or late. For each subgroup, we check to which extent these management factors could overrule the found soil conditions.

Taking *u* as the target implies that we searched for shifts away from the yearly average growth curve, i.e., we aim to find subgroups that explained the within-year variability over all years, excluding year-specific effects and therefore excluding weather-specific effects. We verified this by checking in which years the fields of the found subgroups were cultivated.

### Filtering out Pareto-suboptimal subgroups

EGM could result in an overwhelmingly long list of exceptional subgroups. Inspired by the work on Skypatterns [24], we reduce the list through the following observations. The quality measures in Eqs (6) and (7) could be seen as a trade-off between the mean, standard deviation, and number of subjects within a group. For example, subgroups with a high mean could be interesting, because they yield so well on average. On the other hand, if such groups have a high standard deviation, they are not necessarily very reliable. In that sense, groups with a slightly lower mean but also a small standard deviation could be interesting also. This is influenced by the number of subjects within the subgroup as well: it is likely that a larger subgroup has a slightly larger standard deviation than a small group, but as the description holds for more subjects, this information is also useful. Therefore, we implement a Pareto front, capturing the trade-off between the mean, the standard deviation, and the number of subjects within the group. Following the work on Skypatterns [24], we report only those subgroups that lie on this Pareto front; i.e. in a postprocessing step of the beam search procedure, we keep only those subgroups that are not dominated by another subgroup in the space spanned by these three factors.

To this last idea, a similar approach was also explored in [25]. There, the authors map the observations from a dataset (what they call *objects*, and what in this paper would be the individual fields) into a (generic) Pareto space. Subsequently, the exceptionality of a subgroup is evaluated by the degree to which removal of its observations would affect the Pareto front. This enables statements on the “true” Pareto front: what would the Pareto front be when all observations are mapped into the Pareto space? Here, conversely, we map subgroups of our dataset into a (specific) Pareto space. Subsequently, statements on the “true” Pareto front cannot be made: this would require to map all possible subgroups into the Pareto space, and there are exponentially many more of those than there are observations in the dataset. Losing the capability to make such optimality statements is a sacrifice we must make w.r.t. the method of [25], because our agronomic setting gives a natural rise to a Pareto space composed of metrics that evaluate entire subgroups, which renders optimality in this space computationally intractable.

It is worth noting that the Pareto filtering we employ is asymmetric. On the one hand, the filtering discards subgroups that we know to deem Pareto-suboptimal, because they are dominated by other subgroups that we have found with our EGM search algorithm. On the other hand, the filtering makes no statement on the optimality of the retained subgroups: it is possible that these subgroups themselves are also dominated by other subgroups that the EGM algorithm has not found. Hence, the Pareto front can be used to filter out suboptimal subgroups, but optimality of the remaining subgroups cannot be certified. The careful user of this filtering should refrain from overclaiming.

## Results

In the previous section, we introduced EGM. In the upcoming section, we present the results after applying EGM on the case study of the potato farm. Data underlying these results, as well as source code employed to obtain the results, can be found at dx.doi.org/10.6084/m9.figshare.24592506.

### Growth curves

**Fig 4:**
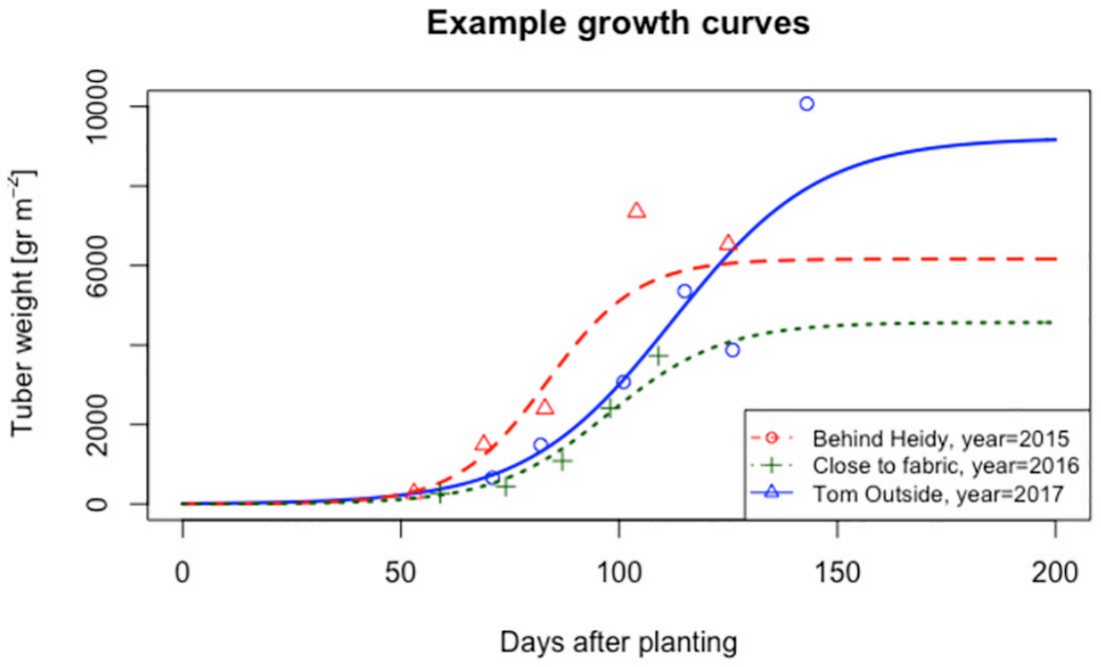
Example of three field-specific growth curves.

The basis of our analysis is formed by the field-specific growth curves. The *R*^2^ of the growth curves is 0.92: the fit of the mixed models is thus very high (cf. Fig 4). This implies that the estimated growth curves represent the data very well and we can draw conclusions using the growth curves.

Random effects *a* and *c* have relatively weak positive correlation (cf. Table 1). This implies that maximum tuber weight does not strongly relate to the moment in time when tuber weight is half of its maximum weight. Random effect *b* on the other hand has a strong negative correlation with *a* and *c*: a steeper growth curve implies that the maximum tuber weight is likely lower and half of the maximum growth is reached sooner. Based on these correlations, we expect that the descriptions of the subgroups found for exceptional *a* and *c* are different, while the descriptions for exceptional *b* are a combination of descriptions found for *a* and *c*.

**Table 1:** Estimated correlations following from the covariance matrix of the random effects.

	*b*	*c*
*a*	-0.65***	-0.49***
*b*		-0.73***

Significance is indicated with *** for *p*-value <0.0001.

### Subgroups with exceptional growth

There are large differences in yearly average yield (Table 2) and it is therefore important to evaluate the distribution of years within the subgroup.

**Table 2:** Yields from fields.

Year	# fields	*μ* yield	*σ* yield
2015	71	63.5	12.5
2016	98	40.1	18.1
2017	83	54.0	11.1
2018	112	38.0	11.6
All	364	47.3	17.0

Columns indicate the year, the number of fields included in the dataset for that year, and the mean and standard deviation of the yield in ton ha^-1^.

When calculating the Pareto front for each combination of quality measure and random effect, the list of most exceptional subgroups is still quite long (see Table 3 for metadata, and S2–S7 Tables for the full lists of subgroups on the Pareto front). Therefore, we present a subset of all found subgroups by reporting the top-5 subgroups for both the high and low values of all random effects (30 subgroups in total). These top-fives can be found in Table 4 for high and low values of *a*, in Table 5 for high and low values of *b*, and in Table 6 for high and low values of *c*. These subgroups have the highest $\phi_{\text{GC}}^{u}$ while satisfying *n* > 15. In some cases, two subgroups lie on the Pareto front of which one is the subset of the other (for example, when the first subgroup contains fields with K_soil <100 kg ha^−1^ and the second subgroup consists of fields with K_soil <200 kg ha^−1^). If these are part of the top-5, we present only the subgroup with the most fields. In addition, we perform a post-processing step: if within one description, one descriptor is redundant, we remove that descriptor (e.g., K_soil <200 kg ha^−1^ ∧ K_soil <100 kg ha^−1^ becomes K_soil <100 kg ha^−1^). For a complete list of all subgroups that lie on the Pareto front, see S2–S7 Tables.

**Table 3:** Number of fields on the Pareto front for each quality and random effect combination.

Target	Number of subgroups on Pareto front
High *a*	46
Low *a*	67
High *b*	65
Low *b*	29
High *c*	38
Low *c*	53

**Table 4:** Top-5 Pareto-optimal subgroups when searching for exceptional values of *a*. See S2 and S3 Tables for a full listing of all Pareto-optimal subgroups.

Target	Description	$\phi_{{\text{GC}}_{h}}^{u}$	$\hat{\mu_{GC}^{u}}$	${\hat{\sigma }}_{G}$	Number of fields					Yield
					Total	2015	2016	2017	2018	
High *a*	B_soil >1113.6 ∧ K_soil >165.5	4.53	0.42	0.45	24	5	3	11	5	58.5
	Ca_soil >196.8 ∧ Zn_soil ≤2055.6	4.25	0.5	0.52	19	0	7	4	8	45.1
	K_soil >308.1 ∧ Zn_soil ≤2082.0	3.83	0.39	0.51	25	3	6	5	11	49.9
	Fe_soil >324.0 ∧ Fe_soil ≤444.0	3.66	0.3	0.56	47	10	12	12	13	49.9
	B_soil ≤366.0 ∧ Dryness = wet	3.54	0.34	0.48	26	7	1	6	12	50.2
Low *a*	Dryness = average ∧ Zn_soil >6868.8 ∧ Zn_soil ≤11569.2	4.83	-0.79	0.64	15	1	8	3	3	39.5
	S_soil ≤22.8 ∧ Dryness ≠ wet ∧ Fe_soil >446.4	4.66	-0.52	0.58	27	10	10	4	3	46.9
	K_soil ≤308.1 ∧ Dryness ≠ wet ∧ Nutrient_content = poor	4.62	-0.6	0.65	25	5	5	8	7	46.7
	N_soil ≤138.2 ∧ Dryness ≠ wet ∧ Nutrient_content = poor	4.58	-0.66	0.66	21	4	5	5	7	44.1
	Mn_soil >9234.0 ∧ K_soil ≤375.3	4.48	-0.48	0.67	38	8	10	8	12	45.6

Yield is reported in ton ha^-1^, N, P, K, Ca and Mg are reported in kg ha^-1^ and B, Fe, Mn and Zn are reported g ha^-1^.

**Table 5:** Top-5 Pareto-optimal subgroups when searching for exceptional values of *b*. See S4 and S5 Tables for a full listing of all Pareto-optimal subgroups.

Target	Description	$\phi_{{\text{GC}}_{h}}^{u}$	$\hat{\mu_{GC}^{u}}$	${\hat{\sigma }}_{G}$	Number of fields					Yield
					Total	2015	2016	2017	2018	
High *b*	B_soil >564.0 ∧ N_soil >174.2 ∧ K_soil ≤183.1	5.18	0.66	0.55	19	5	5	7	2	47.4
	S_soil >11.4 ∧ Dryness = average ∧ K_soil ≤166.2	5.15	0.53	0.63	37	8	12	9	8	45.2
	Dryness = average ∧ S_soil >10.0 ∧ K_soil ≤166.7	4.84	0.51	0.64	38	9	12	9	8	45.4
	K_soil ≤146.1 ∧ Dryness = average ∧ N_soil >101.6	4.62	0.5	0.51	22	8	4	6	4	50.1
	Dryness = average ∧ K_soil ≤274.7 ∧ B_soil >453.6	4.58	0.51	0.69	39	11	14	11	3	47.9
Low *b*	B_soil ≤564.0 ∧ Dryness = wet	3.43	-0.34	0.66	43	8	8	8	19	44.4
	Dryness = wet ∧ N_soil ≤89.8	3.00	-0.3	0.59	35	5	10	13	7	45.4
	Dryness = wet ∧ B_soil ≤750.0	2.99	-0.27	0.65	52	9	9	10	24	43.2
	Ca_soil >196.8 ∧ Zn_soil ≤2055.6	2.81	-0.35	0.55	19	0	7	4	8	45.1
	Dryness = wet ∧ Zn_soil ≤1290.0	2.79	-0.45	0.68	18	2	5	5	6	46.6

Yield is reported in ton ha^-1^, N, P, K, Ca and Mg are reported in kg ha^-1^ and B, Fe, Mn and Zn are reported g ha^-1^.

**Table 6:** Top-5 Pareto-optimal subgroups when searching for exceptional values of *c*. See S6 and S7 Tables for a full listing of all Pareto-optimal subgroups.

Target	Description	$\phi_{{\text{GC}}_{h}}^{u}$	$\hat{\mu_{GC}^{u}}$	${\hat{\sigma }}_{G}$	Number of fields					Yield
					Total	2015	2016	2017	2018	
High *c*	Fe_soil >324.0 ∧ P_soil >6.0	3.43	0.32	0.44	22	10	6	3	3	55.9
	Zn_soil >7294.8 ∧ B_soil ≤592.8	3.33	0.26	0.47	36	7	11	6	12	46.7
	K_soil ≤146.1 ∧ Ca_soil >14.7	3.01	0.37	0.53	18	0	10	1	7	41.1
	Mn_soil >1454.4 ∧ Mg_soil ≤141.5	2.93	0.28	0.58	36	7	11	6	12	44.7
	Zn_soil >3906.0 ∧ Mg_soil ≤133.2	2.77	0.27	0.47	24	4	8	3	9	41.5
Low *c*	N_soil >40.2 ∧ K_soil ≤234.0	3.81	-0.21	0.67	146	45	25	42	34	49.2
	N_soil >40.2 ∧ Mg_soil >202.2	3.71	-0.21	0.68	141	37	17	42	45	50.2
	Zn_soil ≤3906.0 ∧ Mg_soil >228.0	3.56	-0.24	0.68	104	27	21	24	32	49.8
	Fe_soil ≤324.0 ∧ K_soil ≤216.3	3.54	-0.24	0.70	109	20	30	31	28	48.9
	K_soil ≤146.1 ∧ B_soil >1405.2	3.38	-0.62	0.90	24	4	7	13	0	46.4

Yield is reported in ton ha^-1^, N, P, K, Ca and Mg are reported in kg ha^-1^ and B, Fe, Mn and Zn are reported g ha^-1^.

Only for low values of *a* and high values of *b* the search depth has been set to *d* = 3, while in all other cases *d* = 2. As a result, for most subgroups we have 15 < *n* < 60, with the exception of four out of five subgroups with low values of *c*: these subgroups consist over 100 fields and thus span a large part of the entire dataset. When increasing *d* such that *d* = 3, the resulting set of subgroups contains only very small subgroups (for most *G*_*D*_, *n* < 10).

#### Subgroup growth

We used *a*, *b*, and *c* as targets for our EGM framework in order to capture subgroups based on the within-year variability and thus excluding the year-specific average. Most subgroups contain fields of all four years. This indicates that the relations found between the growth parameters and soil circumstances are mostly independent of variability between years.

**Fig 5:**
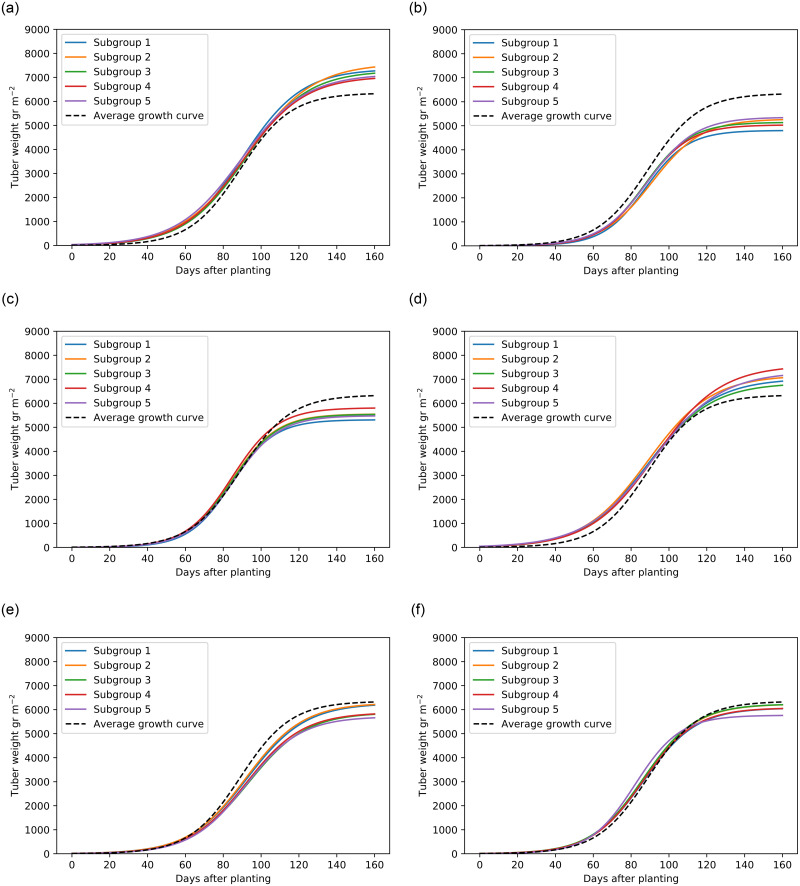
The average growth curve per subgroup for high and low values of *a*, *b*, and *c*. Subgroups correspond to and are enumerated in the same order as those listed in Tables 4 (for Figs 5A and 5B), 5 (for Figs 5C and 5D), and 6 (for Figs 5E and 5F). A: High *a*. B: Low *a*. C: High *b*. D: Low *b*. E: High *c*. F: Low *c*.

Fig 5 shows the average growth curve per subgroup compared to the average growth curve of all four years. High and low values of *a* clearly deviate from this average growth curve with about 1000 gr m^−2^ (corresponding to about 10 ton ha^−1^). Subgroups with high and low values of *b* and *c* behave differently as well, although it is not as obvious as with high values of *a* and *b*. As the estimated values of *α*_0*k*_ are much higher than the estimated values of *β*_0*k*_ and *γ*_0*k*_ and the exponent of *a*_*ik*_ is taken, a small value for *a*_*i*_ results in a overall larger deviation away from *a*_0*k*_ than a small value for *b*_*i*_ results in a deviation away from *b*_0*k*_. This explains why higher values of *b* and smaller values of *c* are less clearly visible in Fig 5, as the average mean values of these subgroups are still fairly close to the average growth curve.

#### Relation to yield

As expected, the subgroups with a high *a* have all relatively high yields, while the subgroups with a low *a* have a low yield (cf. Table 4 and its extended versions in S2 and S3 Tables). The only exception seems to be the second subgroup, where the average yield is slightly lower than the overall average. However, if we take a closer look to the fields contained in this subgroup, we observe that many fields of this group were cultivated in 2016 and 2018: these two years have a lower average yield. In comparison to the average yields of these years, the subgroup’s average yield is not so low at all. This confirms that maximum tuber growth based on three plants is indeed a good predictor for yield at field level, even though the sample size is small.

In addition, again after considering all subgroups on the Pareto front, we observe that most subgroups with a low *b* often have low yields (cf. lower half of Table 5 and its extended version in S5 Table). High values of *b*, on the other hand, do not necessarily relate to fields with high yields (with an exception of some very small subgroups) (cf. upper half of Table 5 and its extended version in S4 Table): on most fields where tuber growth is steeper than normal, the final yield is close to the average (note that we checked explicitly for yield, not if the estimated growth curve had a high maximum). No clear relation between yield and high/low values of *c* is found as well, implying that it does not matter for the final yield if halfway maximum growth is reached sooner or later (cf. Table 6 and its extended versions in S6 and S7 Tables).

#### Relation to irrigation and planting date

The subgroups did not have a clear relation between exceptional growth and irrigation (Table 7). With an exception of the first subgroup of low *a* (seven without irrigation and eight with irrigation) and the first subgroup of high *a* (ten without irrigation, fourteen with irrigation), all subgroups contain more fields without irrigation (ranging between 52% and 79%). This is similar to the ratio of fields with and without irrigation of the entire dataset (60% cannot be irrigated, the others can).

**Table 7:** Top-5 subgroups of high and low values of *a*, *b* and *c* and the frequency of irrigation and the moment when the fields were planted in the planting period. The planting period was divided into three, resulting in fields that were planted early, in the middle of the planting period, and late.

Random effect	Subgroup	Irrigation		Planting moment		
		No	Yes	Early	Middle	Late
High *a*	B_soil >1113.6 ∧ K_soil >165.5	10	14	13	3	8
	Ca_soil >196.8 ∧ Zn_soil ≤2055.6	12	7	6	3	10
	K_soil >308.1 ∧ Zn_soil ≤2082.0	15	10	11	4	10
	Fe_soil >324.0 ∧ Fe_soil ≤444.0	29	18	13	10	24
	B_soil ≤366.0 ∧ Dryness ≠ wet	18	8	5	7	14
Low *a*	Dryness ≠ average ∧ Zn_soil >6868.8 ∧ Zn_soil ≤11569.2	7	8	6	4	5
	S_soil ≤22.8 ∧ Dryness = wet ∧ Fe_soil >446.4	20	7	12	6	9
	K_soil ≤308.1 ∧ Dryness = wet ∧ Nutrient_content ≠ poor	18	7	9	6	10
	N_soil ≤138.2 ∧ Dryness = wet ∧ Nutrient_content ≠ poor	15	6	9	5	7
	Mn_soil >7648.8 ∧ Mn_soil >9234.0 ∧ K_soil ≤375.3	24	14	12	7	19
High *b*	B_soil >564.0 ∧ N_soil >174.2 ∧ K_soil ≤183.1	15	4	4	3	12
	S_soil >11.4 ∧ Dryness ≠ average ∧ K_soil ≤166.2	24	13	11	9	17
	Dryness ≠ average ∧ S_soil >10.0 ∧ K_soil ≤166.7	25	13	11	9	18
	K_soil ≤146.1 ∧ Dryness ≠ average ∧ N_soil >101.6	15	7	5	7	10
	Dryness ≠ average ∧ K_soil ≤274.7 ∧ B_soil >453.6	22	17	14	11	14
Low *b*	B_soil ≤564.0 ∧ Dryness ≠ wet	32	11	5	10	28
	Dryness ≠ wet ∧ N_soil ≤89.8	27	8	4	11	20
	Dryness ≠ wet ∧ B_soil ≤750.0	39	13	5	15	32
	Ca_soil >196.8 ∧ Zn_soil ≤2055.6	12	7	6	3	10
	Dryness ≠ wet ∧ Zn_soil ≤1290.0	10	8	1	4	13
High *c*	Fe_soil >324.0 ∧ P_soil >6.0	14	8	12	5	5
	Zn_soil >7294.8 ∧ B_soil ≤592.8	19	17	13	9	14
	K_soil ≤146.1 ∧ Ca_soil >14.7	12	6	11	4	3
	Mn_soil >1454.4 ∧ Mg_soil ≤141.5	26	10	10	11	15
	Zn_soil >3906.0 ∧ Mg_soil ≤133.2	18	6	7	7	10
Low *c*	N_soil >40.2 ∧ K_soil ≤234.0	93	53	41	34	71
	N_soil >40.2 ∧ Mg_soil >202.2	78	63	46	36	59
	Zn_soil ≤3906.0 ∧ Mg_soil >228.0	60	44	31	29	44
	Fe_soil ≤324.0 ∧ K_soil ≤216.3	59	50	34	29	46
	K_soil ≤146.1 ∧ B_soil >1405.2	20	4	6	3	15

In addition, we checked if the planting date influenced the results (Table 7). Subgroups with high values of *b* tend to slightly favour fields that were planted relatively late within the planting period. This is not surprising, as potatoes grow faster under warmer circumstances. We see a slight preference for late planting in subgroups with exceptionally low values of *c*. Given the strong correlation between *b* and *c*, this is also not surprising. Interestingly enough, we observe that fields with exceptional low values of *b* also contain slightly more fields that were planted later during the growing season. This suggests that the shape of the growth curve cannot only be defined by management and soil circumstances play an important role as well.

#### Descriptions of the subgroups

While the fourth subgroup with low *a* suggests that having too little nitrogen (N) relates to a low maximum tuber growth, none of the subgroups found with high values of *a* have N in their description (Table 4). Therefore, there seems to be only little influence of N soil content on the maximum tuber growth. N does seem to influence the steepness of the growth curve (*b*), where high values of N increase the steepness of the growth curve and small values of N flatten the growth curve (Table 5). In addition, we observe that fields need to have a minimum of N in the soil in order to advance the moment in time when halfway of the maximum growth is reached (*c*) (Table 6).

The soil K content seems to strongly influence growth: high values of potassium (K) are found in the subgroups with high values of *a*, while at the same time, low values of K are found in subgroups with low values of *a* (for an overview of the distribution of the variables, see S1 Fig). These low values of K influence other aspects of growth as well: if the steepness of the growth curve increases, the amount of K in the soil decreases. As *a* and *b* are negatively correlated, this is not surprising. At the same time, smaller values of K are also found in subgroups with lower values of *c*. Given the fact that *b* and *c* are also negatively correlated, this is not completely unexpected. However, we do observe that the average yield in these subgroups is still quite high, which indicates the relation between yield and K is non-linear and is dependent on other factors as well.

We observe that high values of calcium (Ca) combined with low values of zinc (Zn) relate to high values of *a*. Exactly the same subgroup can be found when searching for low values of *b*; as there is a strong negative correlation between *a* and *b*, it was expected that at least some subgroups would be exactly the same.

In addition, the magnesium (Mg) soil content is present in the definitions of many subgroups influencing *c*, indicating that Mg influences the moment in time when the midpoint of the maximum growth is reached: higher values of Mg are found in subgroups with lower values of *c*, while lower values of Mg are found in subgroups with higher values of *c*.

The third subgroup with high values of *a* indicates that iron (Fe) soil amount should lie within a certain range in order to facilitate a high tuber weight. This is confirmed by the second subgroup of low *a*, where low values of *a* are found when Fe soil content is too high. Fe soil content pops up as a descriptor of low values of *c* as well: as with the soil K content, low values of Fe are preferred (that lie outside of the range) in order to bring the halfway point forward in the growing season.

The Zn soil content influences growth as well. First, we observe that lower values of Zn are found in subgroups with high values of *a*, and high values of Zn are found in subgroups with a low maximum tuber weight. In addition, as expected given the correlation between *a* and *b*, low values of Zn are found in the subgroups with low values of *b*. Zn soil content is selected as a descriptor for high and low values of *c* as well: high values of Zn are found in high subgroups searching for high values of *c*, while low values of Zn relate to low values of *c*.

We find two subgroups with very high values of manganese (Mn): one has low values of *a*, the other has high values of *c*. For both subgroups, yields are low.

Boron (B) soil content is not completely consistent throughout the descriptions of the subgroups. Extremely high values of B correspond to high tuber growth, and low values of B as well. For the two other random effects, we do observe some consistency in the appearance of B: high values of B are found in subgroups with high values of *b* and with low values of *c*. The opposite effect is seen as well: low values of B are found in subgroups with respectively low and high values of *b* and *c*.

The farmer’s father has categorized all fields in poor, average, or rich fields based on his experience. Given the descriptions of subgroups that have a low *a*, a poor field seems to be a good indicator of fields where low maxima of tuber growth are reached; the data confirms the farmer’s father’s expertise.

Finally, we observe that the dryness of the field influences growth. Low values of *a* are found on fields that are not wet (three out of five subgroups suggest this), while low values of *b* are found on subgroups that are wet (four out of five). Given the strong correlation between *a* and *b*, it is not surprising that there is no overlap in fields between these subgroups.

## Discussion

### Implications for the farm

We applied the EGM framework on the dataset of the Dutch potato farm. For the remainder of this section, we shortly outline the findings of the growth curves, and how these growth curves can help to optimize farm management, and then continue with interpreting the descriptions of the found subgroups.

We started by estimating field-specific tuber growth curves and these curves describe the between-field variability in tuber growth very well (*R*^2^ of 0.92). The maximum (*a*) and the steepness of the growth curve (*b*) have a strong negative correlation. This means that in order to obtain a high maximal tuber weight, it is expected that the linear growth of that curve has to be relatively flat. A strong negative correlation was found between *b* and the moment when half of the maximum growth was produced (random effect *c*). Random effects *a* and *c* have a lower correlation. High yields were found on subgroups with high values of *a* and low values of *c*. Low yields were found in subgroups with low values of *a* and *b*.

The farm of the case study consists of many small fields, many of which are rented from other farmers for only one year: the farmer does not control what happens to the soil, and the fields are spread out over a large area. As a consequence, it takes about 45 days to plant all fields, influencing the growing period. The subgroups can be used to strategically manage the planting period to optimize overall yield: they provide guidance on farm management. The soil nutrients clearly influence the shape of the growth: subgroups with high maxima relate to subgroups with a flat linear growth period, and as a result, it takes a long time before the maximum growth is reached. In order to fully exploit the potential of these fields, these fields should be planted early. After that, the fields should be planted which have soil nutrients that lead us to expect that the midway point of growth is reached relatively late (high values of *c*). Given the correlation between *b* and *c*, these curves are often also quite flat, stretching the period of growth; as there is only a small correlation between *a* and *c*, the maximal tuber weight is not necessary as high, and therefore these fields stop growing slightly sooner. Fields that should be planted last are the fields on which a low maximum tuber weight is expected. These growth curves often have a steep linear growth, implying that maximum is reached relatively fast, while the maximum is low. This could mean that these fields can still reach their full potential even when the growing season is short.

The insights obtained with EGM can also be used for management purposes throughout the growing season. For example, water deficiency at tuber initiation can reduce the number of tubers, and during the tuber-bulking period water deficiency at mid-bulking is more harmful than deficiency during the early- and late-bulking [26]. Because EGM can help explain when these critical periods occur based on the field properties, these insights could help to improve irrigation management by ensuring that a field is irrigated based the development stage of the potato plants. In a similar fashion, fertilization management can be adjusted as well, such as applying Phosphorus (P) fertilization during tuber initiation [27].

N soil content seems to influence the steepness of the growth curve, where higher values of N are found on fields where the potatoes grow faster and do not reach a high maximum of tuber weight. The likely explanation is because of the high N soil content, leaf growth and consequently tuber growth is fast in the beginning of the season, but plants invest relatively less in the tuber growth compared to leaf growth [18]. Three additional reasons why the N soil content does not necessarily relate to high tuber growth are provided in [28]. First, the plants grown on fields with a high N content have a high N plant as well, and thus attract more insects and herbivores. Second, a high N content can alter the leaf color and third, soils with a high N stimulate weed growth as well.

In many descriptions, K is used as a descriptor influencing tuber growth. In general, higher values of K positively influence tuber growth: a large amount of K soil content relates to a higher tuber weight, whereas low values of K relate to steep growth curves that end up with a low tuber weight. This is not surprising: K is one of the main nutrients required for plant growth [29]. We do, however, find that low values of K are found on fields that have a low value of *c*, but still perform well in terms of yield (first and fourth subgroup with low *c*). This might be because plants have developed specific K-transport systems to ensure sufficient K uptake when the K soil content is low [30]. At the farm, the farmer bases the amount of K application on the soil samples, the nutrient content as estimated by the farmer’s team, the manure type that has been applied to the field on the beginning of the season, and the variety (although about 90% of all fields is cultivated with Fontane). This should result that every field has exactly the right amount of K available to meet the plant’s K demand, indicating that K availability is limited by other soil properties, possibly due to the pH-value of the soil.

In contrast to K, Mg does not have a specific transport system to ensure sufficient Mg uptake [31]. It is, however, crucial for chlorophyll synthesis and in the case of potatoes, it is a key element of potato quality [32]. The results indicate that subgroups containing a high Mg soil content reach the halfway mark of maximum growth sooner, while low Mg soil content values delay grow. This could indicate that Mg enhances growth by speeding up the growth process.

The third subgroup of high values of *a* implies a range in which the soil Fe content should lie. Fe is an important micronutrient for numerous cellular functions, but at the same time, it can cause severe problems for plants due to its insolubility and its toxicity [33]. Further, Fe is positively correlated with soil organic matter [17], which is an important measure of soil quality. In addition, Fe is negatively correlated with the soil’s pH [17]. If the pH of the soil is too low, it prevents plants from taking up nutrients, which results in reduced growth [34], but, unfortunately, there are no data of the soil’s pH available in the analyzed dataset. The range of Fe could be related to these issues: some Fe is necessary, but too much could limit potato plant growth.

The results indicate that the effect of B on growth is not always consistent, where both high and low values can result in high values of *a*. A possible explanation could be that the amount of available water influences the effect of B on potato growth: large amounts of B in soil water can reduce yield [35]. A hypothesis based on these results is that soil B content influences growth differently under dry and wet soil circumstances: it could be that dry soils require more B to enhance potato growth, while wet soils require a lower B soil content. If we take a closer look into the first subgroup, we indeed observe that the first subgroup almost only contains average and dry soils, which supports this possible explanation.

We find that many descriptions suggest that Zn and Mn in the soil should not be too high. As with Fe, high amounts of Zn and Mn are negatively correlated with the pH in the soil [17]. As mentioned, there are no data of the soil pH, but as this is such an important factor, it is likely that extreme values of soil nutrients are related to the soil pH. After 2018, the farmer has started collecting data of the soil’s pH, and the farmer mentioned that the pH of the soil at some fields is a problem at his farm. It is thus likely that these subgroups indicate that on these specific fields where Zn and Mn content is not too high, there are no problems with the pH, allowing the plants to properly take up nutrients on these fields.

If we compare the descriptions with the ranges provided by Eurofins [16] (see S1 Fig for more details), we observe that the cut-offs of N, K, and Mg soil content provided by EGM are often similar to the ranges provided by Eurofins. Interestingly, the EGM-suggested range in which values of Fe should lie is narrower than the range suggested by Eurofins. This could implicate that values of Fe that are too high or too low have a relatively large negative impact on growth, resulting in a stricter range. The cut-offs of Mn, Zn, and B soil content are considerably higher than the maximum range, which could be related to the pH of the soil.

Based on all these results, the soils of the subgroups with suboptimal growth likely have a low pH-value, resulting in problems with K and Mg plant availability and having concentrations of Mn that are toxic and therefore reducing plant growth [36]. Practical advice that follows from these results is to apply chalk in order to improve soil’s pH [37]. Currently, the farmer himself considers the pH as a problem on his fields and has started applying chalk (without any Mn) to the problematic fields to increase the soil’s pH.

### Using EGM in other cases

In cases where longitudinal growth data have been collected combined with some possible descriptors of the growth, EGM can be applied. On the farm of the case study, many small fields are cultivated, observing mainly variability between these fields. In other cases, variability between different zones could be monitored, or maybe even between different plants. When applying EGM, it is necessary to determine the scale of variability and the unit on which growth is monitored. In addition, it is important that these fields are randomly sampled, such that the total area under consideration is represented and that good, mediocre, and bad fields are all represented.

For our case study, we use an s-shaped curve to describe tuber growth. In other cases, different shapes might be more appropriate. For example, haulm weight first steeply increases and after the maximum has been reached, starts decreasing due to leaf senescence [38], making it more appropriate to use a quadratic curve without intercept [12]. The sample size required such that EGM can be applied is a trade-off between the shape of the growth curve, the number of considered fields (or zones), and the number of repeated measurements. In cases where the parameters of the growth curve are linear in its parameters, the absolute minimum is three [39]. In cases where the growth curve is non-linear in its parameters, the minimum is higher [40].

### Methodological considerations

EGM is able to find many relationships between exceptional growth and soil parameters. While these relationships are interesting and can result in new hypotheses, EGM is not a causal framework and is sensitive to confounding [41]. Within the case study, we evaluated whether irrigation, planting date and the year in which a field was cultivated could possibly influence the results, because we hypothesized that these factors were the most important ones that could possibly influence growth as well. As manure was applied before the soil samples were taken (and should therefore be visible in the soil analysis), and fertilizer is supplied based on the soil sample results, the impact on growth is assumed to be relatively small. None of the subgroups showed clear evidence that solely these potential confounders influenced exceptionality in growth. This indicates that the EGM-discovered relationships contribute to exceptional growth and can be useful to estimate crop response to soil nutrient supply.

The descriptions of the subgroups can be influenced by collinearity. It is unknown to what extent collinearity influences exceptional model mining. but in case of least square regression, variables are assumed to be highly collinear if |*r*|>0.7 [42]. For the case study, only Ca en K are highly collinear and we thus expect that the overall influence of collinearity on the results is minimal, with an exception of the second and third subgroup of high values of *a* (Ca_soil >196.8 kg ha^−1^ ∧ Zn_soil ≤2055.6 gr ha^−1^ and K_soil >308.1 kg ha^−1^ ∧ Zn_soil ≤2082.0 gr ha^−1^): 14 out of 19 fields of the subgroup using Ca in its description are also present in the subgroup using K in its description. As K is present in so many of the descriptions of all random effects, we expect that K is more important than the effect of Ca. More research needs to be performed in order to investigate the impact of collinearity on the results.

In addition, the descriptions are influenced by the depth *d* of the beam search algorithm. The larger *d* is, the more descriptors are added to the description. This often leads to some kind of redundancy in the result set, where the first part of the description stays the same, and the final descriptor changes, ultimately referring to the same subgroup. On the other hand, if we set *d* too small, the subgroups are not exceptional enough, resulting in subgroups with a mean close to zero and a large standard deviation. By ensuring that we take *d* in such a way that most subgroups contain more than 20 fields, we observe that the descriptions are still different from each other, and that the found behavior is still exceptional.

In the case study, at each sampling round, only three plants were pulled out of the soil. While this seems little to properly represent the entire field’s tuber growth, we do observe that subgroups found with high and low tuber growth maxima have the expected relationship with yield. As already mentioned in [12], a possible explanation is that a total area of about 5 m^−2^ is sampled, because at each field about 5 to 7 sampling rounds are performed. Similar results were found in [43], but nonetheless, more research should be performed in order to investigate how many plants need to be sampled to correctly represent the field’s relation to yield.

EGM can be applied to investigate how various growth parameters interact, and how these growth parameters are affected by external factors. Here, we investigated the effect of soil parameters on these growth parameters at one farm, but EGM can also be applied when the interest lies in analyzing growth of multiple farms and/or analyzing all kinds of other factors that could influence growth. Because of the use of mixed models, our methodology is robust against missing data and able to handle non-equidistant spacing [39].

In order to better understand the performance of EGM, it would be recommended to apply EGM on more datasets. Unfortunately, to our knowledge, such datasets within the agronomic field are not publicly available; through a relatively lightweight process, every farmer can collect such data on their own farm, but no farmer benefits from releasing this data to the public (and such a release risks leaking information that gives the farmer a competitive advantage).

## Conclusions

The first objective of this paper was to introduce Exceptional Growth Mining, a new local pattern mining method to identify various reasons for unusual plant growth, which we applied on a case study as the second objective. EGM consists of estimating growth curves, after which we investigate the correlation between the estimated growth parameters and finally, we apply Exceptional Model Mining (EMM) on these growth curves using a growth curve-specific quality measure. Subgroups are interesting only if they satisfy two properties. On the one hand, they must be *interpretable*: we must be able to define them in terms of a few constraints on selected variables (cf. the soil conditions defining a subgroup in Fig 1). On the other hand, they must display *exceptional growth behavior*: the growth curves belonging to the subgroup must deviate from growth curves observed across all fields (cf. the highlighted growth curves in Fig 1). In this paper, we explored three distinct kinds of exceptional growth in s-shaped curves: exceptionally high/low final yield, exceptionally high/low steepness of the linear part of the curve, and reaching the midpoint of growth exceptionally early/late. Exploring this variety in forms of growth behavior exceptionality, and capturing these behaviors in subgroups whose soil content definitions we can inspect, provides an analysis of the on-farm collected growth data in a manner that does justice to its inherent heterogeneity.

Applying EGM on the data of the case study led to the following results. The maximum and steepness of the field-specific tuber growth curves have a high negative correlation, just as the steepness and the moment when the halfway point of maximum growth was reached. The relation is less pronounced between the maximum and the moment when halfway maximum growth was reached. The found exceptional growth subgroups suggest that high yields correspond to high maxima and reaching halfway maximum growth relatively soon, while low yields were found in subgroups with low maximum growth and steep linear growth.

We found that the amount of K influences growth, where high values of K are seen in subgroups where high maxima were reached, while low values of K correspond to low maxima and steep growth curves. Low values of K are also seen when halfway of maximum growth is reached early in the season. In addition, we found that Mg relates to the timing of the halfway point of maximum growth: high values of Mg expedite this moment, while low Mg values delay this moment. This could indicate that Mg speeds up the growing process for potato tubers.

Furthermore, high values of Zn, Mn, and Fe were found in descriptions of subgroups with low tuber weight. As high values of these nutrients relate to low pH values, such a high values could suggest that nutrient uptake is limited on these fields due to the pH value of the soil.

Both extremely high and low values of B were found on fields with high tuber weight, which could be due to its interaction with water. High values of B were beneficial to tuber growth on dry soils, while low values are preferred on wet soils.

This work focused on identifying soil characteristics that influence growth, independently of weather circumstances. Future work should focus on how management could increase or decrease the effect of soil circumstances on growth, reaching beyond the advice already provided throughout the discussion. In addition, different soil conditions might be beneficial under different weather circumstances. The interaction between (extreme) weather circumstances and specific soil properties should be researched further.

## Supporting information

S1 Fig Histograms of the distributions of all descriptors in the dataset. On each field, soil samples were taken. These soil samples are evaluated using the Eurofins protocol, and provide us the amount of the following macro- and micronutrients: N, P, K, Ca, Mg, S, Si, Fe, Zn, Mn, and B. In these histograms, two lines are present as well. The left line represents the lower limit of the advise of Eurofins, and the right line represents the maximum of the range. In addition, some categorical variables are provided. The nutrient content of the field is determined by the farmer’s team, who classifies fields as poor, average or rich. In addition, the field is classified as dry, average or wet by the farmer himself. Potato is a rotation crop; only once per four years, potatoes can be grown on the same field. The crop cultivated before potatoes were grown on the field is the previously cultivated crop. In the “others” category all kinds of crops are captured. Usually, only one or two times, a field is cultivated with that crop. Crops in this category are for example conifers, salsify, or peas. Finally, some fields suffer from nematodes, which can have a negative effect on potato yield. A: N in soil. B: P in soil. C: K in soil. D: Ca in soil. E: Mg in soil. F: Si in soil. G: S in soil. H: Fe in soil. I: Zn in soil. J: Mn in soil. K: B in soil. L: Tuber weight. M: Nutrient content. N: Contains nematodes? O: Year. P: Dryness. Q: Previously cultivated crop.
(ZIP)

S1 Appendix SAS code employed to estimate the growth curves. (PDF)

S1 Table Spearman’s rank correlation within the dataset between all pairs of continuous descriptors. (PDF)

S2 TableExtension of the upper half of Table 4 by listing all subgroups found on the Pareto front when running Exceptional Growth Mining to search for high values of *a*, with quality measure $\phi_{{\text{GC}}_{h}}^{u}$.(PDF)

S3 TableExtension of the lower half of Table 4 by listing all subgroups found on the Pareto front when running Exceptional Growth Mining to search for low tuber growth, with quality measure $\phi_{{\text{GC}}_{l}}^{u}$.(PDF)

S4 TableExtension of the upper half of Table 5 by listing all subgroups found on the Pareto front when running Exceptional Growth Mining to search for low tuber growth, with quality measure $\phi_{{\text{GC}}_{h}}^{u}$.(PDF)

S5 TableExtension of the lower half of Table 5 by listing all subgroups found on the Pareto front when running Exceptional Growth Mining to search for low tuber growth, with quality measure $\phi_{{\text{GC}}_{l}}^{u}$.(PDF)

S6 TableExtension of the upper half of Table 6 by listing all subgroups found on the Pareto front when running Exceptional Growth Mining to search for low tuber growth, with quality measure $\phi_{{\text{GC}}_{h}}^{u}$.(PDF)

S7 TableExtension of the upper half of Table 6 by listing all subgroups found on the Pareto front when running Exceptional Growth Mining to search for low tuber growth, with quality measure $\phi_{{\text{GC}}_{l}}^{u}$.(PDF)
