# Supplementary figures and images for: Introducing exceptional growth mining—Analyzing the impact of soil characteristics on on-farm crop growth and yield variability
Source: PLoS One. 2024 Jan 29;19(1):e0296684. doi: 10.1371/journal.pone.0296684 (PMC10824435; doi:10.1371/journal.pone.0296684)

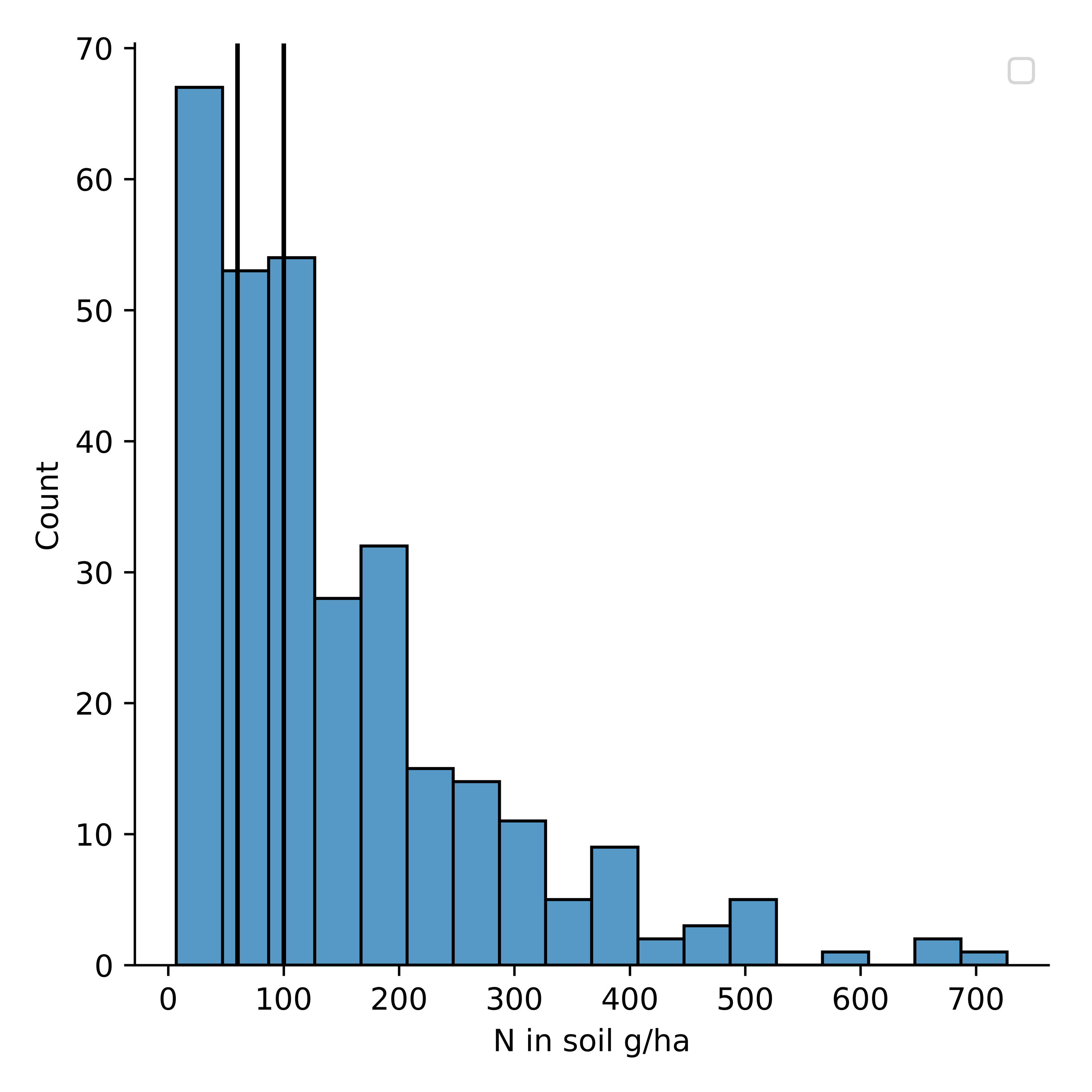

Supplement: S1 Fig — On each field, soil samples were taken. These soil samples are evaluated using the Eurofins protocol, and provide us the amount of the following macro- and micronutrients: N, P, K, Ca, Mg, S, Si, Fe, Zn, Mn, and B. In these histograms, two lines are present as well. The left line represents the lower limit of the advise of Eurofins, and the right line represents the maximum of the range. In addition, some categorical variables are provided. The nutrient content of the field is determined by the farmer’s team, who classifies fields as poor, average or rich. In addition, the field is classified as dry, average or wet by the farmer himself. Potato is a rotation crop; only once per four years, potatoes can be grown on the same field. The crop cultivated before potatoes were grown on the field is the previously cultivated crop. In the “others” category all kinds of crops are captured. Usually, only one or two times, a field is cultivated with that crop. Crops in this category are for example conifers, salsify, or peas. Finally, some fields suffer from nematodes, which can have a negative effect on potato yield. A: N in soil. B: P in soil. C: K in soil. D: Ca in soil. E: Mg in soil. F: Si in soil. G: S in soil. H: Fe in soil. I: Zn in soil. J: Mn in soil. K: B in soil. L: Tuber weight. M: Nutrient content. N: Contains nematodes? O: Year. P: Dryness. Q: Previously cultivated crop. (ZIP) [file pone.0296684.s001.zip › S1A_Fig.tif]

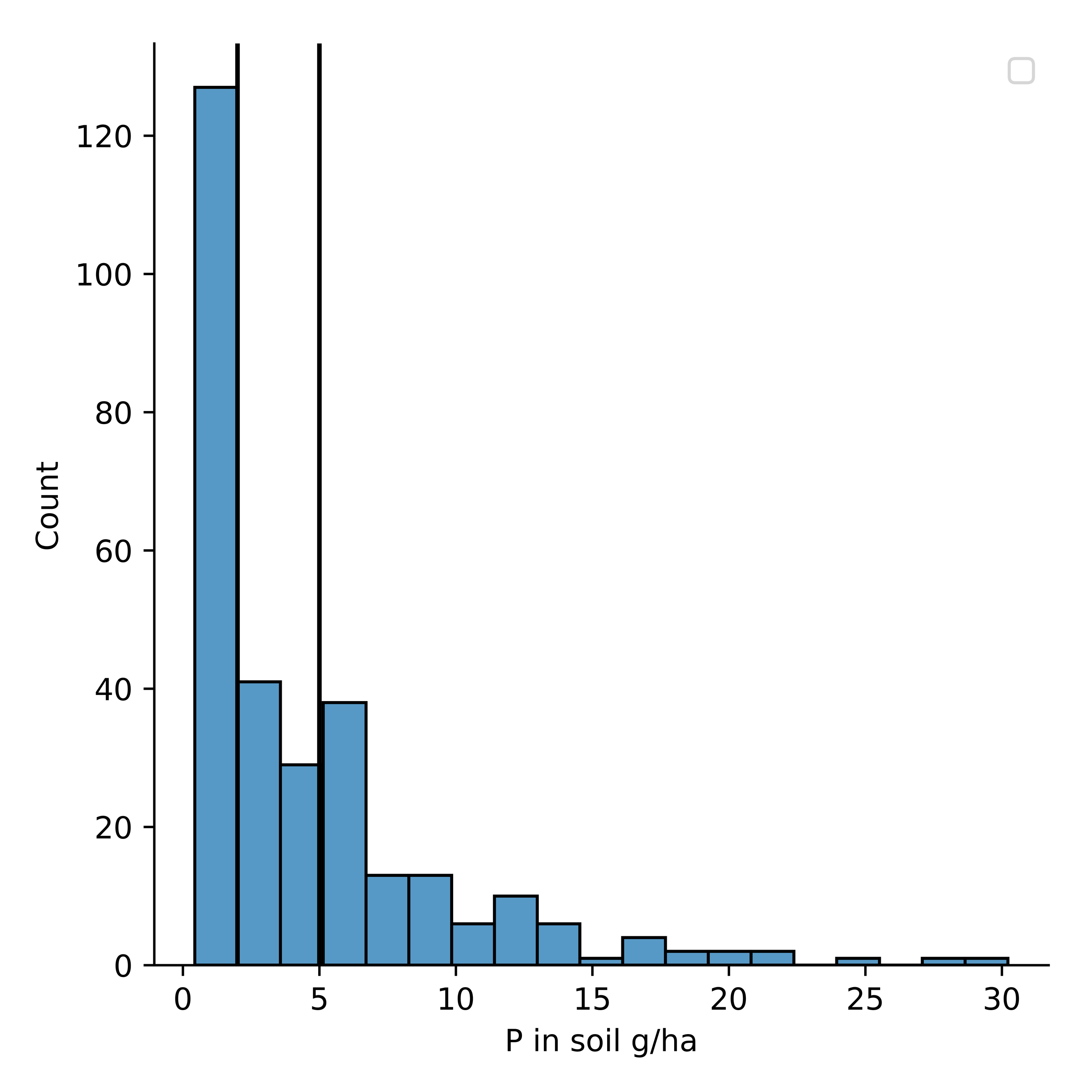

Supplement: S1 Fig — On each field, soil samples were taken. These soil samples are evaluated using the Eurofins protocol, and provide us the amount of the following macro- and micronutrients: N, P, K, Ca, Mg, S, Si, Fe, Zn, Mn, and B. In these histograms, two lines are present as well. The left line represents the lower limit of the advise of Eurofins, and the right line represents the maximum of the range. In addition, some categorical variables are provided. The nutrient content of the field is determined by the farmer’s team, who classifies fields as poor, average or rich. In addition, the field is classified as dry, average or wet by the farmer himself. Potato is a rotation crop; only once per four years, potatoes can be grown on the same field. The crop cultivated before potatoes were grown on the field is the previously cultivated crop. In the “others” category all kinds of crops are captured. Usually, only one or two times, a field is cultivated with that crop. Crops in this category are for example conifers, salsify, or peas. Finally, some fields suffer from nematodes, which can have a negative effect on potato yield. A: N in soil. B: P in soil. C: K in soil. D: Ca in soil. E: Mg in soil. F: Si in soil. G: S in soil. H: Fe in soil. I: Zn in soil. J: Mn in soil. K: B in soil. L: Tuber weight. M: Nutrient content. N: Contains nematodes? O: Year. P: Dryness. Q: Previously cultivated crop. (ZIP) [file pone.0296684.s001.zip › S1B_Fig.tif]

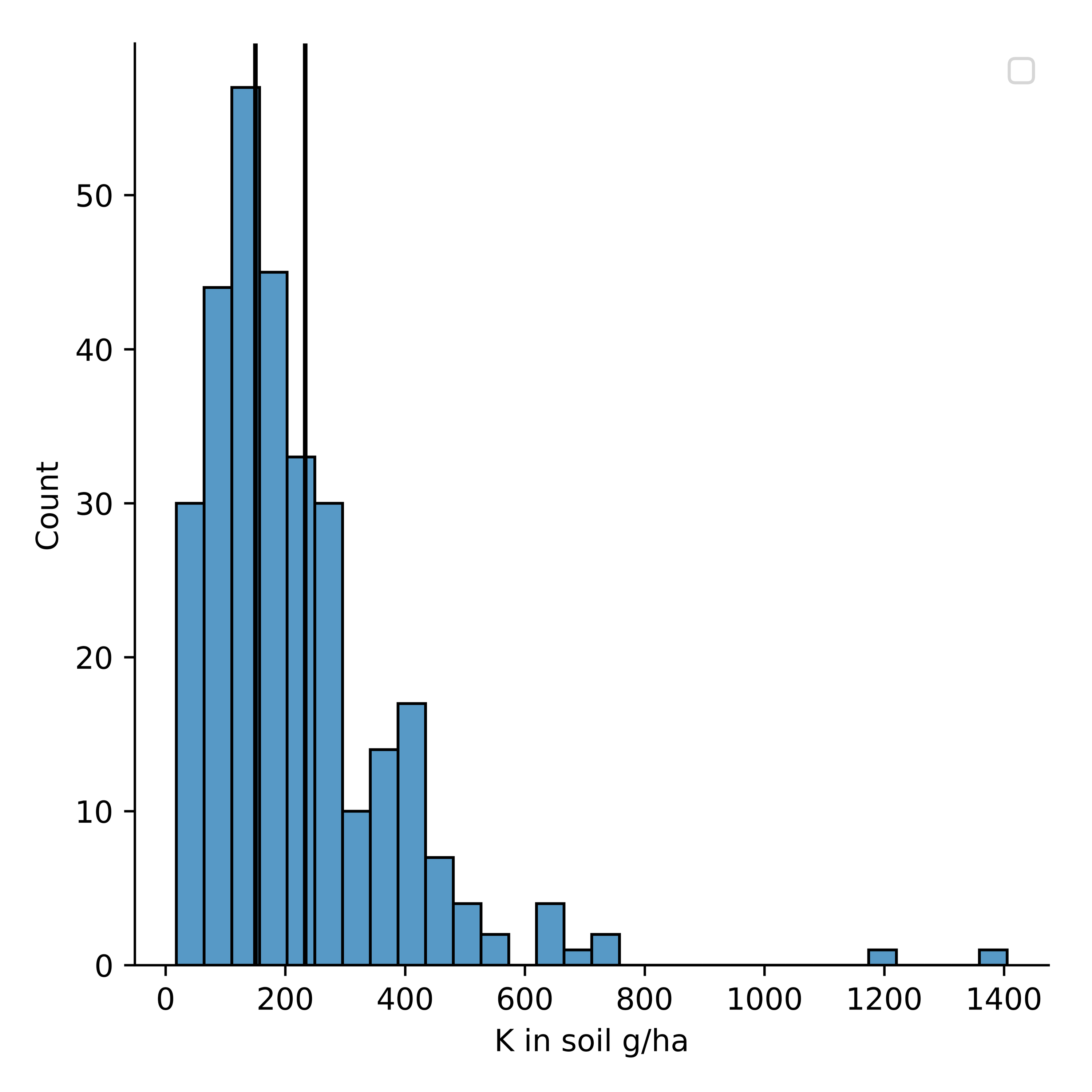

Supplement: S1 Fig — On each field, soil samples were taken. These soil samples are evaluated using the Eurofins protocol, and provide us the amount of the following macro- and micronutrients: N, P, K, Ca, Mg, S, Si, Fe, Zn, Mn, and B. In these histograms, two lines are present as well. The left line represents the lower limit of the advise of Eurofins, and the right line represents the maximum of the range. In addition, some categorical variables are provided. The nutrient content of the field is determined by the farmer’s team, who classifies fields as poor, average or rich. In addition, the field is classified as dry, average or wet by the farmer himself. Potato is a rotation crop; only once per four years, potatoes can be grown on the same field. The crop cultivated before potatoes were grown on the field is the previously cultivated crop. In the “others” category all kinds of crops are captured. Usually, only one or two times, a field is cultivated with that crop. Crops in this category are for example conifers, salsify, or peas. Finally, some fields suffer from nematodes, which can have a negative effect on potato yield. A: N in soil. B: P in soil. C: K in soil. D: Ca in soil. E: Mg in soil. F: Si in soil. G: S in soil. H: Fe in soil. I: Zn in soil. J: Mn in soil. K: B in soil. L: Tuber weight. M: Nutrient content. N: Contains nematodes? O: Year. P: Dryness. Q: Previously cultivated crop. (ZIP) [file pone.0296684.s001.zip › S1C_Fig.tif]

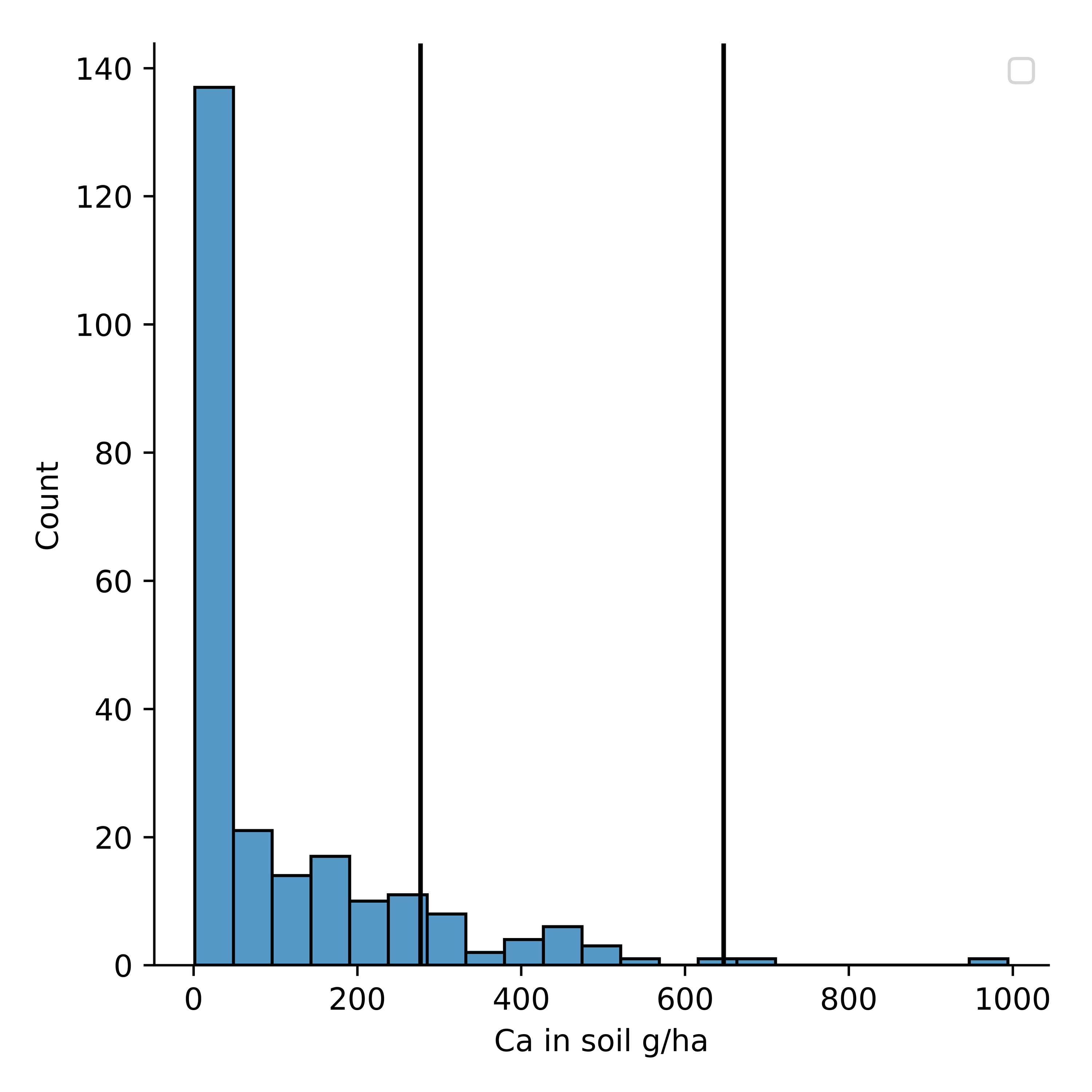

Supplement: S1 Fig — On each field, soil samples were taken. These soil samples are evaluated using the Eurofins protocol, and provide us the amount of the following macro- and micronutrients: N, P, K, Ca, Mg, S, Si, Fe, Zn, Mn, and B. In these histograms, two lines are present as well. The left line represents the lower limit of the advise of Eurofins, and the right line represents the maximum of the range. In addition, some categorical variables are provided. The nutrient content of the field is determined by the farmer’s team, who classifies fields as poor, average or rich. In addition, the field is classified as dry, average or wet by the farmer himself. Potato is a rotation crop; only once per four years, potatoes can be grown on the same field. The crop cultivated before potatoes were grown on the field is the previously cultivated crop. In the “others” category all kinds of crops are captured. Usually, only one or two times, a field is cultivated with that crop. Crops in this category are for example conifers, salsify, or peas. Finally, some fields suffer from nematodes, which can have a negative effect on potato yield. A: N in soil. B: P in soil. C: K in soil. D: Ca in soil. E: Mg in soil. F: Si in soil. G: S in soil. H: Fe in soil. I: Zn in soil. J: Mn in soil. K: B in soil. L: Tuber weight. M: Nutrient content. N: Contains nematodes? O: Year. P: Dryness. Q: Previously cultivated crop. (ZIP) [file pone.0296684.s001.zip › S1D_Fig.tif]

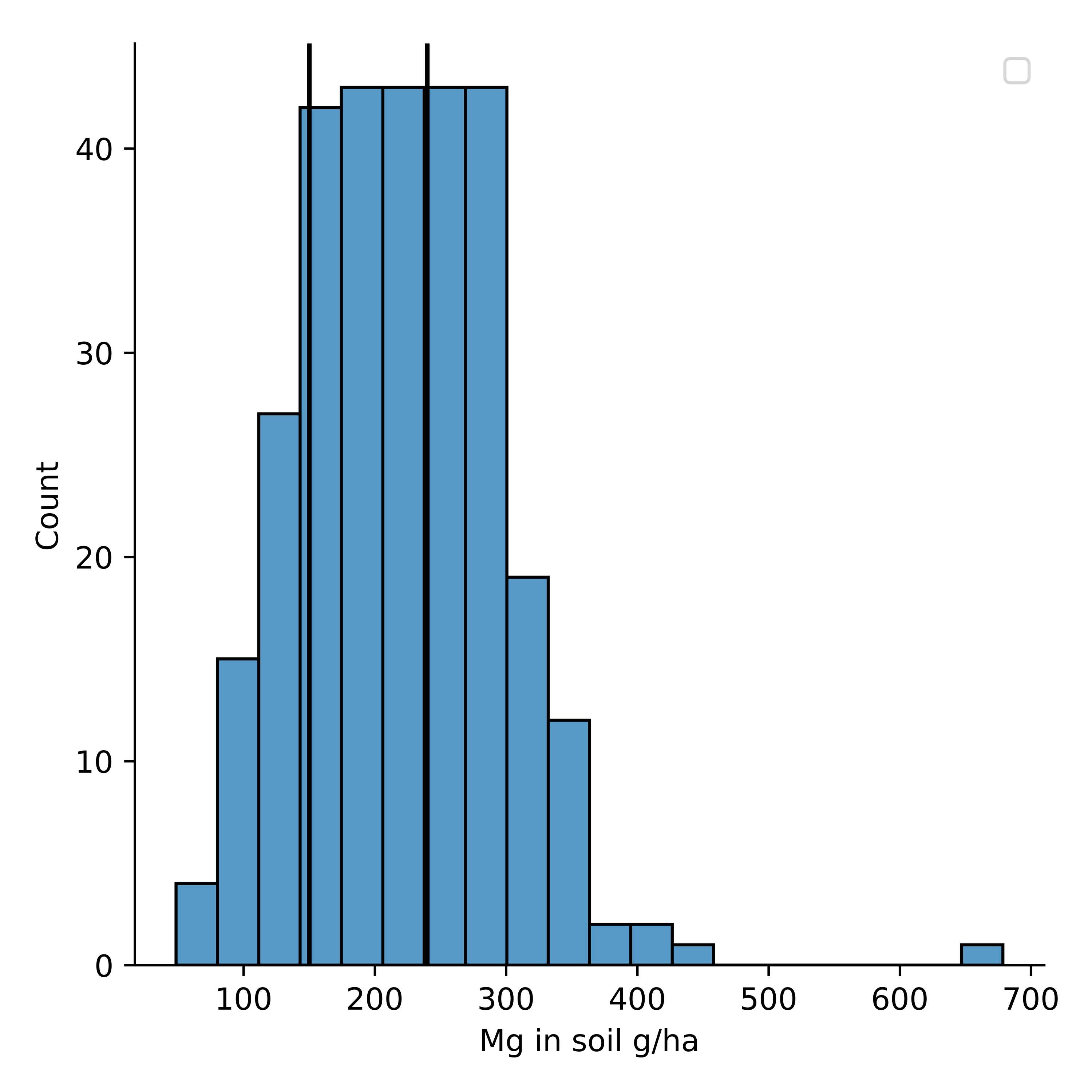

Supplement: S1 Fig — On each field, soil samples were taken. These soil samples are evaluated using the Eurofins protocol, and provide us the amount of the following macro- and micronutrients: N, P, K, Ca, Mg, S, Si, Fe, Zn, Mn, and B. In these histograms, two lines are present as well. The left line represents the lower limit of the advise of Eurofins, and the right line represents the maximum of the range. In addition, some categorical variables are provided. The nutrient content of the field is determined by the farmer’s team, who classifies fields as poor, average or rich. In addition, the field is classified as dry, average or wet by the farmer himself. Potato is a rotation crop; only once per four years, potatoes can be grown on the same field. The crop cultivated before potatoes were grown on the field is the previously cultivated crop. In the “others” category all kinds of crops are captured. Usually, only one or two times, a field is cultivated with that crop. Crops in this category are for example conifers, salsify, or peas. Finally, some fields suffer from nematodes, which can have a negative effect on potato yield. A: N in soil. B: P in soil. C: K in soil. D: Ca in soil. E: Mg in soil. F: Si in soil. G: S in soil. H: Fe in soil. I: Zn in soil. J: Mn in soil. K: B in soil. L: Tuber weight. M: Nutrient content. N: Contains nematodes? O: Year. P: Dryness. Q: Previously cultivated crop. (ZIP) [file pone.0296684.s001.zip › S1E_Fig.tif]

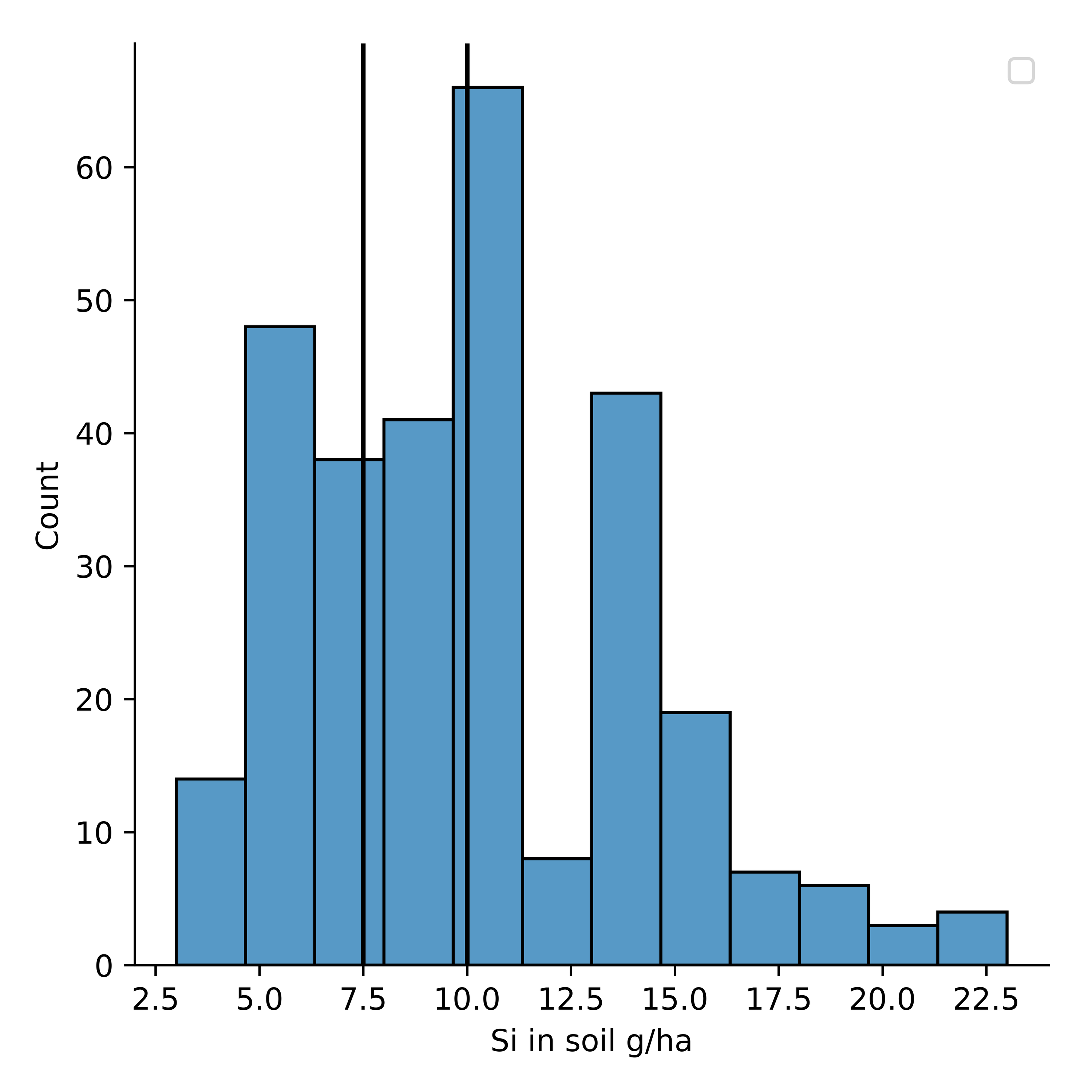

Supplement: S1 Fig — On each field, soil samples were taken. These soil samples are evaluated using the Eurofins protocol, and provide us the amount of the following macro- and micronutrients: N, P, K, Ca, Mg, S, Si, Fe, Zn, Mn, and B. In these histograms, two lines are present as well. The left line represents the lower limit of the advise of Eurofins, and the right line represents the maximum of the range. In addition, some categorical variables are provided. The nutrient content of the field is determined by the farmer’s team, who classifies fields as poor, average or rich. In addition, the field is classified as dry, average or wet by the farmer himself. Potato is a rotation crop; only once per four years, potatoes can be grown on the same field. The crop cultivated before potatoes were grown on the field is the previously cultivated crop. In the “others” category all kinds of crops are captured. Usually, only one or two times, a field is cultivated with that crop. Crops in this category are for example conifers, salsify, or peas. Finally, some fields suffer from nematodes, which can have a negative effect on potato yield. A: N in soil. B: P in soil. C: K in soil. D: Ca in soil. E: Mg in soil. F: Si in soil. G: S in soil. H: Fe in soil. I: Zn in soil. J: Mn in soil. K: B in soil. L: Tuber weight. M: Nutrient content. N: Contains nematodes? O: Year. P: Dryness. Q: Previously cultivated crop. (ZIP) [file pone.0296684.s001.zip › S1F_Fig.tif]

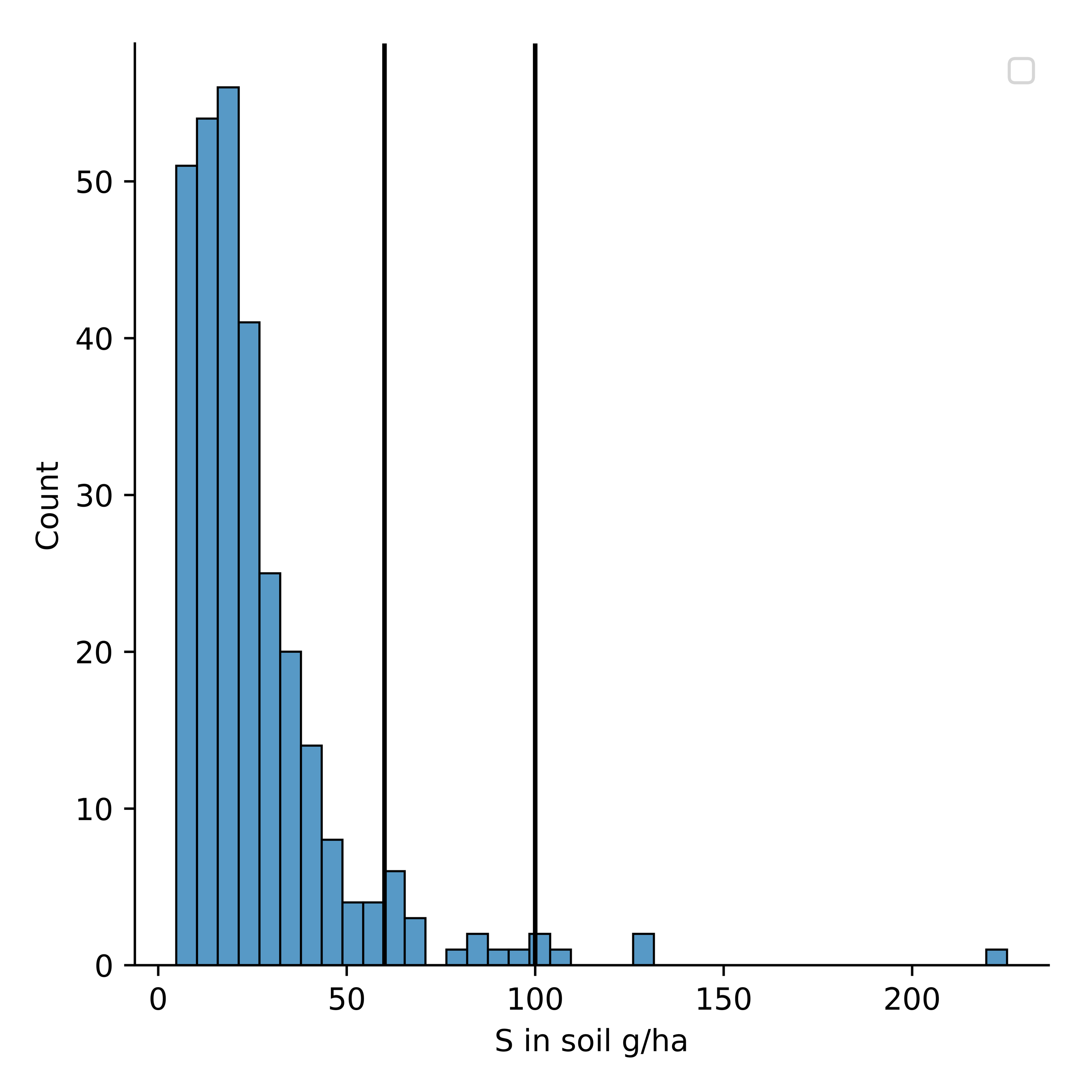

Supplement: S1 Fig — On each field, soil samples were taken. These soil samples are evaluated using the Eurofins protocol, and provide us the amount of the following macro- and micronutrients: N, P, K, Ca, Mg, S, Si, Fe, Zn, Mn, and B. In these histograms, two lines are present as well. The left line represents the lower limit of the advise of Eurofins, and the right line represents the maximum of the range. In addition, some categorical variables are provided. The nutrient content of the field is determined by the farmer’s team, who classifies fields as poor, average or rich. In addition, the field is classified as dry, average or wet by the farmer himself. Potato is a rotation crop; only once per four years, potatoes can be grown on the same field. The crop cultivated before potatoes were grown on the field is the previously cultivated crop. In the “others” category all kinds of crops are captured. Usually, only one or two times, a field is cultivated with that crop. Crops in this category are for example conifers, salsify, or peas. Finally, some fields suffer from nematodes, which can have a negative effect on potato yield. A: N in soil. B: P in soil. C: K in soil. D: Ca in soil. E: Mg in soil. F: Si in soil. G: S in soil. H: Fe in soil. I: Zn in soil. J: Mn in soil. K: B in soil. L: Tuber weight. M: Nutrient content. N: Contains nematodes? O: Year. P: Dryness. Q: Previously cultivated crop. (ZIP) [file pone.0296684.s001.zip › S1G_Fig.tif]

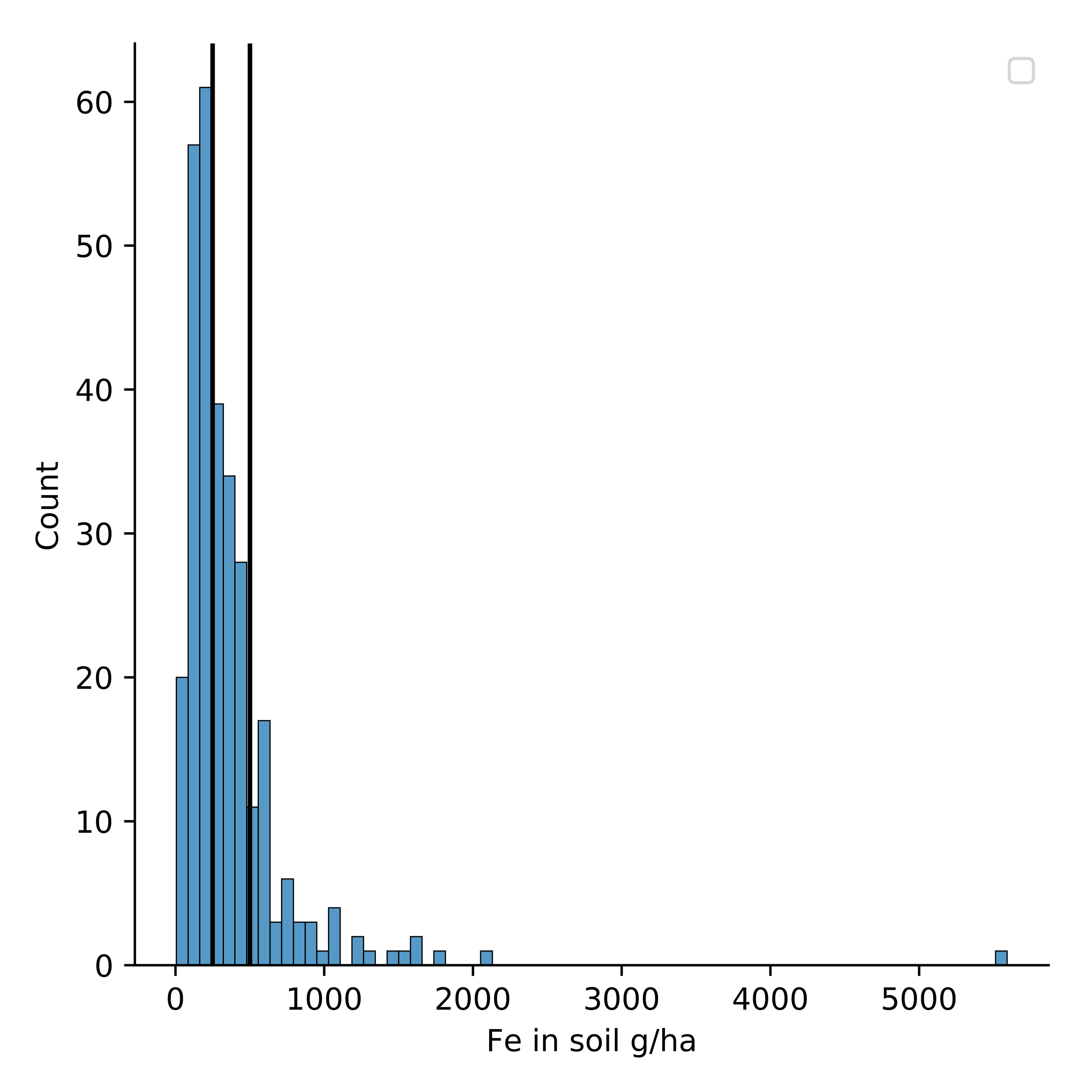

Supplement: S1 Fig — On each field, soil samples were taken. These soil samples are evaluated using the Eurofins protocol, and provide us the amount of the following macro- and micronutrients: N, P, K, Ca, Mg, S, Si, Fe, Zn, Mn, and B. In these histograms, two lines are present as well. The left line represents the lower limit of the advise of Eurofins, and the right line represents the maximum of the range. In addition, some categorical variables are provided. The nutrient content of the field is determined by the farmer’s team, who classifies fields as poor, average or rich. In addition, the field is classified as dry, average or wet by the farmer himself. Potato is a rotation crop; only once per four years, potatoes can be grown on the same field. The crop cultivated before potatoes were grown on the field is the previously cultivated crop. In the “others” category all kinds of crops are captured. Usually, only one or two times, a field is cultivated with that crop. Crops in this category are for example conifers, salsify, or peas. Finally, some fields suffer from nematodes, which can have a negative effect on potato yield. A: N in soil. B: P in soil. C: K in soil. D: Ca in soil. E: Mg in soil. F: Si in soil. G: S in soil. H: Fe in soil. I: Zn in soil. J: Mn in soil. K: B in soil. L: Tuber weight. M: Nutrient content. N: Contains nematodes? O: Year. P: Dryness. Q: Previously cultivated crop. (ZIP) [file pone.0296684.s001.zip › S1H_Fig.tif]

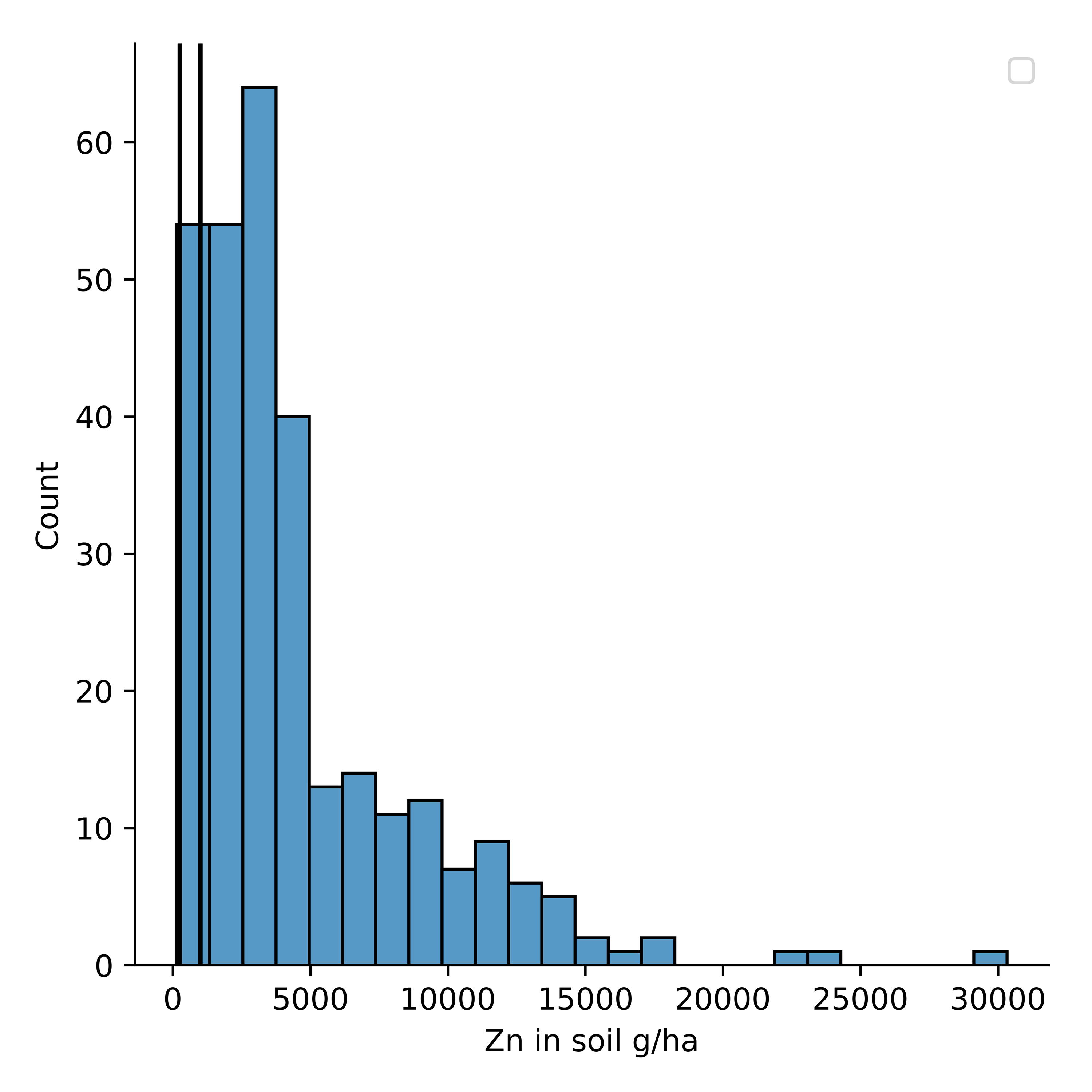

Supplement: S1 Fig — On each field, soil samples were taken. These soil samples are evaluated using the Eurofins protocol, and provide us the amount of the following macro- and micronutrients: N, P, K, Ca, Mg, S, Si, Fe, Zn, Mn, and B. In these histograms, two lines are present as well. The left line represents the lower limit of the advise of Eurofins, and the right line represents the maximum of the range. In addition, some categorical variables are provided. The nutrient content of the field is determined by the farmer’s team, who classifies fields as poor, average or rich. In addition, the field is classified as dry, average or wet by the farmer himself. Potato is a rotation crop; only once per four years, potatoes can be grown on the same field. The crop cultivated before potatoes were grown on the field is the previously cultivated crop. In the “others” category all kinds of crops are captured. Usually, only one or two times, a field is cultivated with that crop. Crops in this category are for example conifers, salsify, or peas. Finally, some fields suffer from nematodes, which can have a negative effect on potato yield. A: N in soil. B: P in soil. C: K in soil. D: Ca in soil. E: Mg in soil. F: Si in soil. G: S in soil. H: Fe in soil. I: Zn in soil. J: Mn in soil. K: B in soil. L: Tuber weight. M: Nutrient content. N: Contains nematodes? O: Year. P: Dryness. Q: Previously cultivated crop. (ZIP) [file pone.0296684.s001.zip › S1I_Fig.tif]

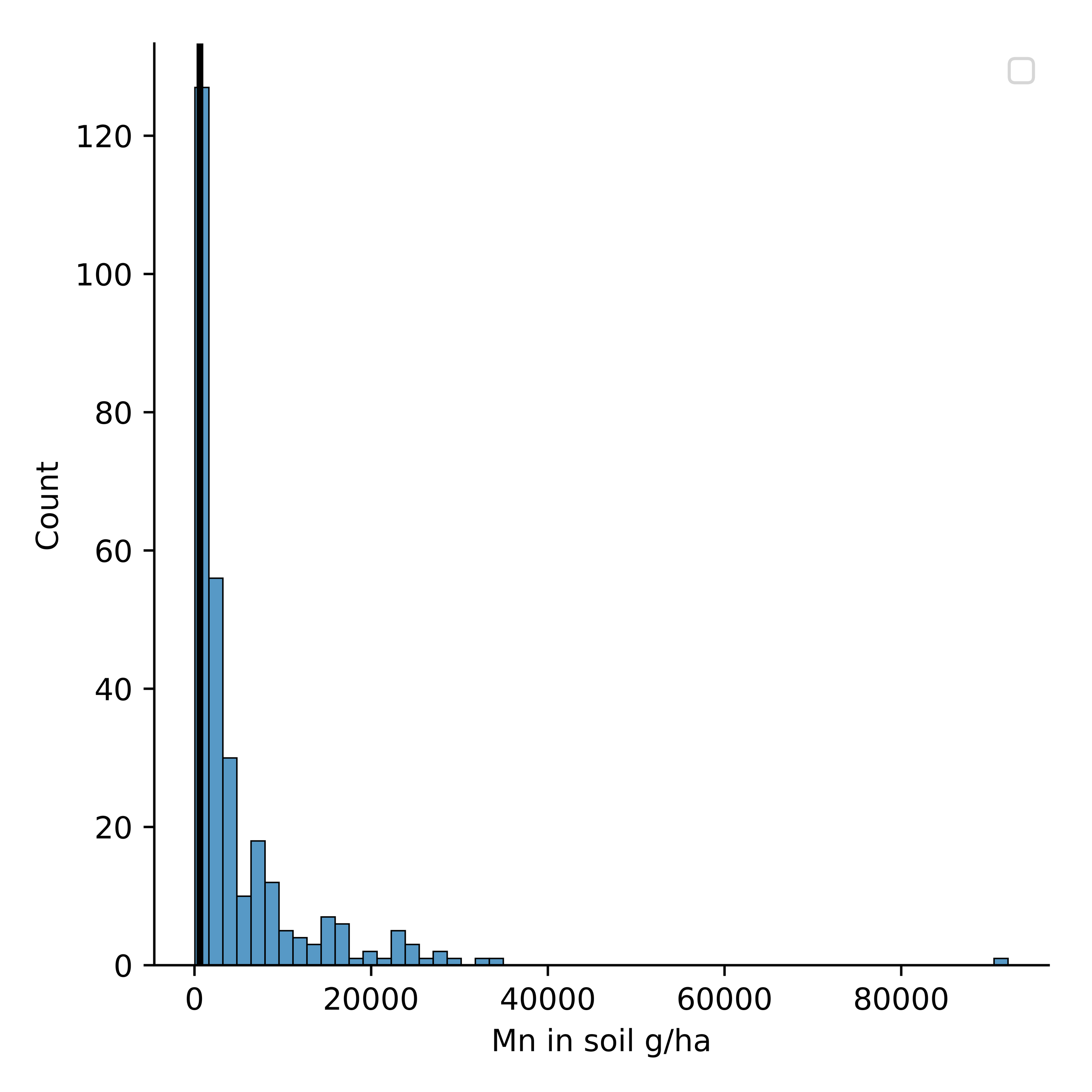

Supplement: S1 Fig — On each field, soil samples were taken. These soil samples are evaluated using the Eurofins protocol, and provide us the amount of the following macro- and micronutrients: N, P, K, Ca, Mg, S, Si, Fe, Zn, Mn, and B. In these histograms, two lines are present as well. The left line represents the lower limit of the advise of Eurofins, and the right line represents the maximum of the range. In addition, some categorical variables are provided. The nutrient content of the field is determined by the farmer’s team, who classifies fields as poor, average or rich. In addition, the field is classified as dry, average or wet by the farmer himself. Potato is a rotation crop; only once per four years, potatoes can be grown on the same field. The crop cultivated before potatoes were grown on the field is the previously cultivated crop. In the “others” category all kinds of crops are captured. Usually, only one or two times, a field is cultivated with that crop. Crops in this category are for example conifers, salsify, or peas. Finally, some fields suffer from nematodes, which can have a negative effect on potato yield. A: N in soil. B: P in soil. C: K in soil. D: Ca in soil. E: Mg in soil. F: Si in soil. G: S in soil. H: Fe in soil. I: Zn in soil. J: Mn in soil. K: B in soil. L: Tuber weight. M: Nutrient content. N: Contains nematodes? O: Year. P: Dryness. Q: Previously cultivated crop. (ZIP) [file pone.0296684.s001.zip › S1J_Fig.tif]

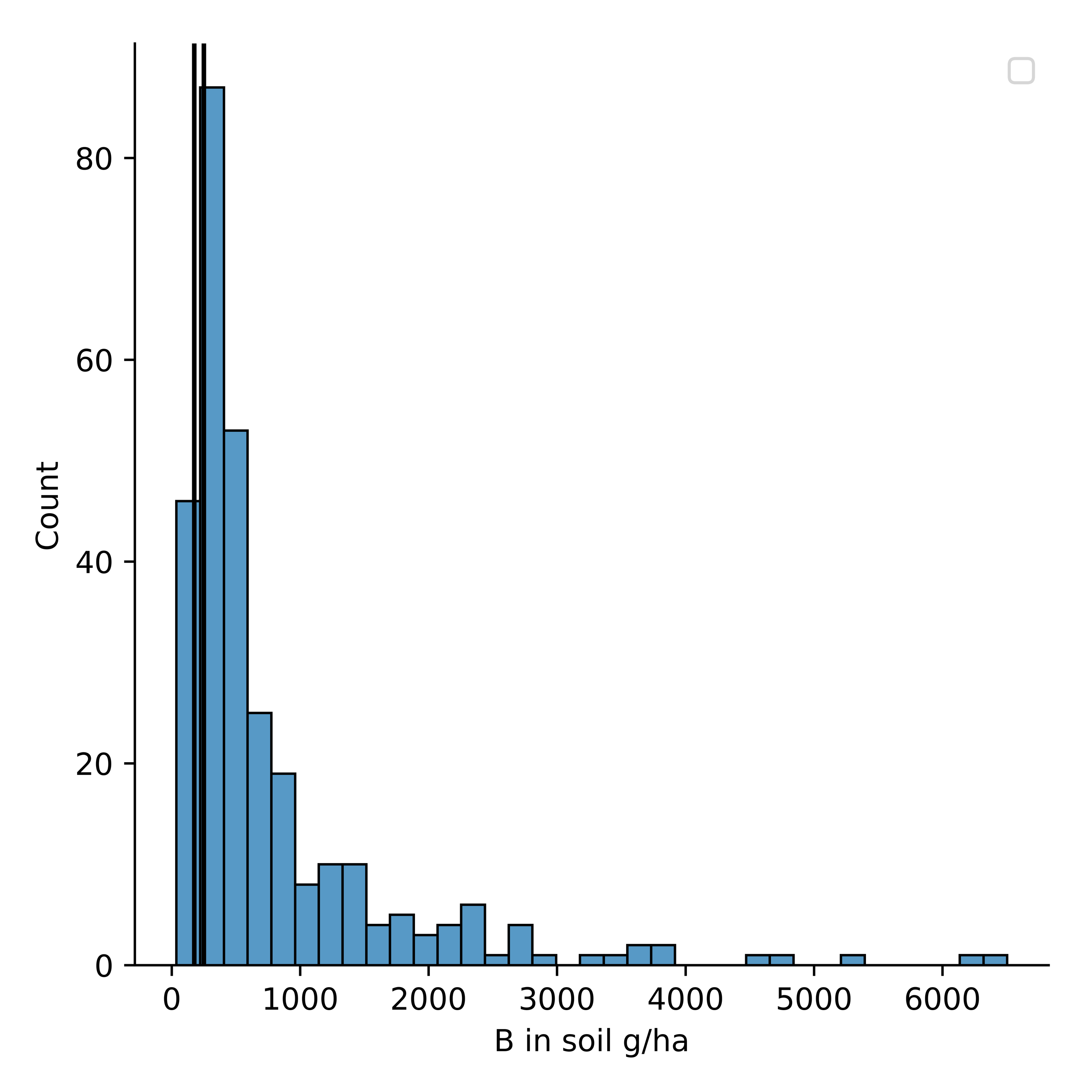

Supplement: S1 Fig — On each field, soil samples were taken. These soil samples are evaluated using the Eurofins protocol, and provide us the amount of the following macro- and micronutrients: N, P, K, Ca, Mg, S, Si, Fe, Zn, Mn, and B. In these histograms, two lines are present as well. The left line represents the lower limit of the advise of Eurofins, and the right line represents the maximum of the range. In addition, some categorical variables are provided. The nutrient content of the field is determined by the farmer’s team, who classifies fields as poor, average or rich. In addition, the field is classified as dry, average or wet by the farmer himself. Potato is a rotation crop; only once per four years, potatoes can be grown on the same field. The crop cultivated before potatoes were grown on the field is the previously cultivated crop. In the “others” category all kinds of crops are captured. Usually, only one or two times, a field is cultivated with that crop. Crops in this category are for example conifers, salsify, or peas. Finally, some fields suffer from nematodes, which can have a negative effect on potato yield. A: N in soil. B: P in soil. C: K in soil. D: Ca in soil. E: Mg in soil. F: Si in soil. G: S in soil. H: Fe in soil. I: Zn in soil. J: Mn in soil. K: B in soil. L: Tuber weight. M: Nutrient content. N: Contains nematodes? O: Year. P: Dryness. Q: Previously cultivated crop. (ZIP) [file pone.0296684.s001.zip › S1K_Fig.tif]

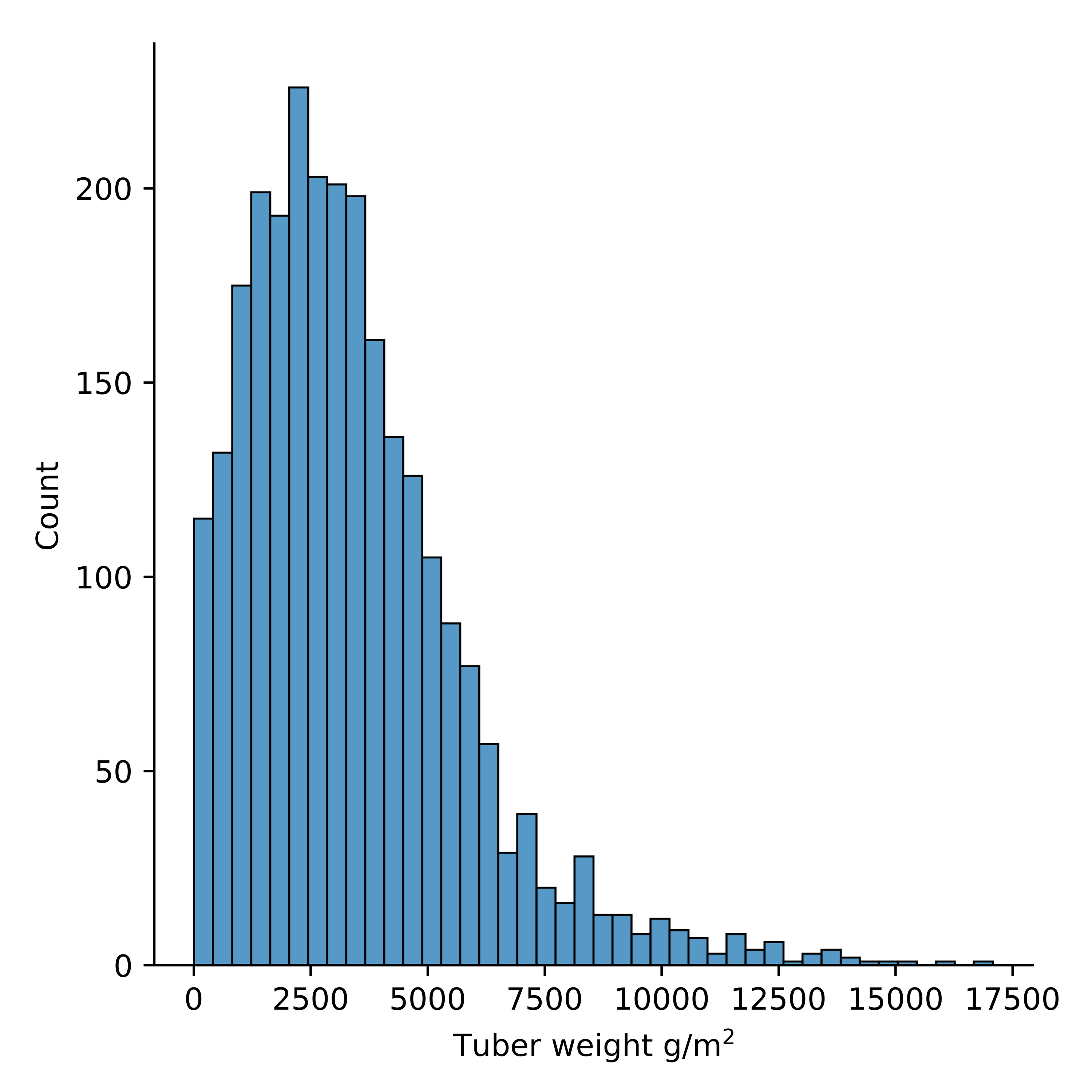

Supplement: S1 Fig — On each field, soil samples were taken. These soil samples are evaluated using the Eurofins protocol, and provide us the amount of the following macro- and micronutrients: N, P, K, Ca, Mg, S, Si, Fe, Zn, Mn, and B. In these histograms, two lines are present as well. The left line represents the lower limit of the advise of Eurofins, and the right line represents the maximum of the range. In addition, some categorical variables are provided. The nutrient content of the field is determined by the farmer’s team, who classifies fields as poor, average or rich. In addition, the field is classified as dry, average or wet by the farmer himself. Potato is a rotation crop; only once per four years, potatoes can be grown on the same field. The crop cultivated before potatoes were grown on the field is the previously cultivated crop. In the “others” category all kinds of crops are captured. Usually, only one or two times, a field is cultivated with that crop. Crops in this category are for example conifers, salsify, or peas. Finally, some fields suffer from nematodes, which can have a negative effect on potato yield. A: N in soil. B: P in soil. C: K in soil. D: Ca in soil. E: Mg in soil. F: Si in soil. G: S in soil. H: Fe in soil. I: Zn in soil. J: Mn in soil. K: B in soil. L: Tuber weight. M: Nutrient content. N: Contains nematodes? O: Year. P: Dryness. Q: Previously cultivated crop. (ZIP) [file pone.0296684.s001.zip › S1L_Fig.tif]

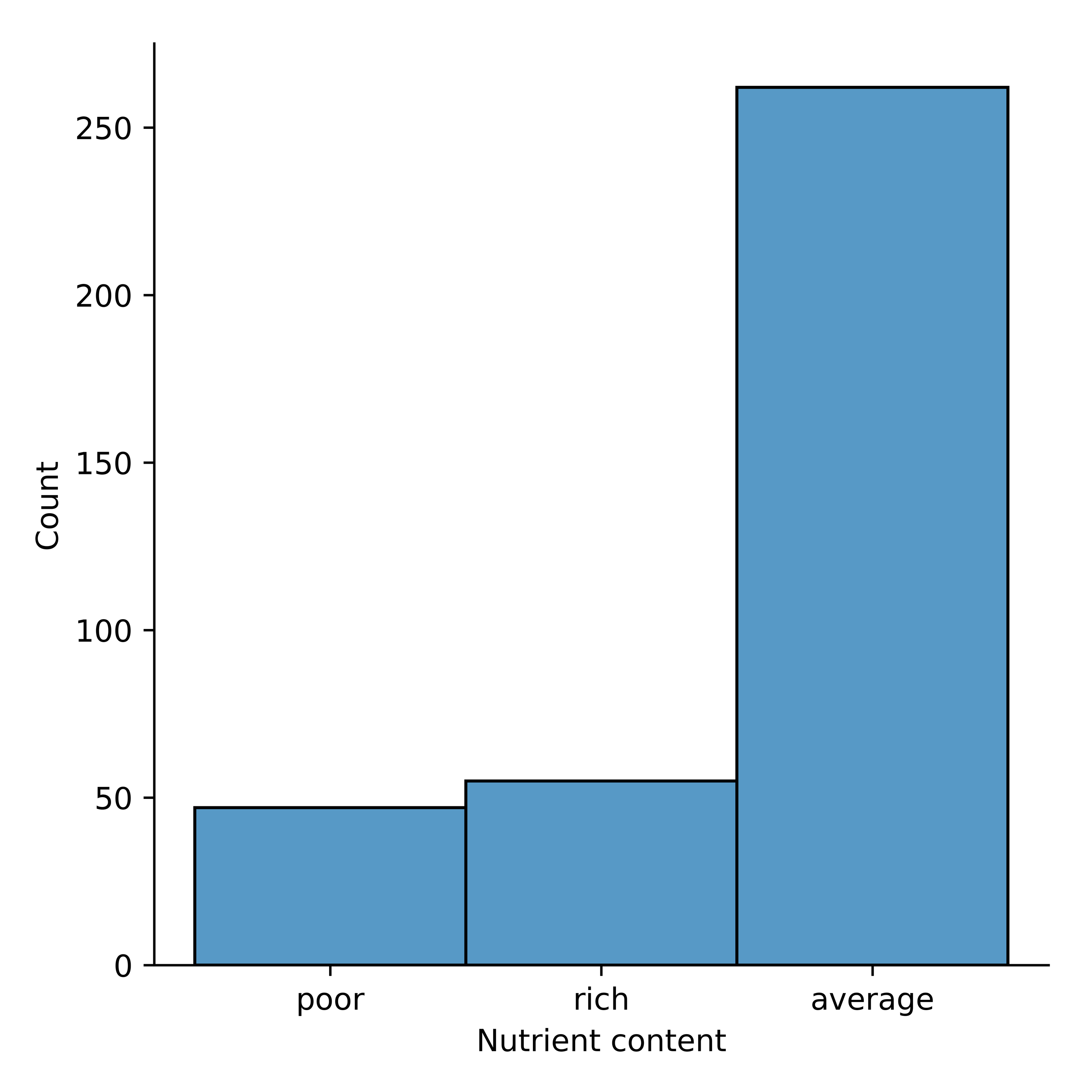

Supplement: S1 Fig — On each field, soil samples were taken. These soil samples are evaluated using the Eurofins protocol, and provide us the amount of the following macro- and micronutrients: N, P, K, Ca, Mg, S, Si, Fe, Zn, Mn, and B. In these histograms, two lines are present as well. The left line represents the lower limit of the advise of Eurofins, and the right line represents the maximum of the range. In addition, some categorical variables are provided. The nutrient content of the field is determined by the farmer’s team, who classifies fields as poor, average or rich. In addition, the field is classified as dry, average or wet by the farmer himself. Potato is a rotation crop; only once per four years, potatoes can be grown on the same field. The crop cultivated before potatoes were grown on the field is the previously cultivated crop. In the “others” category all kinds of crops are captured. Usually, only one or two times, a field is cultivated with that crop. Crops in this category are for example conifers, salsify, or peas. Finally, some fields suffer from nematodes, which can have a negative effect on potato yield. A: N in soil. B: P in soil. C: K in soil. D: Ca in soil. E: Mg in soil. F: Si in soil. G: S in soil. H: Fe in soil. I: Zn in soil. J: Mn in soil. K: B in soil. L: Tuber weight. M: Nutrient content. N: Contains nematodes? O: Year. P: Dryness. Q: Previously cultivated crop. (ZIP) [file pone.0296684.s001.zip › S1M_Fig.tif]

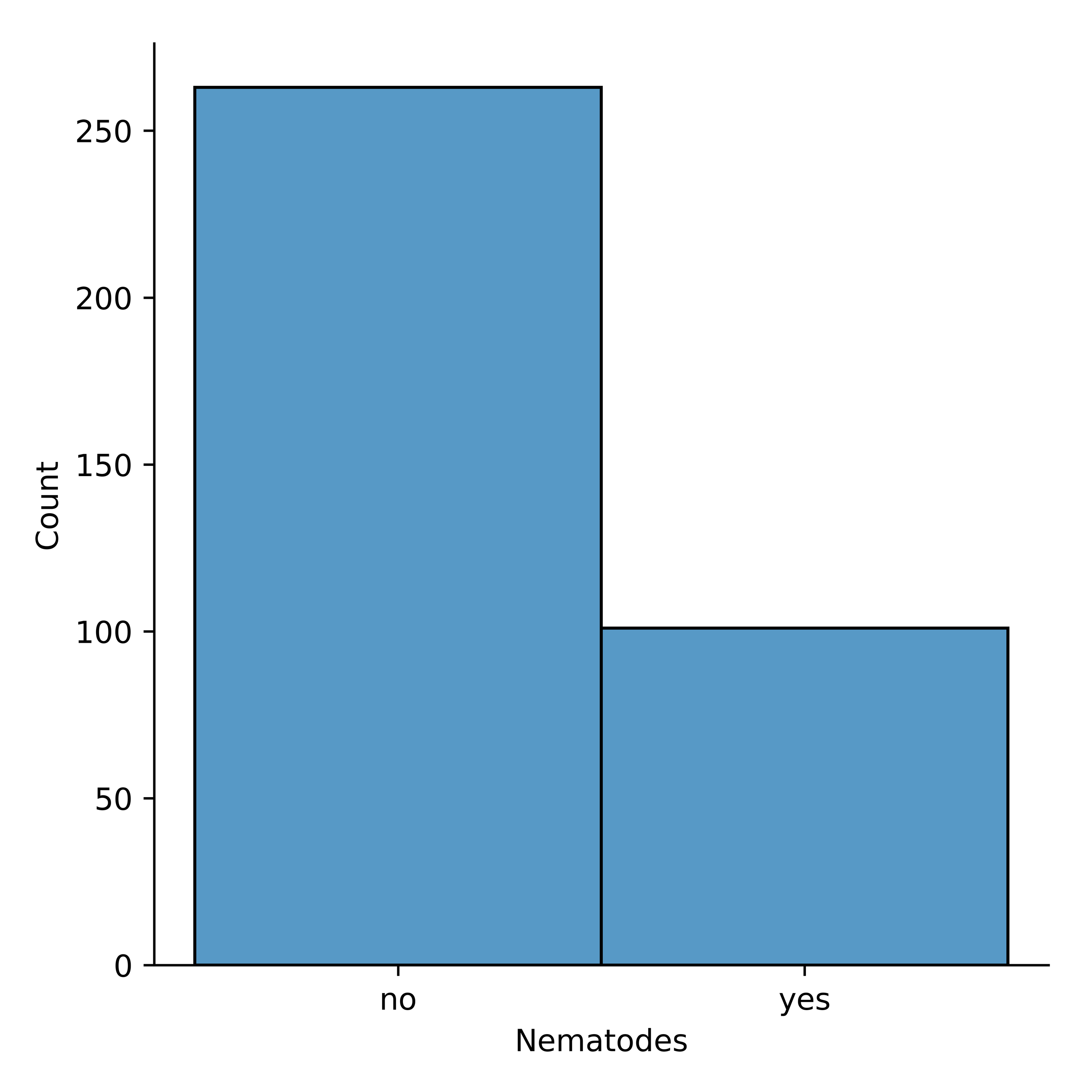

Supplement: S1 Fig — On each field, soil samples were taken. These soil samples are evaluated using the Eurofins protocol, and provide us the amount of the following macro- and micronutrients: N, P, K, Ca, Mg, S, Si, Fe, Zn, Mn, and B. In these histograms, two lines are present as well. The left line represents the lower limit of the advise of Eurofins, and the right line represents the maximum of the range. In addition, some categorical variables are provided. The nutrient content of the field is determined by the farmer’s team, who classifies fields as poor, average or rich. In addition, the field is classified as dry, average or wet by the farmer himself. Potato is a rotation crop; only once per four years, potatoes can be grown on the same field. The crop cultivated before potatoes were grown on the field is the previously cultivated crop. In the “others” category all kinds of crops are captured. Usually, only one or two times, a field is cultivated with that crop. Crops in this category are for example conifers, salsify, or peas. Finally, some fields suffer from nematodes, which can have a negative effect on potato yield. A: N in soil. B: P in soil. C: K in soil. D: Ca in soil. E: Mg in soil. F: Si in soil. G: S in soil. H: Fe in soil. I: Zn in soil. J: Mn in soil. K: B in soil. L: Tuber weight. M: Nutrient content. N: Contains nematodes? O: Year. P: Dryness. Q: Previously cultivated crop. (ZIP) [file pone.0296684.s001.zip › S1N_Fig.tif]

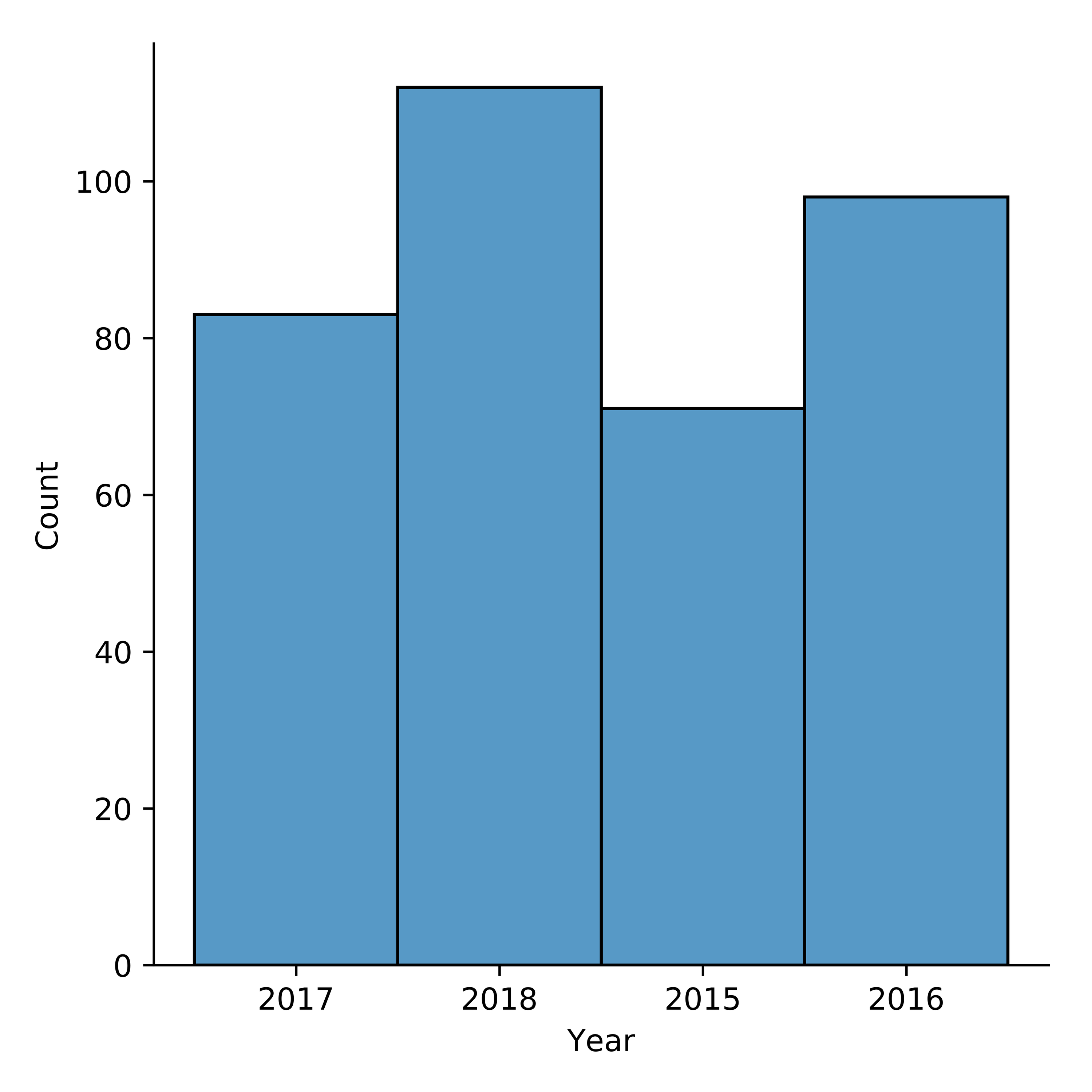

Supplement: S1 Fig — On each field, soil samples were taken. These soil samples are evaluated using the Eurofins protocol, and provide us the amount of the following macro- and micronutrients: N, P, K, Ca, Mg, S, Si, Fe, Zn, Mn, and B. In these histograms, two lines are present as well. The left line represents the lower limit of the advise of Eurofins, and the right line represents the maximum of the range. In addition, some categorical variables are provided. The nutrient content of the field is determined by the farmer’s team, who classifies fields as poor, average or rich. In addition, the field is classified as dry, average or wet by the farmer himself. Potato is a rotation crop; only once per four years, potatoes can be grown on the same field. The crop cultivated before potatoes were grown on the field is the previously cultivated crop. In the “others” category all kinds of crops are captured. Usually, only one or two times, a field is cultivated with that crop. Crops in this category are for example conifers, salsify, or peas. Finally, some fields suffer from nematodes, which can have a negative effect on potato yield. A: N in soil. B: P in soil. C: K in soil. D: Ca in soil. E: Mg in soil. F: Si in soil. G: S in soil. H: Fe in soil. I: Zn in soil. J: Mn in soil. K: B in soil. L: Tuber weight. M: Nutrient content. N: Contains nematodes? O: Year. P: Dryness. Q: Previously cultivated crop. (ZIP) [file pone.0296684.s001.zip › S1O_Fig.tif]

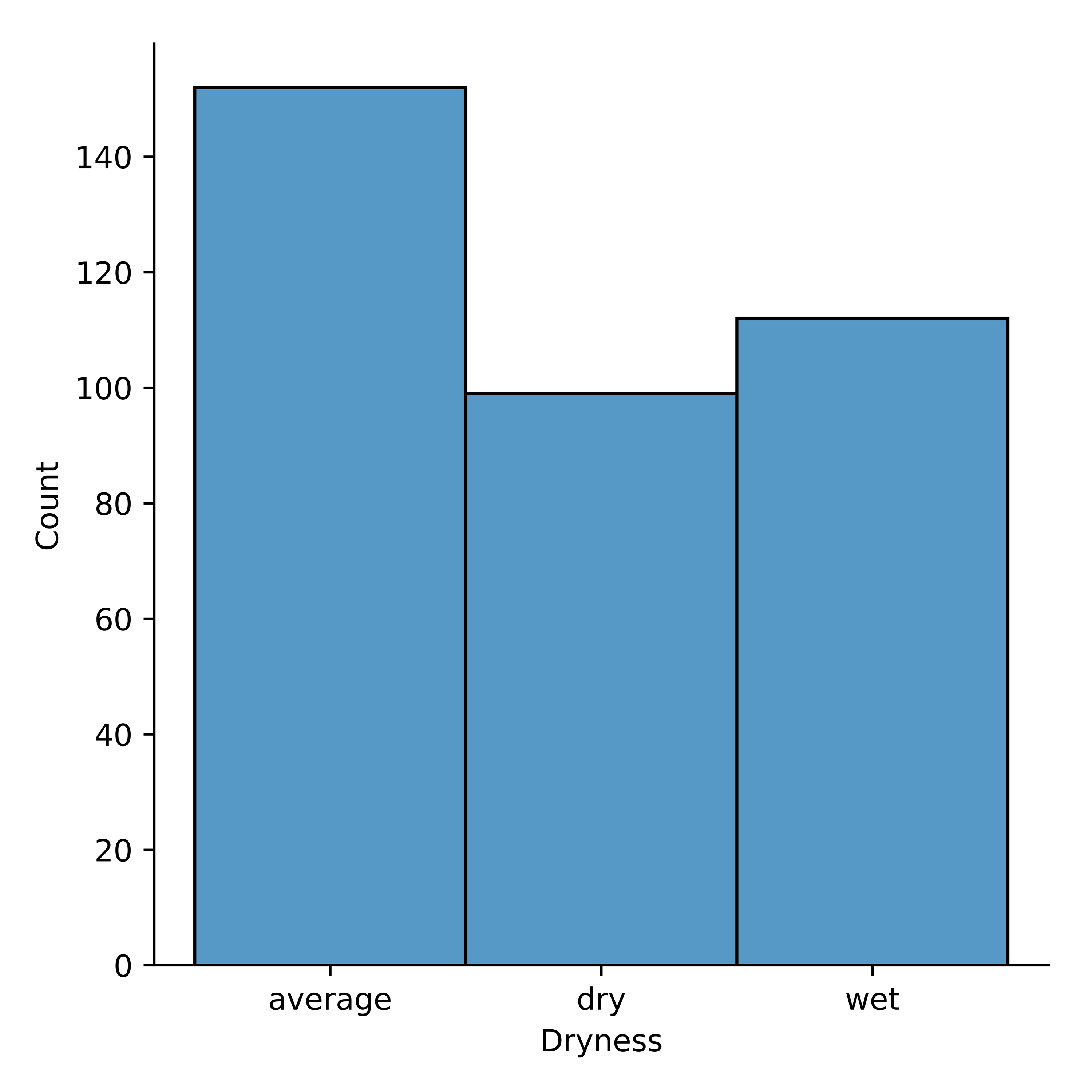

Supplement: S1 Fig — On each field, soil samples were taken. These soil samples are evaluated using the Eurofins protocol, and provide us the amount of the following macro- and micronutrients: N, P, K, Ca, Mg, S, Si, Fe, Zn, Mn, and B. In these histograms, two lines are present as well. The left line represents the lower limit of the advise of Eurofins, and the right line represents the maximum of the range. In addition, some categorical variables are provided. The nutrient content of the field is determined by the farmer’s team, who classifies fields as poor, average or rich. In addition, the field is classified as dry, average or wet by the farmer himself. Potato is a rotation crop; only once per four years, potatoes can be grown on the same field. The crop cultivated before potatoes were grown on the field is the previously cultivated crop. In the “others” category all kinds of crops are captured. Usually, only one or two times, a field is cultivated with that crop. Crops in this category are for example conifers, salsify, or peas. Finally, some fields suffer from nematodes, which can have a negative effect on potato yield. A: N in soil. B: P in soil. C: K in soil. D: Ca in soil. E: Mg in soil. F: Si in soil. G: S in soil. H: Fe in soil. I: Zn in soil. J: Mn in soil. K: B in soil. L: Tuber weight. M: Nutrient content. N: Contains nematodes? O: Year. P: Dryness. Q: Previously cultivated crop. (ZIP) [file pone.0296684.s001.zip › S1P_Fig.tif]

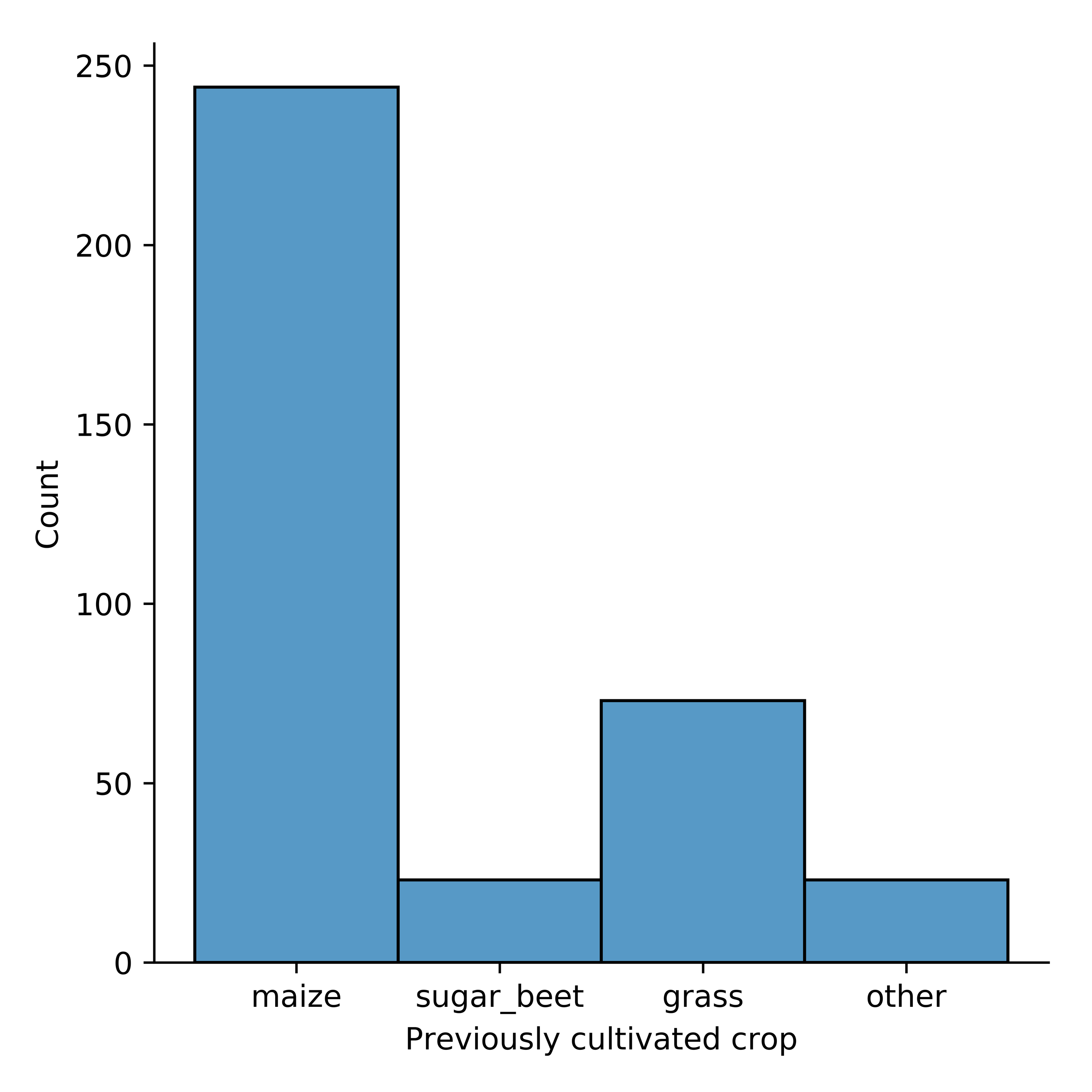

Supplement: S1 Fig — On each field, soil samples were taken. These soil samples are evaluated using the Eurofins protocol, and provide us the amount of the following macro- and micronutrients: N, P, K, Ca, Mg, S, Si, Fe, Zn, Mn, and B. In these histograms, two lines are present as well. The left line represents the lower limit of the advise of Eurofins, and the right line represents the maximum of the range. In addition, some categorical variables are provided. The nutrient content of the field is determined by the farmer’s team, who classifies fields as poor, average or rich. In addition, the field is classified as dry, average or wet by the farmer himself. Potato is a rotation crop; only once per four years, potatoes can be grown on the same field. The crop cultivated before potatoes were grown on the field is the previously cultivated crop. In the “others” category all kinds of crops are captured. Usually, only one or two times, a field is cultivated with that crop. Crops in this category are for example conifers, salsify, or peas. Finally, some fields suffer from nematodes, which can have a negative effect on potato yield. A: N in soil. B: P in soil. C: K in soil. D: Ca in soil. E: Mg in soil. F: Si in soil. G: S in soil. H: Fe in soil. I: Zn in soil. J: Mn in soil. K: B in soil. L: Tuber weight. M: Nutrient content. N: Contains nematodes? O: Year. P: Dryness. Q: Previously cultivated crop. (ZIP) [file pone.0296684.s001.zip › S1Q_Fig.tif]
