# Supplementary material for: Introducing exceptional growth mining—Analyzing the impact of soil characteristics on on-farm crop growth and yield variability
Source: PLoS One. 2024 Jan 29;19(1):e0296684. doi: 10.1371/journal.pone.0296684 (PMC10824435; doi:10.1371/journal.pone.0296684)
Supplement: S1 Appendix — (PDF) [file pone.0296684.s002.pdf]

```
proc nlmixed data=mydata tech=trureg maxiter=150 maxfunc=3000;
        parms alpha15=8.5 alpha16=8.5 alpha17=9 alpha18=8.5 var_a=0.3
                beta15=1 beta16=0.9 beta17=0.6 beta18=0.6  var_b=0.3
                gamma15=2.5 gamma16=2.5 gamma17=3.5 gamma18=2.5 var_c=-1
                ve=0.1  rho_ab=0.05 rho_ac=0.05 rho_bc=-0.55;
        bounds alpha15 > 0, alpha16>0, alpha17>0, alpha18>0, ve>0,  var_a > 0,
        rho_ab >= -1, rho_ab <= 1 , rho_ac >= -1,  rho_ac <= 1, rho_bc >= -1 , rho_bc <= 1 ;
        MU = alpha15*(year=2015) + alpha16*(year=2016) + alpha17*(year=2017) + alpha18*(year=2018) + var_a*a
                - log(1+exp(-exp(beta15*(year=2015) + beta16*(year=2016) + beta17*(year=2017)
                + beta18*(year=2018) + (var_b)*b )*(day-gamma15*(year=2015) - gamma16*(year=2016)
                - gamma17*(year=2017) - gamma18*(year=2018)   - (exp(var_c))*c  )));
        model log_tuber_weight ~ normal(mu,ve);
        random a b c ~ normal([0,0,0],[1,rho_ab,1,rho_ac,rho_bc,1]) subject=name;
        predict a out=predsalpha (rename = (pred = a));
        predict b out=predsbeta (rename = (pred = b));
        predict c out=predsgamma (rename = (pred = c));
        predict mu out=predsmu;
        ods output ParameterEstimates= parms;
run;
```
