# Supplementary material for: Introducing exceptional growth mining—Analyzing the impact of soil characteristics on on-farm crop growth and yield variability
Source: PLoS One. 2024 Jan 29;19(1):e0296684. doi: 10.1371/journal.pone.0296684 (PMC10824435; doi:10.1371/journal.pone.0296684)
Supplement: S1 Table — (PDF) [file pone.0296684.s003.pdf]

	N_soil	S_soil	K_soil	Mg_soil	Si_soil	Fe_soil	Zn_soil	Ca_soil	Mn_soil	B_soil	P_soil
N_soil	1	0.549	0.475	0.238	0.188	-0.081	0.144	0.003	0.328	0.350	0.244
S_soil	0.549	1	0.298	0.124	0.144	-0.061	-0.011	0.072	0.107	0.334	0.180
K_soil	0.475	0.298	1	0.115	0.162	-0.031	0.004	0.746	0.108	-0.026	0.276
Mg_soil	0.238	0.124	0.115	1	0.086	-0.039	-0.389	-0.018	-0.302	0.017	0.005
Si_soil	0.188	0.144	0.162	0.086	1	0.326	0.054	0.152	0.085	0.176	0.081
Fe_soil	0.081	0.061	-0.031	-0.039	0.326	1	0.044	0.152	0.048	-0.001	-0.124
Zn_soil	0.144	0.011	0.004	-0.389	0.054	0.044	1	-0.082	0.648	0.015	0.230
Ca_soil	0.003	0.072	0.746	-0.018	0.152	0.152	-0.082	1	-0.027	-0.215	0.128
Mn_soil	0.328	0.107	0.108	-0.302	0.085	0.048	0.648	-0.027	1	0.048	0.166
B_soil	0.350	0.334	-0.026	0.017	0.176	-0.001	0.015	-0.215	0.048	1	0.104
P_soil	0.244	0.180	0.276	0.005	0.081	-0.124	0.230	0.128	0.166	0.104	1
