# Supplementary material for: Introducing exceptional growth mining—Analyzing the impact of soil characteristics on on-farm crop growth and yield variability
Source: PLoS One. 2024 Jan 29;19(1):e0296684. doi: 10.1371/journal.pone.0296684 (PMC10824435; doi:10.1371/journal.pone.0296684)
Supplement: S2 Table — (PDF) [file pone.0296684.s004.pdf]

$\varphi^{u}_{\mathrm{GC}_h}$	Description	Mean	Std	Number of fields					Yield
				Total	2015	2016	2017	2018	
4.97	N_soil $\leq$ 40.2 $\wedge$ Dryness = wet	0.81	0.51	10	0	4	5	1	45.5
4.53	B_soil > 1113.6 $\wedge$ K_soil > 165.5	0.42	0.45	24	5	3	11	5	58.5
4.25	Ca_soil > 196.8 $\wedge$ Zn_soil $\leq$ 2055.6	0.50	0.52	19	0	7	4	8	45.1
4.07	Si_soil > 13.0 $\wedge$ P_soil > 7.5	0.48	0.37	10	2	2	2	4	55.8
3.86	B_soil > 1113.6 $\wedge$ K_soil > 257.3	0.44	0.39	12	1	1	5	5	55.3
3.83	K_soil > 308.1 $\wedge$ Zn_soil $\leq$ 2082.0	0.39	0.51	25	3	6	5	11	49.9
3.73	Zn_soil $\leq$ 1396.8 $\wedge$ K_soil > 348.8	0.53	0.49	12	2	2	2	6	50.1
3.66	Fe_soil > 324.0 $\wedge$ Fe_soil $\leq$ 444.0	0.30	0.56	47	10	12	12	13	49.0
3.61	Ca_soil > 196.8 $\wedge$ Mn_soil $\leq$ 798.0	0.56	0.51	11	0	2	2	7	46.0
3.54	B_soil $\leq$ 366.0 $\wedge$ Dryness = wet	0.34	0.48	26	7	1	6	12	50.2
3.42	Fe_soil $\leq$ 145.2 $\wedge$ Nutrient_content = rich	0.43	0.34	7	2	2	3	0	68.1
3.42	K_soil > 308.1 $\wedge$ Mn_soil $\leq$ 1458.0	0.34	0.50	25	5	4	3	13	50.4
3.41	B_soil > 1113.6 $\wedge$ B_soil $\leq$ 1398.0	0.52	0.55	13	1	4	7	1	53.8
3.33	K_soil > 308.1 $\wedge$ P_soil $\leq$ 6.0	0.31	0.56	37	6	4	9	18	51.7
3.23	K_soil > 146.1 $\wedge$ Mg_soil > 278.6	0.25	0.47	36	11	4	11	10	54.6
3.20	Dryness = wet $\wedge$ B_soil $\leq$ 330.0	0.28	0.37	18	6	1	5	6	53.5
3.02	K_soil > 308.1 $\wedge$ P_soil $\leq$ 10.1	0.24	0.55	48	8	7	12	21	50.9
3.02	Mn_soil $\leq$ 1454.4 $\wedge$ S_soil > 19.2	0.24	0.54	46	12	5	14	15	50.9
2.96	Zn_soil $\leq$ 3906.0 $\wedge$ B_soil > 1026.0	0.27	0.54	35	6	7	16	6	52.1
2.94	P_soil $\leq$ 7.4 $\wedge$ K_soil > 285.2	0.23	0.54	48	8	5	13	22	51.7
2.90	Dryness = wet $\wedge$ Mn_soil $\leq$ 1320.0	0.36	0.73	35	3	6	13	13	45.1
2.90	K_soil > 308.1 $\wedge$ Nutrient_content $\neq$ poor	0.22	0.53	51	7	8	16	20	51.7
2.89	Dryness = wet $\wedge$ Mn_soil $\leq$ 3216.0	0.29	0.73	52	7	10	17	18	46.4
2.89	Dryness = wet $\wedge$ Fe_soil $\leq$ 594.0	0.26	0.74	69	8	14	21	26	44.8
2.87	K_soil > 308.1 $\wedge$ Fe_soil > 192.0	0.26	0.55	36	7	6	6	17	50.9
2.85	Nutrient_content $\neq$ poor $\wedge$ K_soil > 303.4	0.21	0.53	52	7	8	16	21	51.5
2.84	K_soil > 308.1 $\wedge$ Nutrient_content = average	0.23	0.52	42	6	8	10	18	50.3
2.78	Dryness $\neq$ dry $\wedge$ K_soil > 196.9	0.20	0.65	85	16	10	20	39	49.5
2.78	Nutrient_content = average $\wedge$ K_soil > 304.8	0.22	0.52	43	6	8	10	19	50.1
2.75	Dryness = wet $\wedge$ Previously_cultivated_crop = maize	0.21	0.70	85	11	25	20	29	43.2
2.75	Previously_cultivated_crop = maize $\wedge$ Dryness = wet	0.21	0.70	85	11	25	20	29	43.2
2.73	Mn_soil $\leq$ 1454.4 $\wedge$ Dryness = wet	0.33	0.73	36	3	6	13	14	44.6
2.72	S_soil $\leq$ 34.0 $\wedge$ Dryness = wet	0.26	0.77	65	9	15	16	25	43.5
2.71	N_soil $\leq$ 138.2 $\wedge$ Dryness = wet	0.28	0.76	56	7	15	19	15	45.0
2.70	N_soil > 220.8 $\wedge$ S_soil > 60.0	0.29	0.38	12	5	0	2	5	58.3
2.67	Dryness = wet $\wedge$ Si_soil $\leq$ 12.0	0.28	0.77	53	10	8	18	17	47.9
2.64	Si_soil > 6.0 $\wedge$ Dryness = wet	0.23	0.72	71	8	15	22	26	44.8
2.59	Dryness = wet $\wedge$ Si_soil > 7.0	0.23	0.71	66	8	14	20	24	45.0
2.57	Fe_soil $\leq$ 472.8 $\wedge$ Fe_soil > 252.0	0.16	0.59	93	22	21	20	30	48.8
2.54	Dryness = wet $\wedge$ Si_soil $\leq$ 15.0	0.24	0.79	70	10	12	22	26	46.2
2.52	K_soil > 207.5 $\wedge$ Dryness $\neq$ dry	0.18	0.64	77	14	7	19	37	49.7
2.49	Previously_cultivated_crop $\neq$ maize $\wedge$ N_soil > 219.6	0.25	0.45	20	3	1	9	7	58.8
2.45	S_soil > 22.8 $\wedge$ K_soil > 178.4	0.17	0.59	70	10	4	26	30	52.1
2.45	S_soil > 34.0 $\wedge$ Mg_soil > 302.6	0.26	0.36	12	4	1	6	1	60.4
2.44	Dryness = wet $\wedge$ Previously_cultivated_crop $\neq$ grass	0.18	0.73	95	13	27	21	34	43.2
2.44	Previously_cultivated_crop $\neq$ grass $\wedge$ Dryness = wet	0.18	0.73	95	13	27	21	34	43.2

Yield is reported in ton ha^{-1}, N, P, K, Ca and Mg are reported in kg ha^{-1} and B, Fe, Mn and Zn are reported g ha^{-1}.
