# Supplementary material for: Introducing exceptional growth mining—Analyzing the impact of soil characteristics on on-farm crop growth and yield variability
Source: PLoS One. 2024 Jan 29;19(1):e0296684. doi: 10.1371/journal.pone.0296684 (PMC10824435; doi:10.1371/journal.pone.0296684)
Supplement: S3 Table — (PDF) [file pone.0296684.s005.pdf]

$\varphi^{u}_{\mathrm{GC}_h}$	Description	Mean	Std	Number of fields					Yield
				Total	2015	2016	2017	2018	
7.21	Mg_soil ≤ 158.5 ∧ P_soil > 6.6 ∧ Ca_soil ≤ 229.8	-0.37	0.13	6	0	1	1	4	44.7
5.47	Fe_soil ≤ 145.2 ∧ Mn_soil > 3770.4 ∧ Zn_soil ≤ 5020.8	-0.94	0.45	7	2	0	3	2	42.0
5.40	Mg_soil ≤ 158.5 ∧ P_soil > 6.6 ∧ Ca_soil ≤ 491.8	-0.31	0.16	8	0	1	2	5	42.8
5.38	Mg_soil ≤ 158.5 ∧ P_soil > 6.6 ∧ Si_soil > 7.2	-0.31	0.16	8	1	1	2	4	46.7
5.07	Dryness =average ∧ Zn_soil > 6868.8 ∧ Zn_soil ≤ 9582.0	-0.97	0.60	10	0	5	3	2	36.0
5.05	Mg_soil ≤ 158.5 ∧ Zn_soil > 2829.6 ∧ Ca_soil > 182.0	-0.36	0.20	8	0	0	2	6	38.1
4.94	Zn_soil > 7294.8 ∧ Dryness =average ∧ Zn_soil ≤ 9724.8	-0.90	0.58	10	0	5	2	3	35.8
4.89	Mn_soil > 7648.8 ∧ Si_soil ≤ 7.2 ∧ Fe_soil ≤ 261.6	-1.14	0.66	8	1	1	4	2	46.6
4.87	P_soil ≤ 1.2 ∧ Dryness =average ∧ Ca_soil ≤ 118.9	-0.79	0.61	14	0	9	2	3	39.8
4.87	Dryness =average ∧ P_soil ≤ 1.3 ∧ Ca_soil ≤ 118.9	-0.79	0.61	14	0	9	2	3	39.8
4.83	Dryness =average ∧ Zn_soil > 6868.8 ∧ Zn_soil ≤ 11569.2	-0.79	0.64	15	1	8	3	3	39.5
4.77	Fe_soil ≤ 145.2 ∧ Mn_soil > 3770.4 ∧ K_soil ≤ 227.4	-0.95	0.52	7	1	1	4	1	43.7
4.70	P_soil ≤ 1.2 ∧ Dryness =average ∧ Ca_soil ≤ 34.4	-1.01	0.57	7	0	4	2	1	40.4
4.70	Dryness =average ∧ P_soil ≤ 1.3 ∧ Ca_soil ≤ 34.4	-1.01	0.57	7	0	4	2	1	40.4
4.66	S_soil ≤ 22.8 ∧ Dryness ≠wet ∧ Fe_soil > 446.4	-0.52	0.58	27	10	10	4	3	46.9
4.65	Mn_soil > 7648.8 ∧ Si_soil ≤ 7.2 ∧ Nematodes =no	-1.17	0.71	8	2	1	3	2	44.6
4.65	Mn_soil > 7648.8 ∧ Si_soil ≤ 7.2 ∧ Nematodes ≠yes	-1.17	0.71	8	2	1	3	2	44.6
4.62	K_soil ≤ 308.1 ∧ Dryness ≠wet ∧ Nutrient_content =poor	-0.60	0.65	25	5	5	8	7	46.7
4.62	Dryness ≠wet ∧ K_soil ≤ 302.8 ∧ Nutrient_content =poor	-0.60	0.65	25	5	5	8	7	46.7
4.62	Nutrient_content =poor ∧ Dryness ≠wet ∧ K_soil ≤ 215.0	-0.60	0.65	25	5	5	8	7	46.7
4.62	Dryness ≠wet ∧ Nutrient_content =poor ∧ K_soil ≤ 215.0	-0.60	0.65	25	5	5	8	7	46.7
4.62	K_soil ≤ 308.1 ∧ Nutrient_content =poor ∧ Dryness ≠wet	-0.60	0.65	25	5	5	8	7	46.7
4.59	B_soil > 564.0 ∧ Mn_soil > 9231.6 ∧ Nutrient_content =poor	-1.08	0.58	6	2	1	2	1	51.1
4.58	N_soil ≤ 138.2 ∧ Dryness ≠wet ∧ Nutrient_content =poor	-0.66	0.66	21	4	5	5	7	44.1
4.54	Dryness ≠wet ∧ S_soil ≤ 32.8 ∧ Mg_soil ≤ 201.0	-0.36	0.65	69	13	25	15	16	47.2
4.54	S_soil ≤ 34.0 ∧ Dryness ≠wet ∧ Mg_soil ≤ 201.8	-0.36	0.65	69	13	25	15	16	47.2
4.48	Mn_soil > 7648.8 ∧ Mn_soil > 9234.0 ∧ K_soil ≤ 375.3	-0.48	0.67	38	8	10	8	12	45.6
4.48	Mn_soil > 3123.6 ∧ Mn_soil > 9234.0 ∧ K_soil ≤ 375.3	-0.48	0.67	38	8	10	8	12	45.6
4.48	Zn_soil > 1396.8 ∧ Mn_soil > 9234.0 ∧ K_soil ≤ 375.3	-0.48	0.67	38	8	10	8	12	45.6
4.48	Mn_soil > 717.6 ∧ Mn_soil > 9234.0 ∧ K_soil ≤ 375.3	-0.48	0.67	38	8	10	8	12	45.6
4.46	Nutrient_content ≠average ∧ Zn_soil > 2836.8 ∧ Fe_soil > 442.8	-0.75	0.56	11	4	4	3	0	43.6
4.42	Nutrient_content =poor ∧ B_soil > 440.4 ∧ S_soil ≤ 46.2	-0.99	0.81	13	3	3	5	2	45.1
4.41	Dryness ≠wet ∧ S_soil ≤ 22.0 ∧ Fe_soil > 451.2	-0.49	0.56	26	10	10	3	3	47.6
4.33	Mg_soil ≤ 199.2 ∧ Dryness ≠wet ∧ S_soil ≤ 30.4	-0.34	0.64	66	13	22	15	16	47.7
4.32	Mg_soil ≤ 238.6 ∧ Mn_soil > 10110.0 ∧ Ca_soil > 86.0	-0.22	0.12	6	0	2	2	2	54.4
4.32	Mn_soil > 7648.8 ∧ Si_soil ≤ 10.0 ∧ B_soil > 787.2	-0.86	0.69	12	4	3	3	2	48.7
4.31	Mn_soil > 7648.8 ∧ Mn_soil > 9234.0 ∧ Zn_soil ≤ 11145.6	-0.56	0.70	29	7	6	7	9	49.2
4.31	Mn_soil > 3123.6 ∧ Mn_soil > 9234.0 ∧ Zn_soil ≤ 11145.6	-0.56	0.70	29	7	6	7	9	49.2
4.31	Zn_soil > 1396.8 ∧ Mn_soil > 9234.0 ∧ Zn_soil ≤ 11145.6	-0.56	0.70	29	7	6	7	9	49.2
4.31	Mn_soil > 717.6 ∧ Mn_soil > 9234.0 ∧ Zn_soil ≤ 11145.6	-0.56	0.70	29	7	6	7	9	49.2
4.28	Nutrient_content ≠average ∧ Mn_soil > 9394.8 ∧ K_soil > 137.9	-0.80	0.62	11	3	1	4	3	48.5
4.27	Mn_soil > 7648.8 ∧ Nutrient_content ≠average ∧ Si_soil ≤ 7.2	-1.10	0.63	6	3	0	2	1	50.4
4.27	Mn_soil > 7648.8 ∧ Si_soil ≤ 7.2 ∧ Nutrient_content ≠average	-1.10	0.63	6	3	0	2	1	50.4
4.25	K_soil ≤ 308.1 ∧ Mn_soil > 7644.0 ∧ Mn_soil > 9013.2	-0.46	0.66	37	8	11	8	10	45.7
4.24	Nutrient_content =poor ∧ Dryness ≠wet ∧ N_soil ≤ 121.0	-0.67	0.69	19	4	5	4	6	44.4
4.24	Dryness ≠wet ∧ Nutrient_content =poor ∧ N_soil ≤ 121.0	-0.67	0.69	19	4	5	4	6	44.4
4.22	Dryness ≠wet ∧ S_soil ≤ 22.0 ∧ Nutrient_content ≠rich	-0.28	0.71	111	25	37	20	29	48.4
4.21	S_soil ≤ 16.8 ∧ Dryness ≠wet ∧ Fe_soil > 454.8	-0.56	0.59	20	6	8	3	3	45.5
4.21	Mn_soil > 7648.8 ∧ B_soil > 706.8 ∧ Nutrient_content ≠average	-0.73	0.60	12	3	2	6	1	53.7
4.19	Mg_soil ≤ 158.5 ∧ Fe_soil > 477.6 ∧ Zn_soil ≤ 13552.8	-0.75	0.54	9	4	4	0	1	48.0
4.19	Mg_soil ≤ 158.5 ∧ Fe_soil > 477.6 ∧ Fe_soil ≤ 878.4	-0.75	0.54	9	4	4	0	1	48.0
4.18	Fe_soil ≤ 145.2 ∧ Mn_soil > 3770.4 ∧ Zn_soil ≤ 9006.0	-0.80	0.58	9	2	1	4	2	43.1
4.17	Mn_soil > 7648.8 ∧ Fe_soil ≤ 255.6 ∧ Si_soil ≤ 10.0	-0.67	0.68	18	3	2	7	6	46.8
4.17	Mn_soil > 7648.8 ∧ Si_soil ≤ 10.0 ∧ Fe_soil ≤ 256.8	-0.67	0.68	18	3	2	7	6	46.8
4.17	Si_soil ≤ 8.4 ∧ Nutrient_content =poor ∧ Dryness ≠wet	-0.64	0.66	18	3	3	5	7	47.5
4.16	Fe_soil ≤ 145.2 ∧ Mn_soil > 3770.4 ∧ B_soil ≤ 687.6	-0.71	0.45	7	2	0	3	2	42.1
4.11	Dryness ≠wet ∧ S_soil ≤ 16.0 ∧ Fe_soil > 451.2	-0.59	0.61	18	6	7	2	3	42.7
4.11	Dryness =average ∧ Zn_soil > 6868.8 ∧ S_soil ≤ 31.4	-0.53	0.58	20	2	10	4	4	42.9
4.11	Si_soil ≤ 8.4 ∧ Mn_soil > 6020.4 ∧ Mg_soil ≤ 216.0	-0.74	0.79	19	4	6	5	4	43.5
4.11	Mn_soil > 7648.8 ∧ Mn_soil > 9234.0 ∧ Dryness ≠wet	-0.43	0.62	35	7	8	12	8	51.9
4.11	Mn_soil > 3123.6 ∧ Mn_soil > 9234.0 ∧ Dryness ≠wet	-0.43	0.62	35	7	8	12	8	51.9
4.11	Zn_soil > 1396.8 ∧ Mn_soil > 9234.0 ∧ Dryness ≠wet	-0.43	0.62	35	7	8	12	8	51.9
4.11	Mn_soil > 717.6 ∧ Mn_soil > 9234.0 ∧ Dryness ≠wet	-0.43	0.62	35	7	8	12	8	51.9
4.09	Mn_soil > 7648.8 ∧ Mn_soil > 9234.0 ∧ B_soil > 366.0	-0.51	0.66	28	4	7	10	7	48.3
4.09	Mn_soil > 3123.6 ∧ Mn_soil > 9234.0 ∧ B_soil > 366.0	-0.51	0.66	28	4	7	10	7	48.3
4.09	Zn_soil > 1396.8 ∧ Mn_soil > 9234.0 ∧ B_soil > 366.0	-0.51	0.66	28	4	7	10	7	48.3
4.09	Mn_soil > 717.6 ∧ Mn_soil > 9234.0 ∧ B_soil > 366.0	-0.51	0.66	28	4	7	10	7	48.3

Yield is reported in ton ha^{-1}, N, P, K, Ca and Mg are reported in kg ha^{-1} and B, Fe, Mn and Zn are reported g ha^{-1}.
