# Supplementary material for: Introducing exceptional growth mining—Analyzing the impact of soil characteristics on on-farm crop growth and yield variability
Source: PLoS One. 2024 Jan 29;19(1):e0296684. doi: 10.1371/journal.pone.0296684 (PMC10824435; doi:10.1371/journal.pone.0296684)
Supplement: S4 Table — (PDF) [file pone.0296684.s006.pdf]

$\varphi^u_{\mathrm{GC}_h}$	Description	Mean	Std	Number of fields					Yield
				Total	2015	2016	2017	2018	
11.82	Dryness =dry ∧ Mn_soil > 6405.6 ∧ Zn_soil ≤ 9424.8	0.56	0.13	7	1	1	3	2	59.0
7.96	P_soil > 7.4 ∧ K_soil ≤ 142.2 ∧ Fe_soil > 100.8	0.45	0.17	9	2	6	0	1	50.4
6.86	Dryness =dry ∧ Mn_soil > 6405.6 ∧ Zn_soil ≤ 10897.2	0.43	0.21	11	2	1	6	2	58.2
6.83	Nutrient_content ≠average ∧ Si_soil > 12.4 ∧ Ca_soil ≤ 1.7	0.87	0.34	7	0	3	2	2	36.2
6.00	Dryness =dry ∧ Mn_soil > 6405.6 ∧ Mg_soil > 111.5	0.44	0.27	14	5	1	6	2	55.6
5.74	Dryness =dry ∧ Mn_soil > 6405.6 ∧ Mg_soil > 159.5	0.58	0.27	7	2	1	3	1	59.4
5.58	Dryness =dry ∧ Mn_soil > 6405.6 ∧ S_soil > 16.4	0.53	0.36	14	4	1	7	2	59.3
5.56	Dryness =dry ∧ Mn_soil > 6405.6 ∧ Nutrient_content ≠rich	0.46	0.30	13	3	1	6	3	57.1
5.54	Dryness =dry ∧ Mn_soil > 6405.6 ∧ S_soil > 22.6	0.55	0.33	11	2	1	6	2	57.9
5.33	Dryness =dry ∧ Mn_soil > 6405.6 ∧ Mg_soil > 142.6	0.46	0.29	11	4	1	5	1	59.8
5.18	B_soil > 564.0 ∧ N_soil > 174.2 ∧ K_soil ≤ 183.1	0.66	0.55	19	5	5	7	2	47.4
5.15	Mn_soil > 7648.8 ∧ Zn_soil ≤ 13458.0 ∧ P_soil > 7.6	0.45	0.27	10	3	3	2	2	44.1
5.15	S_soil > 11.4 ∧ Dryness =average ∧ K_soil ≤ 166.2	0.53	0.63	37	8	12	9	8	45.2
5.12	Nutrient_content ≠average ∧ Si_soil > 12.4 ∧ S_soil > 22.5	0.90	0.47	7	1	2	2	2	44.6
5.12	Nutrient_content ≠average ∧ Si_soil > 12.4 ∧ N_soil > 159.0	0.90	0.47	7	1	2	2	2	44.6
4.92	Si_soil ≤ 6.0 ∧ Zn_soil ≤ 1429.2 ∧ Mn_soil ≤ 534.0	0.52	0.30	8	4	3	0	1	56.0
4.91	Si_soil ≤ 6.0 ∧ Zn_soil ≤ 1429.2 ∧ Fe_soil > 110.4	0.49	0.31	10	4	3	0	3	53.1
4.84	Dryness =average ∧ S_soil > 10.0 ∧ K_soil ≤ 166.7	0.51	0.64	38	9	12	9	8	45.4
4.74	Si_soil ≤ 6.0 ∧ Zn_soil ≤ 1429.2 ∧ Dryness =average	0.90	0.46	6	3	2	0	1	53.4
4.63	P_soil > 7.4 ∧ K_soil ≤ 142.2 ∧ B_soil > 271.2	0.57	0.37	9	2	6	1	0	49.8
4.62	K_soil ≤ 146.1 ∧ Dryness =average ∧ N_soil > 101.6	0.50	0.51	22	8	4	6	4	50.1
4.59	K_soil ≤ 146.1 ∧ Dryness =average ∧ B_soil > 1554.0	0.76	0.55	11	3	4	4	0	44.2
4.58	Dryness =average ∧ K_soil ≤ 274.7 ∧ B_soil > 453.6	0.51	0.69	39	11	14	11	3	47.0
4.53	B_soil > 564.0 ∧ N_soil > 174.2 ∧ S_soil ≤ 42.2	0.46	0.54	28	5	3	11	9	48.2
4.51	K_soil ≤ 308.1 ∧ Dryness =average ∧ B_soil > 487.2	0.48	0.68	41	11	13	12	5	47.3
4.50	Dryness =average ∧ B_soil > 346.8 ∧ N_soil > 36.4	0.38	0.65	58	16	9	17	16	50.6
4.47	S_soil ≤ 34.0 ∧ Dryness =average ∧ B_soil > 453.6	0.44	0.63	41	10	14	10	7	48.0
4.43	Dryness =average ∧ K_soil ≤ 195.9 ∧ N_soil > 107.0	0.47	0.58	30	11	4	9	6	50.7
4.42	B_soil > 564.0 ∧ N_soil > 119.0 ∧ K_soil ≤ 167.3	0.52	0.63	28	7	8	9	4	48.0
4.42	K_soil ≤ 207.5 ∧ Dryness ≠wet ∧ B_soil > 192.0	0.32	0.72	97	24	33	23	17	49.9
4.42	Mn_soil > 7648.8 ∧ Zn_soil ≤ 13458.0 ∧ Dryness ≠wet	0.43	0.58	35	8	9	11	7	51.8
4.41	K_soil ≤ 308.1 ∧ Dryness ≠wet ∧ B_soil > 195.6	0.27	0.71	135	31	39	35	30	50.6
4.40	Dryness =average ∧ S_soil ≤ 32.2 ∧ B_soil > 435.6	0.47	0.66	39	10	15	9	5	47.1
4.40	B_soil > 252.0 ∧ Dryness =average ∧ B_soil > 440.4	0.41	0.70	56	13	14	16	13	49.4
4.38	Dryness ≠wet ∧ K_soil ≤ 210.4 ∧ B_soil > 192.0	0.32	0.72	99	25	34	23	17	50.3
4.33	K_soil ≤ 308.1 ∧ Dryness =average ∧ B_soil > 223.2	0.33	0.68	81	18	27	20	16	48.2
4.32	K_soil ≤ 207.5 ∧ Dryness =average ∧ B_soil > 550.8	0.56	0.73	32	9	12	9	2	47.7
4.32	S_soil > 11.4 ∧ Dryness ≠wet ∧ K_soil ≤ 234.0	0.29	0.67	98	32	19	30	17	51.4
4.31	S_soil > 11.4 ∧ Dryness =average ∧ P_soil ≤ 1.3	0.60	0.48	12	3	4	2	3	50.9
4.31	Dryness =average ∧ S_soil > 10.0 ∧ P_soil ≤ 1.3	0.60	0.48	12	3	4	2	3	50.9
4.31	Dryness =average ∧ B_soil > 241.2 ∧ B_soil > 432.0	0.40	0.70	57	13	15	16	13	49.1
4.31	Dryness =average ∧ B_soil > 346.8 ∧ B_soil > 432.0	0.40	0.70	57	13	15	16	13	49.1
4.30	Dryness =average ∧ K_soil ≤ 195.9 ∧ S_soil > 12.3	0.42	0.65	44	11	11	14	8	46.6
4.29	S_soil > 11.4 ∧ Dryness =average ∧ S_soil ≤ 36.6	0.30	0.59	73	16	17	18	22	47.5
4.28	Si_soil ≤ 8.4 ∧ Dryness =average ∧ Fe_soil > 210.0	0.56	0.61	22	10	5	1	6	51.2
4.27	Previously_cultivated_crop =grass ∧ Mn_soil > 8642.4 ∧ Dryness ≠average	0.57	0.38	8	3	0	1	4	53.9
4.25	B_soil > 252.0 ∧ Dryness =average ∧ S_soil ≤ 34.0	0.30	0.62	76	16	26	18	16	48.3
4.25	Dryness =average ∧ B_soil > 241.2 ∧ K_soil ≤ 302.1	0.33	0.68	77	17	26	20	14	47.9
4.24	P_soil > 7.4 ∧ K_soil ≤ 142.2 ∧ Si_soil > 7.0	0.58	0.39	8	1	5	1	1	50.9
4.23	B_soil > 252.0 ∧ Dryness =average ∧ K_soil ≤ 295.5	0.33	0.67	75	17	26	19	13	47.8
4.23	K_soil ≤ 146.1 ∧ Dryness =average ∧ Mn_soil > 2502.0	0.52	0.56	21	5	9	3	4	42.8
4.22	K_soil ≤ 207.5 ∧ Dryness =average ∧ S_soil > 12.8	0.41	0.66	47	12	12	14	9	46.4
4.21	Dryness =average ∧ K_soil ≤ 274.7 ∧ B_soil > 217.2	0.33	0.69	77	19	27	18	13	48.8
4.20	Dryness =average ∧ B_soil > 241.2 ∧ N_soil > 36.2	0.31	0.64	77	19	11	23	24	49.7
4.20	Dryness ≠wet ∧ S_soil > 10.8 ∧ K_soil ≤ 234.0	0.28	0.67	101	33	20	31	17	51.4
4.19	B_soil > 252.0 ∧ Dryness ≠wet ∧ K_soil ≤ 220.0	0.31	0.71	94	24	32	26	12	50.7
4.17	B_soil > 252.0 ∧ Dryness =average ∧ N_soil > 35.6	0.31	0.64	75	19	11	22	23	50.2
4.15	S_soil > 11.4 ∧ Dryness =average ∧ S_soil ≤ 26.5	0.31	0.55	55	11	16	14	14	47.8
4.15	Nutrient_content ≠average ∧ Si_soil > 12.4 ∧ Ca_soil ≤ 76.2	0.68	0.52	10	0	5	3	2	37.1
4.14	B_soil > 366.0 ∧ Dryness =average ∧ B_soil > 458.4	0.39	0.70	54	13	13	15	13	49.4
4.12	Dryness =average ∧ S_soil > 10.0 ∧ S_soil ≤ 35.2	0.28	0.59	76	17	18	19	22	47.6
4.11	Dryness =average ∧ B_soil > 241.2 ∧ S_soil ≤ 34.0	0.29	0.62	78	16	27	19	16	48.2
4.10	K_soil ≤ 207.5 ∧ Dryness =average ∧ B_soil > 195.6	0.38	0.73	63	14	25	15	9	47.1
4.10	B_soil > 564.0 ∧ N_soil > 174.2 ∧ B_soil ≤ 1084.8	0.46	0.48	19	2	1	6	10	45.3
4.09	S_soil ≤ 34.0 ∧ Dryness =average ∧ B_soil > 223.2	0.28	0.63	81	17	28	19	17	48.6

Yield is reported in ton ha^{-1}, N, P, K, Ca and Mg are reported in kg ha^{-1} and B, Fe, Mn and Zn are reported g ha^{-1}.
