# Supplementary material for: Introducing exceptional growth mining—Analyzing the impact of soil characteristics on on-farm crop growth and yield variability
Source: PLoS One. 2024 Jan 29;19(1):e0296684. doi: 10.1371/journal.pone.0296684 (PMC10824435; doi:10.1371/journal.pone.0296684)
Supplement: S5 Table — (PDF) [file pone.0296684.s007.pdf]

$\varphi^u_{\mathrm{GC}_h}$	Description	Mean	Std	Number of fields					Yield
				Total	2015	2016	2017	2018	
3.59	Dryness =wet $\wedge$ Nematodes $\neq$no	-0.38	0.26	6	5	0	1	0	66.5
3.54	B_soil $\leq$ 366.0 $\wedge$ Dryness =wet	-0.30	0.44	26	7	1	6	12	50.2
3.43	B_soil $\leq$ 564.0 $\wedge$ Dryness =wet	-0.34	0.66	43	8	8	8	19	44.4
3.24	S_soil $\leq$ 11.4 $\wedge$ Dryness =wet	-0.59	0.60	11	2	3	3	3	49.2
3.00	Dryness =wet $\wedge$ N_soil $\leq$ 89.8	-0.30	0.59	35	5	10	13	7	45.4
2.99	Dryness =wet $\wedge$ B_soil $\leq$ 750.0	-0.27	0.65	52	9	9	10	24	43.2
2.98	Zn_soil $\leq$ 1396.8 $\wedge$ Ca_soil > 249.1	-0.45	0.48	10	0	3	2	5	46.2
2.81	Ca_soil > 196.8 $\wedge$ Zn_soil $\leq$ 2055.6	-0.35	0.55	19	0	7	4	8	45.1
2.79	Dryness =wet $\wedge$ Zn_soil $\leq$ 1290.0	-0.45	0.68	18	2	5	5	6	46.6
2.76	S_soil $\leq$ 34.0 $\wedge$ Dryness =wet	-0.24	0.70	65	9	15	16	25	43.5
2.60	Dryness =wet $\wedge$ B_soil $\leq$ 486.0	-0.27	0.62	36	7	4	8	17	45.7
2.49	Fe_soil > 324.0 $\wedge$ Fe_soil $\leq$ 444.0	-0.21	0.57	47	10	12	12	13	49.0
2.41	Dryness =wet $\wedge$ Si_soil $\leq$ 15.0	-0.19	0.67	70	10	12	22	26	46.2
2.40	Mg_soil $\leq$ 158.5 $\wedge$ Zn_soil $\leq$ 2829.6	-0.32	0.46	12	2	1	2	7	44.6
2.38	Dryness =wet $\wedge$ Si_soil $\leq$ 12.0	-0.22	0.68	53	10	8	18	17	47.9
2.30	Dryness =wet $\wedge$ S_soil $\leq$ 38.0	-0.20	0.72	69	10	16	18	25	43.8
2.24	Ca_soil > 196.8 $\wedge$ Ca_soil > 442.1	-0.28	0.39	10	0	1	5	4	51.1
2.23	Nutrient_content =rich $\wedge$ Fe_soil $\leq$ 162.0	-0.29	0.41	10	2	3	5	0	61.5
2.20	Dryness =wet $\wedge$ Fe_soil $\leq$ 594.0	-0.20	0.75	69	8	14	21	26	44.8
2.01	Nutrient_content =rich $\wedge$ Si_soil $\leq$ 7.2	-0.24	0.40	11	3	3	2	3	54.6
1.94	B_soil > 1113.6 $\wedge$ B_soil $\leq$ 1398.0	-0.22	0.41	13	1	4	7	1	53.8
1.90	N_soil $\leq$ 220.8 $\wedge$ Dryness =wet	-0.17	0.76	74	10	17	20	27	43.7
1.89	N_soil $\leq$ 40.2 $\wedge$ Mn_soil $\leq$ 470.4	-0.37	0.67	12	0	7	3	2	43.7
1.80	N_soil $\leq$ 40.2 $\wedge$ Dryness $\neq$average	-0.18	0.54	30	2	16	6	6	49.1
1.77	P_soil $\leq$ 7.4 $\wedge$ Dryness =wet	-0.16	0.77	77	10	17	22	28	44.4
1.76	Dryness =wet $\wedge$ P_soil $\leq$ 18.1	-0.14	0.76	85	12	18	24	31	44.9
1.71	Nutrient_content =rich $\wedge$ K_soil $\leq$ 82.5	-0.21	0.38	10	4	3	3	0	53.4
1.66	P_soil > 7.4 $\wedge$ Mg_soil > 285.2	-0.18	0.38	12	4	0	6	2	58.9

Yield is reported in ton ha^{-1}, N, P, K, Ca and Mg are reported in kg ha^{-1} and B, Fe, Mn and Zn are reported g ha^{-1}.
