# Supplementary material for: Introducing exceptional growth mining—Analyzing the impact of soil characteristics on on-farm crop growth and yield variability
Source: PLoS One. 2024 Jan 29;19(1):e0296684. doi: 10.1371/journal.pone.0296684 (PMC10824435; doi:10.1371/journal.pone.0296684)
Supplement: S6 Table — (PDF) [file pone.0296684.s008.pdf]

$\varphi^u_{\mathrm{GC}_h}$	Description	Mean	Std	Number of fields Total	2015	2016	2017	2018	Yield
6.74	Previously_cultivated_crop =sugar_beet ∧ Si_soil > 10.0	0.58	0.23	7	2	2	1	2	51.2
5.29	K_soil > 308.1 ∧ Ca_soil > 439.6	0.45	0.28	11	0	2	5	4	52.2
5.26	Zn_soil ≤ 1396.8 ∧ Ca_soil > 249.1	0.53	0.32	10	0	3	2	5	46.2
4.82	Ca_soil > 196.8 ∧ Ca_soil > 442.1	0.46	0.30	10	0	1	5	4	51.1
4.49	Previously_cultivated_crop =sugar_beet ∧ Si_soil > 8.8	0.42	0.33	12	4	2	3	3	54.6
4.49	Si_soil > 8.4 ∧ Previously_cultivated_crop =sugar_beet	0.42	0.33	12	4	2	3	3	54.6
3.43	Fe_soil > 324.0 ∧ P_soil > 6.0	0.32	0.44	22	10	6	3	3	55.0
3.42	Fe_soil > 472.8 ∧ P_soil > 4.9	0.50	0.50	12	6	4	2	0	55.8
3.33	Zn_soil > 7294.8 ∧ B_soil ≤ 592.8	0.26	0.47	36	7	11	6	12	46.7
3.01	K_soil ≤ 146.1 ∧ Ca_soil > 14.7	0.37	0.53	18	0	10	1	7	41.1
2.93	Mn_soil > 1454.4 ∧ Mg_soil ≤ 141.5	0.28	0.58	36	7	11	6	12	44.7
2.77	Zn_soil > 3906.0 ∧ Mg_soil ≤ 133.2	0.27	0.47	24	4	8	3	9	41.5
2.74	Ca_soil > 1.4 ∧ N_soil ≤ 34.2	0.23	0.58	48	0	32	7	9	45.7
2.74	Ca_soil ≤ 994.3 ∧ N_soil ≤ 34.0	0.23	0.58	48	0	32	7	9	45.7
2.64	K_soil > 207.5 ∧ Zn_soil > 7654.8	0.23	0.42	24	4	1	11	8	55.4
2.63	Zn_soil > 7294.8 ∧ B_soil ≤ 366.0	0.24	0.46	25	6	6	4	9	45.3
2.62	K_soil ≤ 96.4 ∧ N_soil ≤ 28.0	0.27	0.37	13	1	8	2	2	49.0
2.59	Zn_soil > 7294.8 ∧ K_soil > 215.0	0.24	0.44	23	4	0	11	8	54.4
2.58	Previously_cultivated_crop =sugar_beet ∧ S_soil > 14.1	0.27	0.41	16	6	2	3	5	54.3
2.58	N_soil ≤ 40.2 ∧ K_soil ≤ 111.2	0.27	0.52	24	2	14	4	4	46.8
2.54	Mn_soil > 3123.6 ∧ Mg_soil ≤ 129.8	0.23	0.45	24	4	8	2	10	40.0
2.50	S_soil ≤ 11.4 ∧ Ca_soil > 1.4	0.23	0.64	49	0	27	7	15	45.0
2.50	Ca_soil > 1.4 ∧ S_soil ≤ 11.2	0.23	0.64	49	0	27	7	15	45.0
2.50	Ca_soil ≤ 994.3 ∧ S_soil ≤ 11.2	0.23	0.64	49	0	27	7	15	45.0
2.47	S_soil ≤ 11.4 ∧ Ca_soil > 6.2	0.31	0.67	29	0	19	2	8	42.2
2.45	Ca_soil ≤ 196.8 ∧ N_soil ≤ 31.0	0.22	0.56	40	0	27	6	7	47.3
2.41	Zn_soil ≤ 2706.0 ∧ Ca_soil > 247.0	0.36	0.65	19	0	7	3	9	44.0
2.37	S_soil ≤ 11.4 ∧ Si_soil > 9.0	0.34	0.63	20	2	9	4	5	46.7
2.34	Ca_soil ≤ 60.6 ∧ N_soil ≤ 35.4	0.20	0.45	29	0	16	6	7	46.6
2.29	Ca_soil > 196.8 ∧ Zn_soil ≤ 2055.6	0.33	0.63	19	0	7	4	8	45.1
2.25	N_soil ≤ 40.2 ∧ Ca_soil > 1.4	0.19	0.64	55	0	35	11	9	46.2
2.24	S_soil ≤ 11.4 ∧ Mg_soil ≤ 274.8	0.20	0.64	49	10	21	4	14	47.8
2.22	Mg_soil ≤ 158.5 ∧ Ca_soil ≤ 224.9	0.20	0.56	37	0	11	9	17	43.8
2.22	B_soil ≤ 1113.6 ∧ Zn_soil > 7232.4	0.15	0.48	48	9	13	12	14	47.9
2.20	Fe_soil > 324.0 ∧ N_soil ≤ 34.4	0.32	0.69	23	1	16	4	2	43.9
2.16	N_soil ≤ 40.2 ∧ N_soil ≤ 32.0	0.18	0.57	49	3	31	7	8	47.6
2.16	N_soil ≤ 87.0 ∧ N_soil ≤ 32.4	0.18	0.57	49	3	31	7	8	47.6
2.16	N_soil ≤ 220.8 ∧ N_soil ≤ 32.0	0.18	0.57	49	3	31	7	8	47.6

Yield is reported in ton ha^{-1}, N, P, K, Ca and Mg are reported in kg ha^{-1} and B, Fe, Mn and Zn are reported g ha^{-1}.
