# Supplementary material for: Introducing exceptional growth mining—Analyzing the impact of soil characteristics on on-farm crop growth and yield variability
Source: PLoS One. 2024 Jan 29;19(1):e0296684. doi: 10.1371/journal.pone.0296684 (PMC10824435; doi:10.1371/journal.pone.0296684)
Supplement: S7 Table — (PDF) [file pone.0296684.s009.pdf]

$\varphi^{u}_{\mathrm{GC}_h}$	Description	Mean	Std	Number of fields					Yield
				Total	2015	2016	2017	2018	
4.44	Si_soil $\leq$ 6.0 $\wedge$ Mg_soil $>$ 284.3	-0.63	0.51	13	5	6	1	1	53.4
4.28	Mg_soil $>$ 280.6 $\wedge$ Si_soil $\leq$ 6.0	-0.59	0.51	14	5	6	1	2	52.6
3.81	N_soil $>$ 40.2 $\wedge$ K_soil $\leq$ 234.0	-0.21	0.67	146	45	25	42	34	49.2
3.76	K_soil $\leq$ 207.5 $\wedge$ N_soil $>$ 30.0	-0.22	0.71	143	42	28	41	32	48.2
3.71	N_soil $>$ 40.2 $\wedge$ Mg_soil $>$ 202.2	-0.21	0.68	141	37	17	42	45	50.2
3.56	Zn_soil $\leq$ 3906.0 $\wedge$ Mg_soil $>$ 228.0	-0.24	0.68	104	27	21	24	32	49.8
3.54	Fe_soil $\leq$ 324.0 $\wedge$ K_soil $\leq$ 216.3	-0.24	0.70	109	20	30	31	28	48.9
3.38	K_soil $\leq$ 146.1 $\wedge$ B_soil $>$ 1405.2	-0.62	0.90	24	4	7	13	0	46.4
3.36	Nutrient_content =rich $\wedge$ Mn_soil $\leq$ 1506.0	-0.27	0.35	19	4	7	4	4	49.7
3.29	Si_soil $\leq$ 8.4 $\wedge$ Mn_soil $\leq$ 610.8	-0.34	0.51	24	9	6	1	8	54.6
3.28	Mg_soil $>$ 199.2 $\wedge$ Dryness $\neq$dry	-0.19	0.66	125	26	25	34	40	49.3
3.28	Dryness $\neq$dry $\wedge$ Mg_soil $>$ 200.6	-0.19	0.66	125	26	25	34	40	49.3
3.25	K_soil $\leq$ 207.5 $\wedge$ N_soil $>$ 109.0	-0.27	0.69	71	21	13	21	16	50.8
3.23	Mg_soil $>$ 199.2 $\wedge$ Dryness =average	-0.22	0.60	77	19	16	20	22	51.4
3.22	S_soil $>$ 11.4 $\wedge$ Dryness $\neq$dry	-0.17	0.69	167	30	33	47	57	47.0
3.22	S_soil $>$ 22.8 $\wedge$ K_soil $\leq$ 178.4	-0.37	0.78	46	14	7	20	5	50.6
3.20	Mg_soil $>$ 238.6 $\wedge$ K_soil $\leq$ 350.6	-0.22	0.67	95	33	15	21	26	50.7
3.20	N_soil $>$ 40.2 $\wedge$ Mg_soil $>$ 244.2	-0.21	0.65	95	34	9	24	28	51.8
3.19	Dryness $\neq$dry $\wedge$ N_soil $>$ 43.4	-0.16	0.66	170	40	25	42	63	48.1
3.16	Mn_soil $\leq$ 717.6 $\wedge$ Nematodes =yes	-0.41	0.53	17	4	5	0	8	51.1
3.16	Mn_soil $\leq$ 717.6 $\wedge$ Nematodes $\neq$no	-0.41	0.53	17	4	5	0	8	51.1
3.16	B_soil $>$ 564.0 $\wedge$ K_soil $\leq$ 189.4	-0.29	0.77	71	16	20	26	9	48.8
3.16	S_soil $>$ 16.8 $\wedge$ K_soil $\leq$ 167.7	-0.30	0.80	69	20	15	26	8	48.8
3.16	N_soil $>$ 40.2 $\wedge$ Zn_soil $\leq$ 7192.8	-0.16	0.71	189	43	29	51	66	48.5
3.14	N_soil $>$ 40.2 $\wedge$ Dryness $\neq$dry	-0.16	0.67	172	40	26	43	63	48.0
3.13	Dryness =average $\wedge$ Zn_soil $\leq$ 6868.8	-0.19	0.60	97	25	20	22	30	50.5
3.12	N_soil $>$ 40.2 $\wedge$ Mg_soil $>$ 158.5	-0.16	0.69	189	44	30	56	59	49.4
3.12	Mg_soil $>$ 158.5 $\wedge$ N_soil $>$ 39.4	-0.16	0.69	189	44	30	56	59	49.4
3.11	Mn_soil $\leq$ 7648.8 $\wedge$ Mg_soil $>$ 218.3	-0.17	0.65	142	34	25	38	45	49.7
3.10	Mn_soil $\leq$ 7648.8 $\wedge$ N_soil $>$ 37.0	-0.16	0.70	187	45	28	52	62	48.7
3.10	Zn_soil $\leq$ 3906.0 $\wedge$ Zn_soil $>$ 2518.8	-0.28	0.77	71	16	13	14	28	46.6
3.08	N_soil $>$ 40.2 $\wedge$ K_soil $\leq$ 346.1	-0.15	0.70	192	52	28	56	56	48.8
3.07	Mn_soil $\leq$ 717.6 $\wedge$ Mn_soil $>$ 463.2	-0.44	0.71	24	7	5	3	9	49.0
3.06	S_soil $>$ 22.8 $\wedge$ K_soil $\leq$ 257.0	-0.27	0.73	70	20	10	28	12	51.7
3.04	Nematodes $\neq$yes $\wedge$ B_soil $>$ 1164.0	-0.40	0.83	41	7	12	19	3	50.6
3.04	Nematodes =no $\wedge$ B_soil $>$ 1164.0	-0.40	0.83	41	7	12	19	3	50.6
3.03	Si_soil $\leq$ 8.4 $\wedge$ Dryness =average	-0.26	0.63	56	13	15	10	18	49.1
3.03	Mg_soil $>$ 199.2 $\wedge$ Mg_soil $>$ 228.0	-0.17	0.65	138	37	22	39	40	50.9
3.03	Mg_soil $>$ 158.5 $\wedge$ Mg_soil $>$ 228.0	-0.17	0.65	138	37	22	39	40	50.9
3.01	S_soil $>$ 11.4 $\wedge$ Previously_cultivated_crop $\neq$sugar_beet	-0.14	0.70	219	42	40	69	68	48.1
3.01	Previously_cultivated_crop $\neq$sugar_beet $\wedge$ S_soil $>$ 11.2	-0.14	0.70	219	42	40	69	68	48.1
3.00	Mg_soil $>$ 280.6 $\wedge$ Fe_soil $\leq$ 307.2	-0.33	0.67	36	9	9	9	9	53.2
3.00	Mn_soil $\leq$ 3123.6 $\wedge$ Mg_soil $>$ 226.1	-0.19	0.65	107	28	21	24	34	50.5
2.99	Dryness =average $\wedge$ Mg_soil $>$ 203.4	-0.21	0.59	73	18	14	20	21	51.1
2.98	Zn_soil $\leq$ 3906.0 $\wedge$ Mn_soil $>$ 2516.4	-0.42	0.84	36	8	7	12	9	44.2
2.98	K_soil $\leq$ 207.5 $\wedge$ Mg_soil $>$ 238.0	-0.24	0.69	71	25	14	16	16	51.1
2.98	B_soil $>$ 1113.6 $\wedge$ K_soil $\leq$ 165.5	-0.42	0.85	36	6	12	17	1	47.3
2.97	Dryness $\neq$dry $\wedge$ Nutrient_content $\neq$poor	-0.13	0.67	237	43	70	52	72	46.7
2.97	Nutrient_content $\neq$poor $\wedge$ Dryness $\neq$dry	-0.13	0.67	237	43	70	52	72	46.7
2.96	Si_soil $\leq$ 6.0 $\wedge$ Mg_soil $>$ 230.6	-0.35	0.60	25	8	8	6	3	55.5
2.95	P_soil $\leq$ 4.4 $\wedge$ Mg_soil $>$ 280.8	-0.34	0.70	36	10	6	10	10	50.6
2.93	Si_soil $\leq$ 8.4 $\wedge$ Previously_cultivated_crop $\neq$maize	-0.26	0.57	41	12	11	6	12	52.3
2.92	Dryness =average $\wedge$ N_soil $>$ 33.0	-0.19	0.63	100	28	13	26	33	51.0

Yield is reported in ton ha^{-1}, N, P, K, Ca and Mg are reported in kg ha^{-1} and B, Fe, Mn and Zn are reported g ha^{-1}.
